# Geospatial indicators of exposure, sensitivity, and adaptive capacity to assess neighbourhood variation in vulnerability to climate change-related health hazards

**DOI:** 10.1186/s12940-021-00708-z

**Published:** 2021-03-22

**Authors:** Jessica Yu, Kaitlin Castellani, Krista Forysinski, Paul Gustafson, James Lu, Emily Peterson, Martino Tran, Angela Yao, Jingxuan Zhao, Michael Brauer

**Affiliations:** 1 School of Population and Public Health, The University of British Columbia (UBC), 2206 East Mall, Vancouver, British Columbia V6T 1Z3 Canada; 2 Faculty of Forestry, The University of British Columbia, Forest Sciences Centre, 2424 Main Mall, Vancouver, BC V6T 1Z4 Canada; 3 Institute for Resources, Environment and Sustainability, The University of British Columbia, 429-2202 Main Mall, Vancouver, British Columbia V6T 1Z3 Canada; 4 Department of Statistics, The University of British Columbia, 3182 Earth Sciences Building, 2207 Main Mall, Vancouver, British Columbia V6T 1Z3 Canada; 5 Vancouver Coastal Health, 601 West Broadway, 11th floor, Vancouver, British Columbia V5Z 4C2 Canada; 6 School of Community and Regional Planning, The University of British Columbia, 433 - 6333 Memorial Road, Vancouver, British Columbia V6T 1Z3 Canada; 7 Faculty of Medicine, The University of British Columbia, 317 - 2194 Health Sciences Mall, Vancouver, British Columbia V6T 1Z3 Canada

**Keywords:** Vulnerability mapping, Climate change and health, Index scores, Adaptive capacity, Principal component analysis

## Abstract

**Background:**

Although the frequency and magnitude of climate change-related health hazards (CCRHHs) are likely to increase, the population vulnerabilities and corresponding health impacts are dependent on a community’s exposures*,* pre-existing sensitivities, and adaptive capacities in response to a hazard’s impact. To evaluate spatial variability in relative vulnerability, we: 1) identified climate change-related risk factors at the dissemination area level; 2) created actionable health vulnerability index scores to map community risks to extreme heat, flooding, wildfire smoke, and ground-level ozone; and 3) spatially evaluated vulnerability patterns and priority areas of action to address inequity.

**Methods:**

A systematic literature review was conducted to identify the determinants of health hazards among populations impacted by CCRHHs. Identified determinants were then grouped into categories of exposure, sensitivity, and adaptive capacity and aligned with available data. Data were aggregated to 4188 Census dissemination areas within two health authorities in British Columbia, Canada. A two-step principal component analysis (PCA) was then used to select and weight variables for each relative vulnerability score. In addition to an overall vulnerability score, exposure, adaptive capacity, and sensitivity sub-scores were computed for each hazard. Scores were then categorised into quintiles and mapped.

**Results:**

Two hundred eighty-one epidemiological papers met the study criteria and were used to identify 36 determinant indicators that were operationalized across all hazards. For each hazard, 3 to 5 principal components explaining 72 to 94% of the total variance were retained. Sensitivity was weighted much higher for extreme heat, wildfire smoke and ground-level ozone, and adaptive capacity was highly weighted for flooding vulnerability. There was overall varied contribution of adaptive capacity (16–49%) across all hazards. Distinct spatial patterns were observed – for example, although patterns varied by hazard, vulnerability was generally higher in more deprived and more outlying neighbourhoods of the study region.

**Conclusions:**

The creation of hazard and category-specific vulnerability indices (exposure, adaptive capacity and sensitivity sub-scores) supports evidence-based approaches to prioritize public health responses to climate-related hazards and to reduce inequity by assessing relative differences in vulnerability along with absolute impacts. Future studies can build upon this methodology to further understand the spatial variation in vulnerability and to identify and prioritise actionable areas for adaptation.

**Supplementary Information:**

The online version contains supplementary material available at 10.1186/s12940-021-00708-z.

## Introduction and background

Climate change is projected to increase the likelihood of flooding, extreme heat, wildfire smoke, and ozone events affecting communities around the world – reshaping the sustainability, health, and well-being of these communities and their surrounding areas [1, 2]. Many cities can be at-risk for inland flooding, when the volume of water on land is greater than the capacity of the natural and built drainage systems. This can be a result of heavy precipitation, snow melt, and the failure of dams and levees. In addition, sea-level rise can specifically impact coastal communities, particularly during storm events. Floods can often wreak havoc on populations and their homes and leave devastating long-term health, livelihood and financial consequences. Further, many cities are rapidly urbanising and experiencing more frequent extreme heat events, when summertime and nighttime temperatures are much hotter and humid than average; August 2020, for example, was the fourth warmest month on record worldwide as global temperatures increased 0.9 degrees Celsius above historical averages [3]. With increasing temperatures, we have also seen an uptick of heat related diseases and mortality [4–6]. Warmer temperatures and changes in precipitation patterns have also lengthened and worsened wildfire seasons in North America, leading to increased smoke events in recent summers [7]. Wildfire smoke is a mixture of gaseous and particle air pollution, including fine particulate matter (PM2.5). When inhaled, even over short periods, it can exacerbate pre-existing health conditions, such as chronic obstructive pulmonary disease, heart diseases, and diabetes. Finally, ground-level ozone is an air pollutant that is formed by photochemical reactions during warm daytime conditions. Climate change is expected to increase the frequency and magnitude of ozone episodes. Ozone exposure, can lead to premature mortality and respiratory emergency room visits and hospitalization [8]. The Intergovernmental Panel on Climate Change (IPCC) Special Report on Extreme Events and Disasters (2012) cautioned that climate change will increase the magnitude, frequency, duration and spatial extent of weather-related events, presenting greater future population health challenges [2]*.*

Although the frequency and magnitude of these hazards are likely to increase, the population vulnerability risk factors and corresponding health impacts are dependent on the combined elements of: hazard exposures (forces or shocks, including flooding or extreme heat events projected to increase with climate change), a community’s pre-existing sensitivities (intrinsic characteristics or conditions that make people more likely to be affected by the exposures, such as age or having pre-existing health conditions), and adaptive capacities (individual and collective resources to adjust to actual or expected climate and its effects, including material or social resources to help moderate harms and exploit beneficial opportunities) [9, 10]*.* Risk factors, or determinants, are defined as any attribute, characteristic or exposure of a household or community that increases the likelihood of developing a disease or injury from the identified climate change-related hazards. These vulnerabilities may be spatially variable, meaning that flooding from rainfall or sea level rise (SLR) can impact some populations more than others for example, depending on where homes are situated and the community’s storm management capacity, while a heat wave can have differential impacts on neighbourhoods depending on the number of seniors or children living in these communities. Therefore, understanding the local context is increasingly important, as larger countries can have geographically diverse weather patterns and variability in community vulnerability profiles and adaptation guidelines. A review of municipal heat emergency response plans indicates that many such plans have not adequately addressed vulnerability of at-risk populations from emerging climate threats [11, 12].

In response, public health authorities around the world have conducted vulnerability and adaptation assessments to assist with local service planning and prioritisation. However, these assessments have been impeded by limited local-level data and models that integrate health, climate, and climate projections down to the neighbourhood or sub-neighbourhood levels. Although there are often cross-disciplinary collaborations between public health departments in climate adaptation planning, some researchers have argued that there are still insufficient health vulnerability and impact assessments for proposed infrastructure projects, especially with a health equity focus [13, 14]. This can include approved housing, transportation, and/or health infrastructure projects that have not comprehensively assessed the health impacts due to climate change. Although the disaster risk community has been collecting information on vulnerability assessments for decades, more evidence-based and data-driven approaches are needed to help identify communities at smaller spatial scales that are disproportionately affected by emerging hazard events [15]; these exposures, sensitivities, and adaptive capacities may vary widely within municipalities or between neighbourhood blocks. Combining multiple indicators to operationalise priority risk factors into composite measures for geospatial mapping provides opportunities to inform resource allocation, but many current composite measures have been conceptualised broadly without providing actionable information about which interventions are more likely to reduce vulnerability that are within the purview and resources of local planners. Arguably, more pragmatic indicators that identify levels and determinants of variability and which can be used for monitoring and evaluation are needed to inform planners on the interventions that would reduce vulnerability in an equitable manner.

Most recent hazard indicators have generally followed the inductive methodology of Cutter et al. to quantitatively map vulnerability indices [16]. A review of vulnerability indicators revealed that indicators in the past have been constructed with a variety of approaches including expert judgment [17], multi-criteria decision analysis [18], equal weighting, ordered weighted averaging [19], unweighted standardisation [17], analytic hierarchy process [18], and multivariate statistical techniques, such as principal components [17] or cluster analysis [20]. Johnson and colleagues argue that the community vulnerability and resilience literature is lacking data-driven methodologies that are more conducive to empirical validation compared to theory-driven philosophies [21]. Future data-driven vulnerability indices may benefit from the selection of indicators and weights-based references, local contextual information, and relevant hazard-related health outcomes [20].

In addition, while many previous vulnerability assessments have incorporated demographic and socioeconomic indicators to measure social vulnerability [22, 23], fewer have combined multiple social and physical factors for local-level planning [24]. Notwithstanding that global and national level differences in climate change related hazards are relatively well understood, within a defined geography in a single climate zone such as a metropolitan area, impacts from climate change and the ability to adapt to these conditions will vary between and within neighbourhoods. Therefore, assessments at the subnational and neighbourhood level provide opportunities to identify and address locally-specific risk factors that may otherwise be overlooked at broader national, provincial, or municipal scales [15]. Some intra-urban studies have been conducted in North America [25–31], but fewer have been done from a local public health perspective to assess variability in relative vulnerability, and not absolute health impacts, of climate change. Overall, more analytic research and vulnerability assessments that provide more actionable insight [32] using public health and spatial equity lenses are required for local service planning and prioritization.

In conjunction with regional public health authorities, we initiated an assessment of differences in relative, and not absolute, vulnerability from climate change-related hazards within two regions of British Columbia, Canada. The overarching objectives were to: 1) identify climate change-related risk factors at the *local* community level; 2) create *actionable* health vulnerability index scores to map community risks to previously identified priority climate-related exposures: flooding (in this study including both inland flooding and that due to SLR), extreme heat, wildfire smoke, and ozone; and 3) spatially assess common areas of vulnerability and *priority areas of action* in the study area context. We outline the methodology developed and applied for this assessment and present opportunities for application of the developed indices and visualization tools.

## Methods

### Study setting: British Columbia

British Columbia (BC) is one of the most physically and biologically diverse provinces in Canada, with many different climatic regions. Thus, the province makes for an ideal study of regional and local variation in vulnerability because weather in one region (the coastal temperate rainforest, for example) is not indicative of other regions (the semi-arid desert of the interior Okanagan Valley, for example). The potential impacts from the increasing number of climate-change related events (CCREs) are diverse and uncertain. However, extreme heat, inland flooding and SLR, wildfire smoke, and ground level ozone have recently been prioritized [33, 34] in this province. In the last century, average temperature in the province has increased 1.4 degrees Celsius, which is higher than the global average (0.85 degrees Celsius) [35]. In 2018, BC recorded its warmest year on record, instated the longest state of emergency due to wildfire smoke in the history of BC, and observed water levels reaching historical highs [36]. Major cities within the province, such as Vancouver, are susceptible to localised flash flooding from heavy rainfall and insufficient drainage systems [37], and more than 250,000 people live within a meter of mean sea level, making it the most vulnerable urban center in Canada to SLR [38].

Despite these emergent threats and concerns of CCREs, the province’s internal audit released in February 2018 revealed that BC lacks climate change adaptation policies, especially related to wildfires and flooding [34]. Although the province released an adaptation strategy in 2010, the auditor general highlighted that little has been done to monitor the progress and reporting on climate action performance, a comprehensive risk assessment has not been completed, nor have climate-driven risks been prioritised across the province. Since then, a preliminary strategic climate risk assessment has been published to understand province-scale climate risks [33]. Without more spatially granular information, local governments, who play a key role in the adaptation of greenhouse gas emissions, lack financial support, reliable data, and knowledge to adapt to CCRE to support their communities. Within a region, there is variability in the types of CCRE that specific communities are vulnerable to, hence high-resolution data mapping inter-community variation can provide guidance in which areas to direct limited resources to. Overall, Vancouver and other cities in Canada would benefit from more mapping of risks [39]. Accordingly, this study responds to the current local and international calls to assess climate change-related risk factors at the neighbourhood level. We focused on two BC health regions, home to more than 2.8 million people.

## Material and methods

### Systematic literature review

**Fig. 1:**
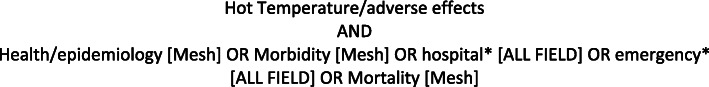
Example inclusion criteria - extreme heat

A systematic literature review was conducted to identify the determinants of health hazards among populations impacted by four target climate-related hazards: extreme heat, flooding, wildfire smoke, and ground level ozone. These determinants are defined as any attribute, characteristic or exposure of a household or community that increases the likelihood of developing a disease or injury from climate change-related hazards; they can ultimately affect the frequency and spatial patterns with which the impacts of these hazards are observed at the population level. An example search strategy for extreme heat can be seen in Fig. 1, which includes the inclusion criteria. More detailed search strategies are provided in Additional file 1. Based upon the search we extracted from each study: outcomes, data sources, key methods, and a tabulation of all determinants mentioned. A second reader reviewed the final set of papers to corroborate the results.

Each list of determinants was reviewed and aggregated into common categories to be able to assess comparisons between all climate hazards. Common categories were decided as a research group until agreement was reached with all members. A data-driven approach was then used to assess categories and determinants that were mentioned the most frequently. If a determinant was identified in the analysis or mentioned by the authors as a potential underlying cause, it was added to the tabulation. There were two readers for each climate hazard systematic review; each reader identified the determinants independently and compared their findings until agreement was reached. The determinants were then grouped into sub-categories (climate, age, preexisting health conditions, socioeconomic status, race/ethnicity, built environment, social cohesion, and institutional) and then into broader categories that were based upon the common conceptual vulnerability framework in the literature (exposure, sensitivity, adaptive capacity) [40]. This conceptualisation is also used within the World Health Organisation (WHO) Framework for Building Climate Resilient Health Systems [1], a document intended to support health decision-makers in their efforts to instill climate-resilience within health systems globally. The tabulations were summed to identify the determinants that were mentioned in the literature most frequently (the top 2–3 determinants in each sub-category if there were many in the literature, or the leading determinant for each sub-category if there were less than 3 determinants identified from the literature).

Results of the tabulation were shared with stakeholders for expert judgement of appropriateness of categories and indicators, and to determine whether data was available to assess these determinants in the study area. The stakeholders engaged were primarily from the health sector. Appropriateness of the determinant categories and indicators for the local context was defined as agreement from more than one stakeholder and also identified from the literature review.

### Data collection and preparation

Potential data sources were collected into a repository. The Vancouver Coastal Health (VCH) and Fraser Health (FH) authority boundaries are comprised of 4188 Census dissemination areas, 158 municipalities, and 36 *My Health, My Community *survey neighbourhoods. All data were aggregated to the dissemination area (DA) level within the two health authority boundaries using ArcMap (v. 10.6) and Microsoft Excel. In Canada, dissemination areas are the smallest standard geographic areas for which census data are publicly available, with populations of 400–700 persons. Due to privacy concerns associated with reporting census data in small areas, some less populous DAs had missing census values. These data gaps were addressed by 1) replacing missing values with an average of the corresponding census tract’s values, 2) where the census tract data were also missing, an average of the corresponding Aggregate Dissemination Area (ADA) (the next level of geographic aggregation in the census) values were instead used, or 3) where the ADA also had no data reported, missing values were replaced by the average of values from the nearest ADA.

In total, 36 variables were collected from 10 different data sources (see Additional file 2 for more details on all variables and data sources). Some variables are worth noting in detail here. Not having access to medical services was considered if participants self-reported to not having a family doctor. Weak social network (respondents reported not having 4+ people to confide in/turn to for help) and emergency and displacement plans (households reported not having emergency supplies for 3+ days) were both from the *My Health, My Community* (MHMC) population surveys [41]. For municipalities where institutional guidelines were not available from the Resilience-C platform [42], a Google search was conducted using for search terms the municipality name and ‘flood planning guidelines’ to assess for official community plans, hazard risk and vulnerability assessment, and any major study done mentioned in the former documents. The variable was categorized as 1 (yes) or 0 (no). The results can be found in Additional file 3.

### Development of vulnerability indices for geospatial analysis

**Fig. 2:**
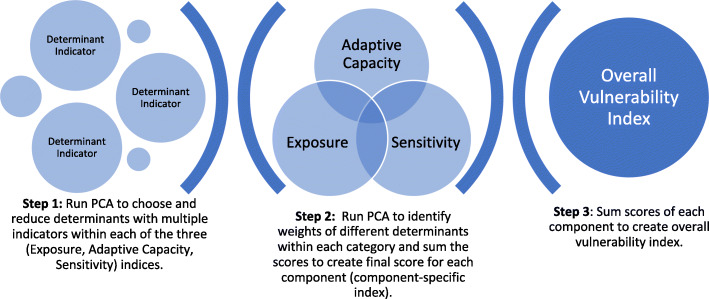
Multi-step process to create vulnerability scores

Principal component analysis (PCA) was applied as a practical data reduction method which allows identification of the individual vulnerability components (exposure, adaptive capacity, and sensitivity) within an overall vulnerability index (Fig. 2). This allowed for highlighting the overall variability within the region and the relative contribution of the different components to this variability. In this way, one can identify the corresponding determinants that may have the most impact on population health inequity related to CCRE.

All collected data were imported into R Studio (v. 1.1.453) and linked by DA IDs. To allow comparisons between determinant indicators, all measures were standardized by calculating z-scores ((indicator score – indicator mean)/standard deviation). Four criteria were used to retain meaningful components based on ‘best practices’ [43] and a similar heat vulnerability index [44]: 1) Eigenvalue > 1; 2) a Scree test to plot components against variance to assess the slope of the relationship and to identify breaks; 3) individual component variance > 10% or cumulative variance of at least 70%; and 4) interpretability criterion – loaded variables share similar concepts (e.g. depression and anxiety for mental health). A two-step process was then used to reduce, choose, and weight the variables for each vulnerability index: 1) if a determinant had multiple indicators (e.g. low income and low education for socioeconomic determinants), PCA using the PCA() function in R was run to select the variable that explained the most variance; 2) based on the results from the first PCA, a second PCA was run to identify the weights of the different determinant indicators within each category (exposure, sensitivity, and adaptive capacity). The category vulnerability scores were calculated by multiplying the chosen indicator for each determinant by its loading and each component by its corresponding eigenvalue. To identify the weights of each determinant, the percentage contribution of the determinant within each categorical index score can be computed as: (determinant indicator loading / total loading) * component eigenvalue.

**Fig. 3:**
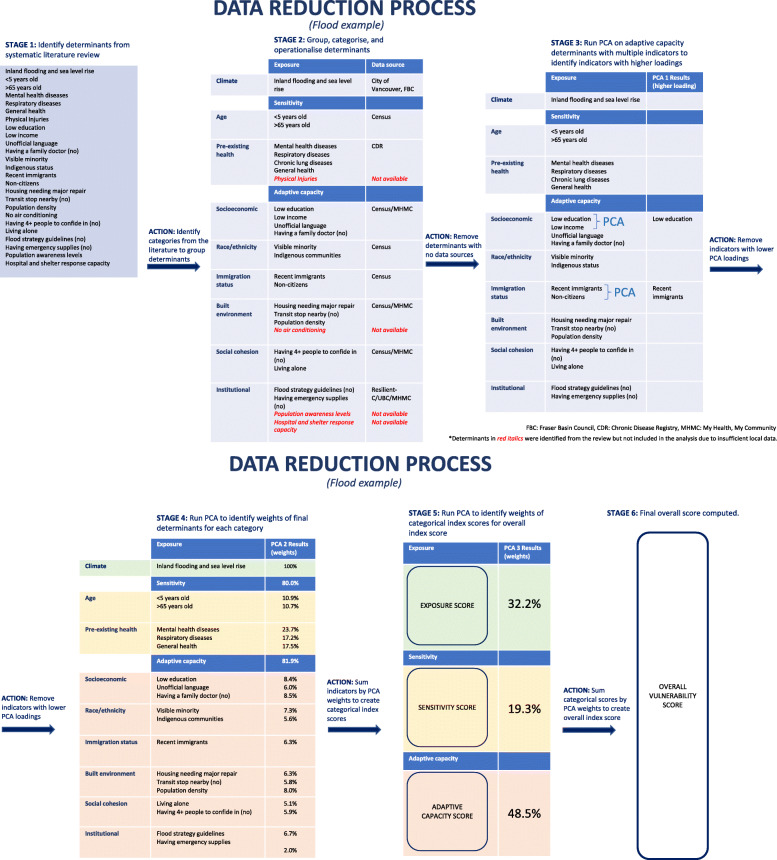
Summary of data reduction process (flooding example)

The final scores for each category were summed to create an overall index for each hazard (extreme heat, flooding, wildfire smoke, and ground-level ozone) and the data were re-scaled to range from 0 to 1. The index scores were then categorised into quintiles (very high, high, medium, low, and very low vulnerability) and mapped (QGIS Development Team, 2018) to show the relative distribution between different DAs within the VCH and FHA regions. Figure 3 is a summary of the data reduction process for flooding; figures for other hazards can be found in Additional file 4. Data were also loaded to an interactive visualization tool displaying vulnerability maps on ArcGIS Storymaps *(Vancouver Coastal Health Community Health and Climate Change)*. This interactive tool was used for knowledge dissemination to other stakeholders (municipalities and First Nations Health Authority) and to the wider public.

## Results

### Systematic review

One hundred two epidemiologic papers met inclusion criteria and were reviewed to identify determinants for extreme heat, 37 for flooding, 25 for wildfire smoke, and 117 for ozone, for a grand total of 281 epidemiological papers. Details of the search strategy and results are provided in Additional file 2. Table 1 summarises the results of the systematic review (Stage 1, in Fig. 3) after analysing the determinants that were identified most frequently and where relevant data sources were available. Many of these determinants were similarly identified in other systematic reviews [45–52], meta-analyses [53, 54], within specific Organisation for Economic Co-operation and Development (OECD) countries [55], within global urban analyses [56], in government reports [57] and weight-of-evidence analyses [58]. The findings in this review reflect epidemiological studies that were identified in the North American context.

**Table 1:** Final indicators from systematic literature review where data was available

Exposure			
Percentage of days per year over 25 degrees Celsius^d^	Inland flooding and predicted sea-level rise^e^	Extreme wildfire smoke^f^	Ground-level ozone^d^
**Sensitivity**			
< 5 years old^a^ > 65 years old^a^ Cardiovascular diseases^b^ Respiratory diseases^b^ Renal diseases^b^ Mental health diseases^b^ General health^c^ Multiple chronic conditions^c^	5 years old^a^ > 65 years old^a^ Mental health diseases^b^ Chronic lung diseases^b^ General health^c^ Multiple chronic diseases^c^	5 years old^a^ > 19 years old^a^ Respiratory diseases^b^ Cardiovascular diseases^b^ Chronic health problems^c^	< 19 years old^a^ > 65 years old^a^ Respiratory diseases^b^ Cardiovascular diseases^b^ Diabetes^b^ General health^c^ Multiple chronic conditions^c^
**Adaptive capacity**			
Low education^a^ Poverty* Visible minority status^a^ Indigenous status^a^ Poor housing condition^a^ High population density^a^ Impervious surfaces^d^ Living alone^a^ Heat response plans^g^	Poor housing condition^a^ Poor access to transit^c^ High population density^a^ Weak social network^c^ Living alone^a^ Flood hazard planning and mitigation guidelines^h^ Evacuation and displacement plans^c^	Low education^a^ Low income*^a^ Income inequality^d^ Females ^a^ Visible minority^a^ Indigenous status ^a^ Recent immigrants ^a^ Social support and community belonging^c^	Poverty^d^ Low income*^a^ Unemployed*^a^ Visible minority status^a^ Indigenous status^a^ Immigrant status^a^ Nonurban^a^ Public transit use ^a^ Low fruit and vegetable intake^c^

*Indicators were removed after first-stage PCA was run to eliminate one of two indicators of similar concepts ^a^Statistics Canada, 2016 Census ^b^Chronic Disease Registry Dashboard, British Columbia Center for Disease Control (BCCDC), ^c^My Health, My Community Population Survey, Vancouver Coastal Health, ^d^The Canadian Urban Environmental Research Consortium, ^e^City of Vancouver, Fraser Basin Council ^f^Optimized Statistical Smoke Exposure Model, BCCDC, ^g^Municipal Heat Response Planning in British Columbia, Canada Report, BCCDC, ^h^The Resilient-C Project and School of Population and Public Health, University of British Columbia

After finalising the indicators with stakeholders, appropriate quantitative, geocoded and consistently available data sources for most of the study area were identified. Proportion of days in a year in heat events based on maximum temperature was selected and extracted from high resolution temperature models (2001–2010) [59, 60]; heat events were defined as having three or more consecutive days with maximum daily temperature greater than the 95th percentile of daily normal maximum temperatures (or 25 degrees in the Vancouver area). This heat criteria is conservative given than mortality has been shown to increase in the BC Coastal region even below 25 degrees [61]*.* Components of urban heat island [62] measures such as impervious surfaces and population density were not used for exposure as we included only driving forces or shocks. They were more appropriately placed in the adaptive capacity category given that they are potentially modifiable. Institutional guidelines, such as heat response plans, were derived from an existing review conducted to describe approaches to addressing extreme heat risks by health authorities and municipalities in BC [63].

For flooding, City of Vancouver flooding data and Fraser Basin Council flooding scenario B and D data (sea-level rise of 1 m by year 2100) were used [64]. The flooding models are mostly based on SLR with some inclusion of inland flooding where data were available. Specifically, the flood model from the City of Vancouver includes factors such as overland inundation from the coastal inlet (seasonal high tide, storm surge, and wave runup), extreme winter tide, and the impact of rainstorms on overflow of the sewer network. The Fraser Basin Council model includes sea level rise, river flow rate, and potential dike breach scenarios. Flooding data was not available in some of the Northern rural VCH communities (e.g. Bella Coola, Sunshine Coast). In these and other areas where higher resolution data was not available, a conservative approach was used to classify all areas within 500 m of water bodies (ocean, river, and lake) as ‘exposed.’ Sex was excluded as a relevant factor because there was no clear direction of association between males and females. Urbanicity, defined as the “impact of living in urban areas at a given time” [65], was also excluded because there was no clear direction of association in the literature, despite its frequent mention as a potentially relevant adaptive capacity factor.

For wildfire smoke, proportion of days with average PM_2.5_ concentration > = 25μg/m^3^, among all days (905 days in total) during the five most intense fire seasons in the last 10 years (April 1 to September 30 in year 2009, 2010, 2014, 2015, and 2017) was extracted from an optimized statistical smoke exposure model. Income inequality was operationalized from the material deprivation index, which are factor scores based on Canadian Census variables: average household income, unemployment rate, and high school education rate. Not having social support and community safety were considered if participants responded ‘community belonging (not strong/ not somewhat strong)’ in the MHMC population survey. Recent immigrants were classified as having immigrated less than 5 years ago from the 2016 Census. Municipal guidelines on wildfire smoke were not available for British Columbia.

For ozone, annual modelled concentration estimates from the Canadian Urban Environmental Health Research Consortium (CANUE) were used. Poverty was derived from the social deprivation index, which is based on factor scores of the Canadian Census and incorporates the following variables: proportion of the population separated, divorced, or widowed; proportion of the population that lives alone; and proportion of the population that has moved in the past 5 years. Nonurban was considered if neighbourhoods had less than 400 people per square kilometer. Low fruit and vegetable intake were derived from respondents reporting they had less than 5 servings of fruits and vegetables daily. Both male and female sex were found to be significant modifiers in the literature with similar amounts of supporting evidence, thus neither was included as a final determinant. As with wildfire smoke, municipal guidelines for ground-level ozone were not available.

For all hazards, surrogate indicators from the Chronic Disease Registry Dashboard were used, such as rates of chronic obstructive pulmonary disease, chronic kidney disease, and depression, to operationalize existing chronic conditions. Visible minority group was used instead of race/ethnicity to reflect a more appropriate vulnerable group in the British Columbia context. The latter decisions were made based on expert opinion instead of a data-driven method, so we would not expect agreement with literature or other assessments. Some key data sources were not available among final determinants, such as air conditioning of homes, pregnancy, and outdoor worker numbers. More details of all the data source decisions and indicators can be found in Additional file 3.

### Principal component analysis

The results tables below outline the number of components retained with eigenvalues higher than one, the corresponding variables that loaded highly within each component, and the proportion of variance that each component explains.

### Categorical index PCA scores

#### Extreme heat

Table 2 presents the results of the PCA for the sensitivity and adaptive capacity indices for extreme heat. The table summarises the variables that loaded highly within each component for each category. For sensitivity, three components were retained that met the criteria. The components explained 48.9, 14.4, and 12.5% of the variance respectively. Collectively, the three components explained 75.8% of the total variance of the heat sensitivity index. For adaptive capacity, four components were retained with eigenvalues higher than one. The components explained 41.6, 24.1, 11.1, and 10.4% of the variance respectively. Collectively, the four components explained 87.2% of the total variance of the heat adaptive capacity index across the region.

**Table 2:** Principal Component Analysis Results for Extreme Heat Sensitivity and Adaptive Capacity

Components	Sensitivity Variables	Proportion of variance	Adaptive Capacity Variables	Proportion of variance
**1**	Mental health diseases (anxiety and depression), Respiratory diseases (asthma and chronic obstructive pulmonary disease), Cardiovascular diseases (acute myocardial infarction, and coronary artery bypass graft), Cerebrovascular diseases (hospitalised stroke)	48.9%	Low education, Housing not suitable, Living alone, Indigenous status	41.6%
**2**	Age	14.4%	Population density, Impervious surfaces)	24.1%
**3**	General self-rated health Multiple chronic diseases	12.5%	Total minority	11.1%
**4**			Heat strategy guidelines	10.4%
**Total variance explained:**		75.8%		87.2%

#### Flooding

Table 3 presents the results of the PCA for the sensitivity and adaptive capacity indices for flooding; it summarises the variables that loaded highly within each component for each category. For sensitivity, three components were retained that met the criteria. The components explained 40.9, 21.6, and 17.5% of the variance respectively. Collectively, the three components explained 80.0% of the total variance of the flooding sensitivity index across the region. For adaptive capacity, five components were retained that met our criteria. The components explained 31.4, 22.3, 13.6, 7.9, and 6.7% of the total variance respectively. Collectively, the five components explained 81.9% of the total variance of the flooding adaptive capacity index across the region.

**Table 3:** Principal Component Analysis Results for Flooding Sensitivity and Adaptive Capacity

Components	Sensitivity Variables	Proportion of variance (%)	Adaptive Capacity Variables	Proportion of variance (%)
**1**	Mental health diseases (anxiety and depression), Respiratory diseases (asthma and chronic obstructive pulmonary disease)	40.9	Low education, Housing needing major repair, Unofficial language, Indigenous status, Living alone	31.4
**2**	Age (< 5 and > 65 years old)	21.6	Have a family doctor, Transit stop nearby, Population density	22.3
**3**	General self-rated health Multiple chronic diseases	17.5	Recent immigrants, Visible minority	13.6
**4**			Having emergency supplies, Having 4+ people to confide in	7.9
**5**			Flood strategy guidelines	6.7
**Total variance explained:**		80.0		81.9

#### Wildfire smoke

Table 4 presents the results of the PCA for the sensitivity and adaptive capacity indices for wildfire smoke, which summarises the variables that loaded highly within each component for each category. For sensitivity, three components were retained that met the criteria. The components explained 47.5, 16.9, and 13.3% of the total variance respectively. Collectively, the three components explained 77.7% of the total variance of the wildfire smoke sensitivity index across the region. For adaptive capacity, four components were retained based on the criteria. The components explained 44.1, 23.3, 15.2, and 11.2% of the total variance respectively. Collectively, the four components explained 93.8% of the total variance of the wildfire smoke adaptive capacity index across the region.

**Table 4:** Principal Component Analysis Results for Wildfire Smoke Sensitivity and Adaptive Capacity

Components	Sensitivity Variables	Proportion of variance (%)	Adaptive Capacity Variables	Proportion of variance (%)
**1**	Respiratory diseases (asthma and chronic obstructive pulmonary disease), Cardiovascular diseases (acute myocardial infarction, coronary artery bypass graft, hypertension), cerebrovascular diseases (hospitalised stroke)	47.5	Low education, Female	44.1
**2**	Age (< 5 and > 65 years old)	16.9	Indigenous status, Recent immigrants, Visible minority	23.3
**3**	General self-rated health	13.3	Income inequality	15.2
**4**			Community belonging	11.2
**Total variance explained:**		77.7		93.8

#### Ground-level ozone

Table 5 presents the results of the PCA for the sensitivity and adaptive capacity indices for ground-level ozone, which summarises the variables that loaded highly within each component for each category. For sensitivity, three components were retained that met the criteria. The components explained 40.3, 16.3, and 15.7% of the total variance respectively. Collectively, the three components explained 72.3% of the total variance of the ozone sensitivity index across the region. For adaptive capacity, five components were retained based on the criteria. The components explained 32.4, 20.0, 16.6, 10.5, and 8.6% of the total variance respectively. Collectively, the five components explained 88.1% of the total variance of the ozone adaptive capacity index across the region.

**Table 5:** Principal Component Analysis Results for Ground-level Ozone Sensitivity and Adaptive Capacity

Components	Sensitivity Variables	Proportion of variance (%)	Adaptive Capacity Variables	Proportion of variance (%)
**1**	Chronic lung disease Diabetes Hypertension	40.3	Immigrant status, Visible minority	32.4
**2**	Age (< 5 and > 65 years old)	16.3	Transit commuter, Material deprivation	20.0
**3**	General self-rated health, Multiple chronic conditions	15.7	Non-urban area, Indigenous status	16.6
**4**			Social deprivation	10.5
**5**			Low fruit and vegetable intake	8.6
**Total variance explained:**		72.3		88.1

#### Overall PCA results

Table 6 presents the results of the contributions of the different categories (derived from percentage of variance) to the overall vulnerability index scores for all hazards after incorporating the exposure scores into a PCA along with the sensitivity and adaptive capacity scores. The final PCA results determined the corresponding weights that were used in calculating the final vulnerability index scores. The results show that sensitivity is weighted much higher for extreme heat, wildfire smoke, and ground-level ozone, and adaptive capacity is higher for flooding. Components that explained the least variation include exposure for extreme heat and ground-level ozone, sensitivity for flooding, and adaptive capacity for wildfire smoke. These results seem reasonable given the province’s experiences of widespread smoke in the past wildfire seasons due to increased frequency of hot, dry, and windy conditions, showing that less can be done to control the exposure of wildfire smoke compared to flooding, and to some extent, ground-level ozone and extreme heat. Overall, there are opportunities for *actionable adaptation of capacities* (15.8–48.5%) across all hazards, with the most observed in flooding.

**Table 6:** PCA Results for All Overall Vulnerability Indices

	Proportion of variance explained for each index (%)			
	Extreme Heat	Flooding	Wildfire Smoke	Ground-level Ozone
Exposure	11.1	32.2	30.0	20.5
Sensitivity	**60.9**	19.3	**54.2**	**50.6**
Adaptive Capacity	28.0	**48.5**	15.8	28.9

### Geospatial analysis

#### Category-specific index maps

**Fig. 4:**
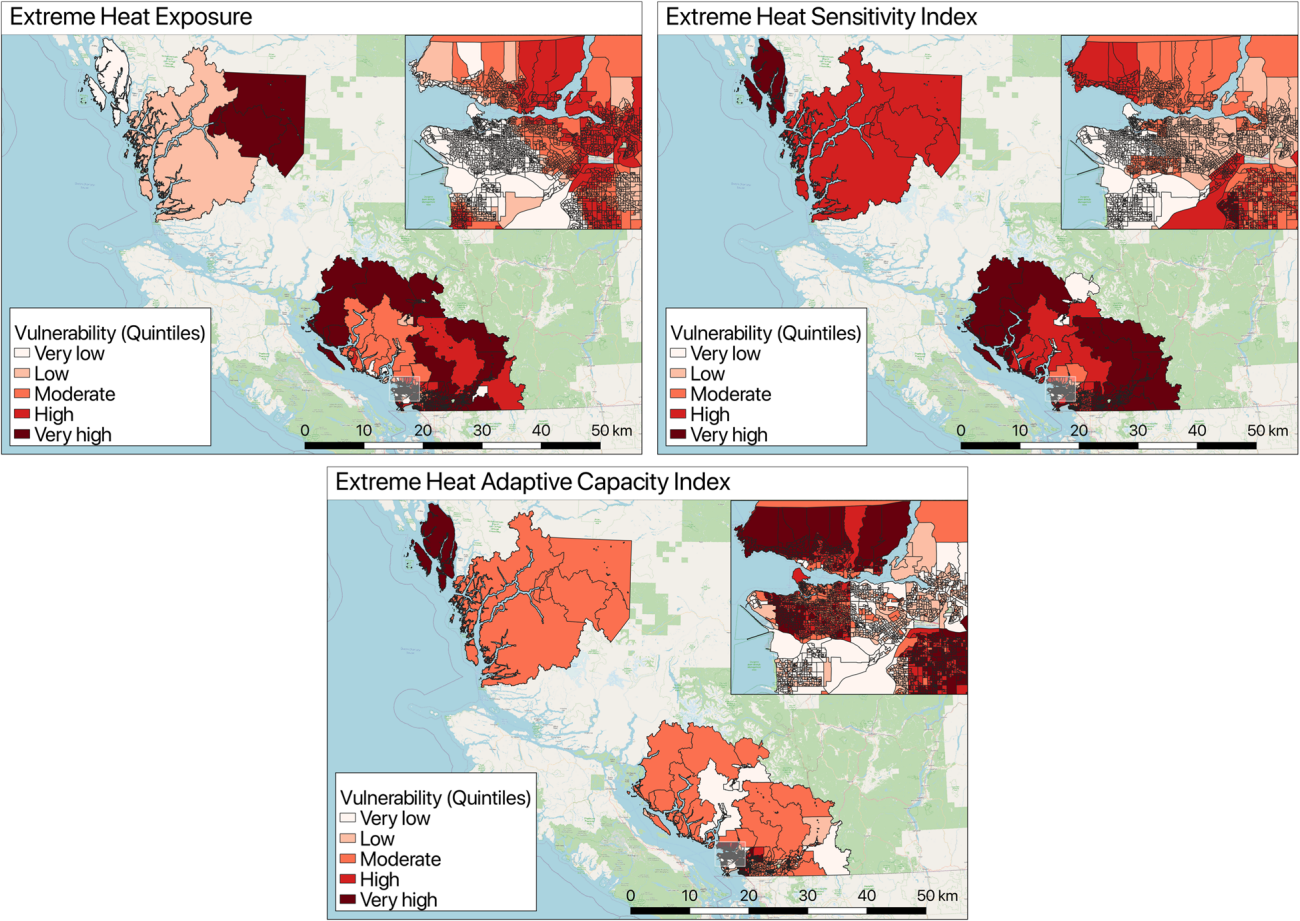
Extreme heat exposure, sensitivity, and adaptive capacity index maps

The final category-specific and overall index scores were mapped for all hazards, which shows the variability in exposure, sensitivity, and adaptive capacity within the study region. Figure 4 present the results from one hazard example results - the extreme heat exposure and the corresponding sensitivity and adaptive capacity indices for the entire study area. The remaining maps for flooding, wildfire smoke, and ground-level ozone can be found in Additional file 5.

#### Overall vulnerability index maps

**Fig. 5:**
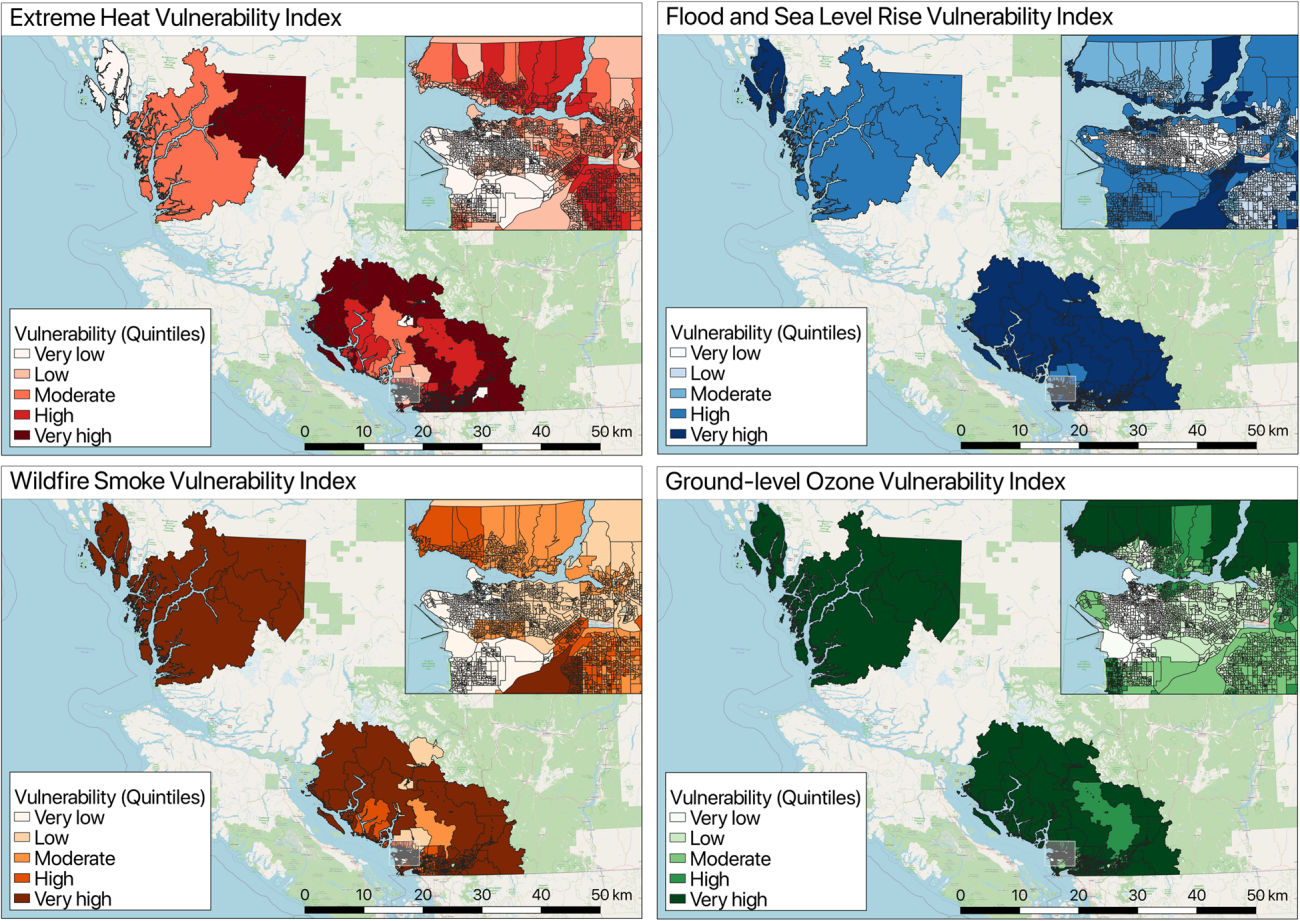
Overall vulnerability index maps for all hazards

Overall vulnerability index scores from the PCA results were also mapped for all hazards. Figure 5 presents the overall vulnerability index maps for all hazards. Reflecting the PCA results, the observed spatial patterns can be drawn from the PCA results. For example, extreme heat is ~ 11% driven by the exposure indicator (% of days above 25 degrees Celsius), ~ 28% by the adaptive capacity variables (e.g. education, visible minority status, population density, impervious surfaces, heat guidelines etc.), and ~ 61% by the sensitivity variables (age, pre-existing conditions).

#### Maps of highest overall vulnerability quintiles across all hazards

**Fig. 6:**
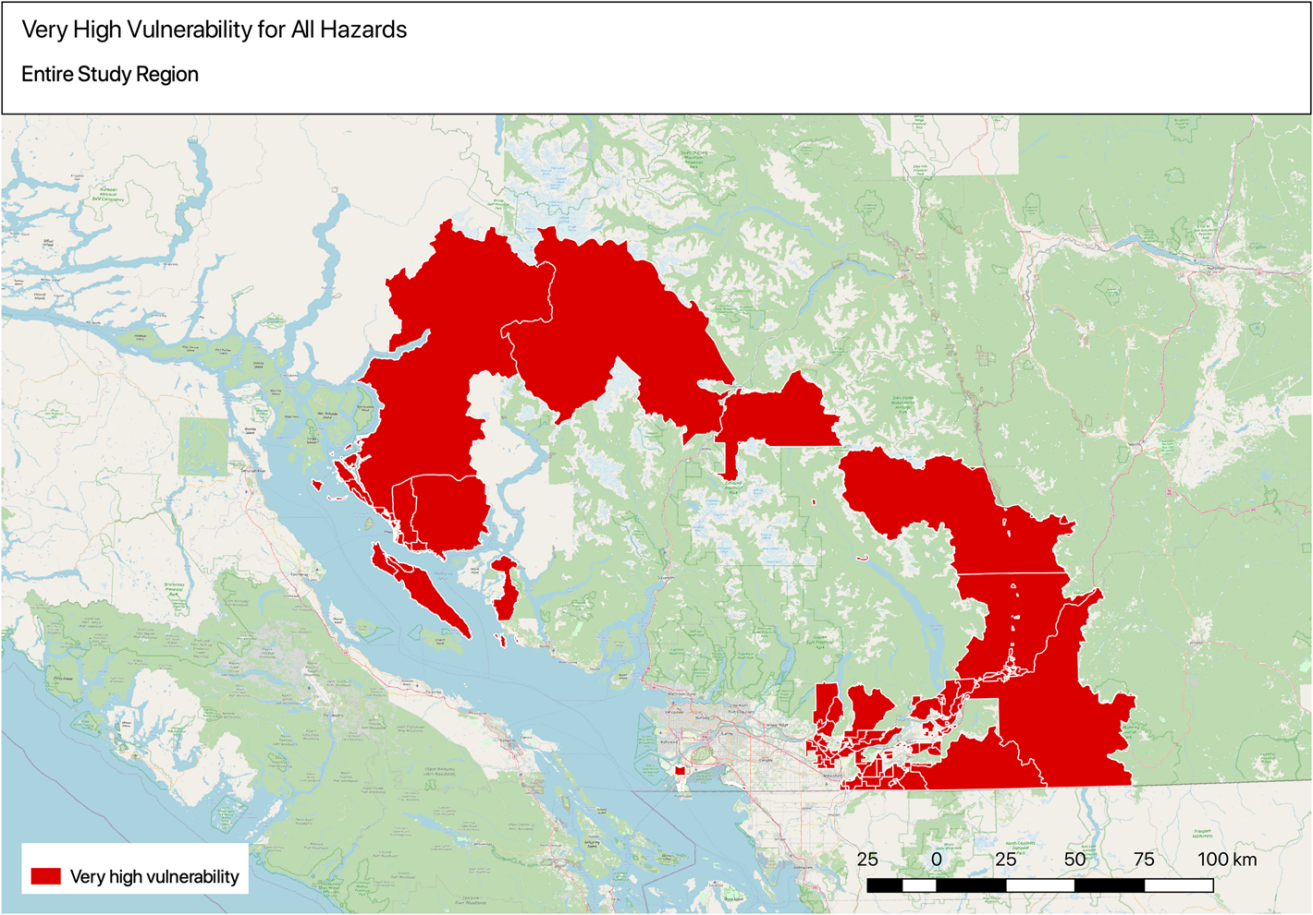
Identifying very high vulnerability areas for all hazards

Two hundred one DAs were identified within the entire study area that were in the highest overall vulnerability index quintiles of all hazards, with most of the concentration of highest vulnerable DAs observed in eastern areas of Metro Vancouver. Spatial heterogeneity can be observed for the entire study area and within Metro Vancouver (Fig. 6).

#### Maps of lowest overall vulnerability quintiles across all hazards

**Fig. 7:**
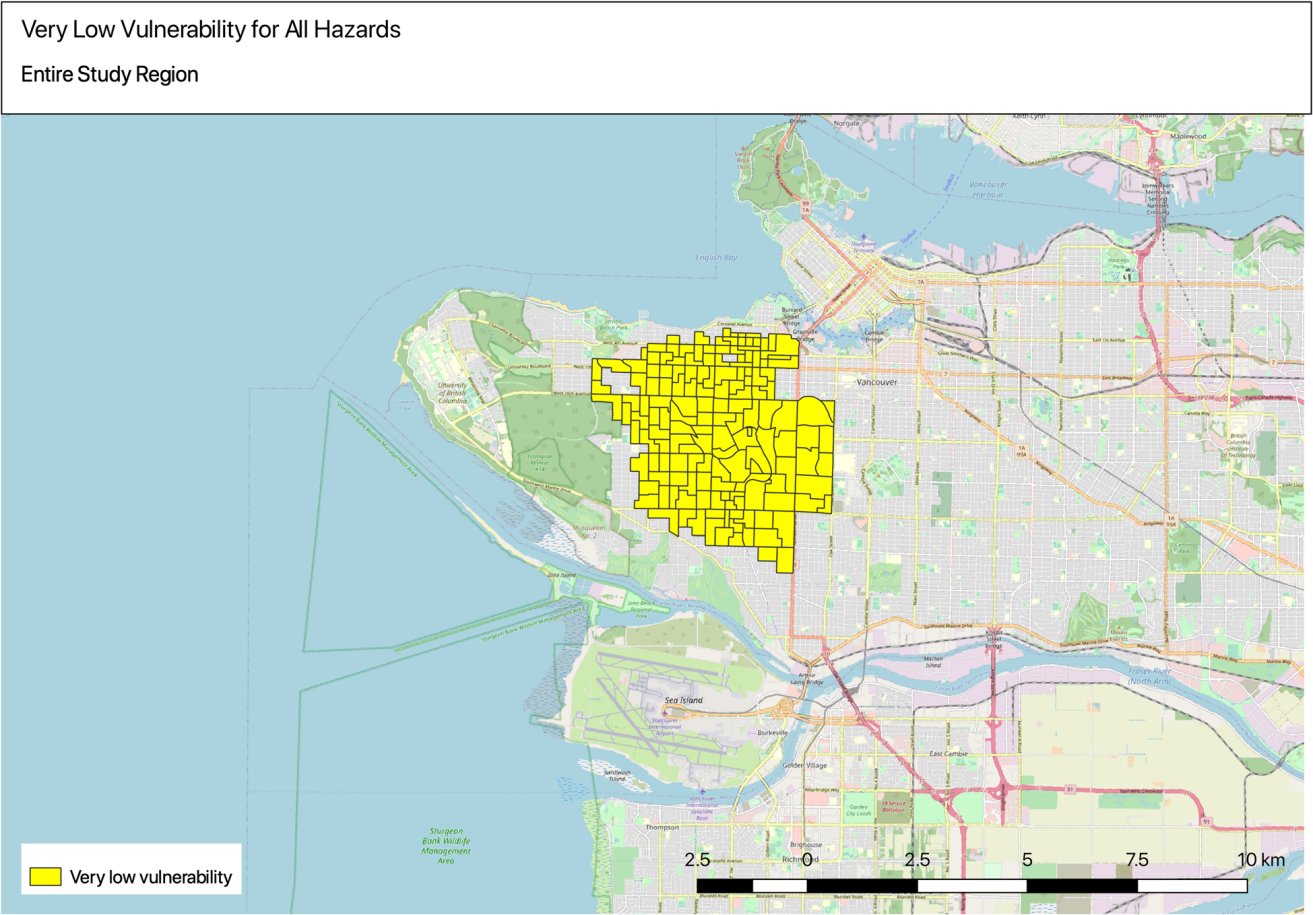
Identifying very low vulnerability areas for all hazards

One hundred fifty-three DAs were identified within the entire study area that were in the lowest overall vulnerability index quintiles of all hazards. Spatial patterns were observed for the entire study area, which showed that the lowest vulnerability DAs of all hazards were mostly concentrated within the west side of Vancouver (Fig. 7).

Overall, when comparing across all hazards, the highest vulnerability DAs are observed in the eastern part of the study region, which has important implications for future targeted investments to increase adaptive capacity of these communities. Spatial patterns were also more evident among the least vulnerable DAs across all hazards, which may also reflect broad historical socio-economic differences across the region. Some of the lowest vulnerability areas were concentrated within the west side of Vancouver, where the DAs have some of the highest levels of health status and adaptive capacity (e.g. income, green space, access to transit, housing). The results show the need for targeted adaptation planning with regional coordination to reduce inequity of health impacts by targeting more exposed, sensitive, and less adaptive communities across the region. The next section outlines some potential ways to approach this.

## Discussion

The combination of population growth, urbanisation and acute shocks related to changing weather and climate patterns are creating new climate realities in communities around the world. A recent review of vulnerability indices [20] concluded that most indices have not adequately justified their methodological decisions in terms of weighting and selecting component indicators. Our climate vulnerability assessment attempts to address many of these concerns by incorporating a systematic review to identify priority risk factors for each of four pre-specified specific climate hazards, conducting stakeholder consultation to collect appropriate data sources, and by using principal component analysis to objectively select and weight both parent (overall vulnerability) and daughter (adaptive capacity, sensitivity) index scores. Altogether, we reviewed 281 epidemiological papers and collected data for 36 determinant indicators across all four hazards. For each adaptive capacity and sensitivity index score, 3–5 principal components explaining 72–94% of the total variance were retained based upon 7–13 indicators. There were varied opportunities to adapt capacities across all hazards (16–49%), with the greatest opportunity found for flooding (49%). Mapping the index scores showed that there was significant spatial heterogeneity throughout the entire study region, indicating that there were wide disparities in exposures, sensitivities, and adaptive capacities within and between neighbourhoods.

In the findings of the systematic literature review, a table of determinants (Table 1) was identified in our review similar to other reviews of heat [50, 53, 54], flooding [45–48], wildfire [52, 66], and ozone [57, 67]. There was generally less information on wildfire and smoke compared to the other hazards, however, all of the indicators identified in this review for wildfire smoke have been identified in other reviews. To operationalise these determinants, some indicators were specifically selected because they were considered more appropriate in the British Columbian context than those specifically indicated in the literature, which was dominated by studies from the USA (e.g. visible minority instead of African American / Hispanic). Furthermore, efforts were made to categorise the determinants into major categories (exposure, sensitivity, and adaptive capacity) and sub-categories (age, sex, race/ethnicity, socioeconomic, climate, existing health conditions, built environment, and institutional setting) to allow for comparisons. However, some sub-categories did not have determinants in all hazards after finalising the list through the data reduction process (e.g. no social cohesion and institutional setting measures were identified for ozone). The major categories were found to be more consistently available and actionable. Overall, we recommend this categorisation (exposure, sensitivity, and adaptive capacity) along with a combination of objective assessment and expert judgment to contextualise the local indicators and data needs.

The results of our PCA for overall vulnerability show that sensitivity scores explained more of the variance of the overall vulnerability of extreme heat (60.9%), wildfire smoke (54.2%) and ground-level ozone (50.6%), while adaptive capacity scores did the same for flooding (48.5%). Based on this, local response planning may emphasize interventions that will reduce the variability of sensitivity and exposure depending on the hazard of interest. For example, as exposure may be more feasible to modify for flooding compared to extreme heat, planners can focus more attention to communities living near the coast and along floodplains by building, maintaining, or enhancing dykes and ensuring the buildings within these communities meet minimum flood construction levels. With some hazard exposures (e.g. flooding) being more modifiable than others (e.g. wildfire smoke), planners can weigh the importance of these exposure indicators accordingly in their vulnerability assessments. For sensitivity variables, pre-existing health conditions (e.g. mental health and respiratory diseases) dominated the components that explained the most variance among all hazards. Attention can thus be given to plan emergency response and public health resources according to the locations of these communities that are more sensitive to these climate change-related health issues. It is well known that increased distance to the nearest hospital may increase vulnerability by preventing immediate relief and prolonging recovery [68], although less is known about the recommended physical range to optimise planning, and data on public health facilities was not available to incorporate this indicator. Overall, as the senior population continues to grow to outnumber youth in North America [69] and climate events such as the wildfire smoke seasons increase in frequency and severity in areas like western North America, governments should prepare for the rise in sensitive communities to these hazards. For example, efforts have been underway in BC to build resilience across the VCH authority by projecting the impacts of climate change on health facilities by 2020 and 2050 [70]; some local professionals in this region acknowledge that the design of the facilities today will impact patient care currently and in the future.

From a pragmatic perspective, another question bears discussion: *do adaptive capacity index scores and maps provide more information that is more actionable than exposure, sensitivity or ‘overall vulnerability’ maps from a local urban or health planners’ perspectives?* That is, municipal governments may not be able to (rapidly) mitigate the climate change exposures or change the demographics and health profiles of their local population, but are capable of affecting city and neighbourhood-level policies, programs, and resources that impact the adaptive capacities of these communities, such as housing, green space, immigration programs, and building climate-resilient facilities. In previous studies, indicators with *adaptive capacity-like* variables were divided into two sub-categories: socio-economic and built environment vulnerability [68]. The socio-economic vulnerability index and built environment vulnerability index were created in Norway to derive two of the first social vulnerability indices [16, 68]. In our study, we grouped these sub-categories into the overarching category of adaptive capacity for two reasons: 1) to operationalise the components of vulnerability according to the Intergovernmental Panel on Climate Change Fourth Assessment [9] and WHO Framework for Health System Resilience [1]; and 2) to help planners answer the equity-related question, *of all determinants found to be epidemiologically important for the adaptive capacity of these communities, which one would reduce variability in vulnerability the most?* For example, in the findings of the extreme heat adaptive capacity index, unsuitable housing, low education, living alone, and Indigenous communities were four variables included in the component that explained the most variance across the study area. A planner concerned with inequitable impacts of extreme heat may consider giving more attention to policies and programs that address these indicators, such as retrofitting homes for better ventilation and cooling, providing material resources and building capacity in low SES neighbourhoods, and identifying households in high smoke exposed areas where there are Indigenous communities or populations living alone.

Similarities can also be identified across the adaptive capacity index scores of the other hazards – flooding, wildfire smoke, and ozone. Our results showed that low SES (e.g. low education, language, or housing needing major repair) and households with visible minorities, Indigenous communities, and females were variables included in the components that explained the most variability in vulnerability across all these hazards (31.4, 44.0, and 37.2%, respectively). Appropriate adaptation interventions may therefore be targeted towards women, Indigenous populations, or visible minorities in communities where there is high exposure and low adaptive capacity.

Visible minority status was chosen based on expert opinion as a more appropriate measure in the BC context (as opposed to African and Latin American groups found to be at risk in US-based studies); future studies should consider researching and understanding which specific groups are impacted the most in BC or other local contexts to better understand and invest in tailored interventions. Other notable variables that were prominent for these hazards include population density, being a transit commuter, and having access to a family doctor. Addressing issues of access to healthcare, transportation, and other essential amenities in areas with increasing population density during a climate event should similarly be on the agenda, and similarly for populations who rely on transit and/or live in rural communities.

Finally, another potential utility of these maps can be developed by assessing categorical index maps to identify areas of high exposure and very high and low adaptive capacities and sensitivities. This can be done by identifying thresholds of the highest and lowest quintiles of categories or vulnerabilities (20th and 80th percentiles). Using extreme heat as an example, Fig. 8 in Additional file 6 shows that 608 DAs are of high priority to professionals because such areas have very high exposure and very high sensitivity. Potential interventions include increasing and protecting green canopy and public spaces, retrofitting homes for better natural cooling, and mapping out and planning for better access to public cooling centers in these communities. Similarly, we may also identify areas with high flood exposures that were protective from having households with high adaptive capacities – these 203 DAs present opportunities to explore and learn about what these communities are doing differently (see Fig. 9 in Additional file 6). From an equity standpoint, there may be a higher likelihood of reducing vulnerability in these particular areas since the adaptive capacities are arguably more amenable to changes. By identifying quintile thresholds with wildfire smoke as an example, we can also assess areas of the highest or lowest quintiles of vulnerability. Fig. 10 in Additional file 6 shows that 72 DAs have high wildfire smoke exposure and sensitivity and low adaptive capacity; sensitivity had the highest range for component weight percentage (19.3–60.9%) and thus the relevant programs and tools outlined earlier for sensitivity could be targeted in these areas from a planner’s perspective. Fig. 11 in Additional file 6 shows that 21 DAs have very low ozone exposure and sensitivity and very high adaptive capacity and reflected similar patterns of the overall low vulnerability maps whereby spatial patterns are observed in the west side of Vancouver. Essentially, by having a combination of categorical and overall vulnerability maps, potential users of these tools have the opportunity to not only assess areas for service planning, but also raise and test new hypotheses for future research.

### Strengths and limitations

As with most ecological analysis, there is potential for modifiable area unit problems, whereby aggregated exposures and outcome statistics will vary with changes to area boundaries (e.g. from dissemination areas to municipalities). As the analysis is based on relative differences between all DAs within the study region, the factors that explain the variance as being the most significant may change if the study area was expanded to the rest of the province or restricted to a smaller area (e.g. only Metro Vancouver). There are also limitations related to missing census and household survey data for certain neighbourhoods due to low data counts. As such, there may be exposure misclassification from local ascertainment of health and sociodemographic data (e.g. using average rates or rates from nearby neighbourhoods where data were not available). Furthermore, this study was limited in its flood exposure data, especially in the northern communities within Vancouver Coastal Health (e.g. Bella Coola, Sunshine Coast). Inland flooding data, for example sewage or drainage issues after a large rainfall event, was inconsistently available across the entire study with the use of two different flood models (City of Vancouver and Fraser Basin Council). Nevertheless, we were able to use high resolution (within 1 cm elevation) sea-level rise and flood models where available in the BC context. Where data were missing, a conservative approach was employed, whereby all areas within 500 m of water bodies was classified as ‘exposed’ to ensure the vulnerability of areas known to have seasonal flooding would not be underestimated. Elevation was not incorporated in the conservative approach given the lack of high-resolution data available outside of Metro Vancouver. More climate projections for other exposures (extreme heat, wildfire smoke, and ozone) should be included in the future as they become available and inclusion of additional exposures may also be warranted in future updates.

Certain determinants for extreme heat, ground-level ozone, and flooding that were not identified in this review and have been included in other international reviews were not selected based on our data-driven tabular approach, in that not as many papers reported an epidemiological relationship. These other indicators from the wider literature include:

1. Extreme heat: having pre-existing diabetes or digestive disorders [50, 71, 72], teenagers and adolescents (under 18 years old) [51, 72–74], PM_10_, O_3_ [50, 71, 75], latitude [50], medication usage (e.g. psychoactive, anticoagulants, nitrates, diuretics) [76–78], pregnancy [51, 79, 80], urbanicity [81–83], being confined to bed [54], not leaving home daily [54], being unable to care for oneself [84], patients living outside of retirement homes [54], taking extra showers during hot days [54], wind speed and direction [85], dew point [86], vapor pressure [87], cloud cover [88], public transit use [89], and ecoregion [90].
2. Flooding: pregnancy [46], having pre-existing cardiovascular and gastrointestinal conditions [46, 91], malnutrition [46], number of severe storm and hurricane events experienced [46–48, 91], flood duration [46, 47], having below ground living space [55], rental status [55], and certain occupational types (outdoor workers).
3. Ozone: female [49], ethnic minorities [49], lower educational level [49], and low Vitamin E and C diets [58].

Similar to previous studies [25], this analysis was unable to incorporate data on genetics, and less quantifiable variables such as awareness levels of the population, number of outreach programs and proximity of communities to health care facilities. Although our study did not include these or some of the identified indicators after finalising the systematic review, future studies should consider these variables as stronger evidence emerges and more data become available to justify their inclusions. Similarly, this systematic review did not include the analysis of grey literature, review papers, and studies outside of North America due to feasibility and the study’s regional scope. Key indicators may have been missed in the data tabulation approach that otherwise would have been included if we included these studies. Future studies can include such data sources in the literature review stage to identify missing indicators and may consider emphasizing literature sources most representative of their region, or expanding to the global literature if deemed relevant.

Unlike previous studies, this study was able to take advantage of other data that were available in the British Columbian context and at the community level, such as various chronic health conditions like diabetes (derived from the local health authority level), the presence/absence of municipal preparation guidelines for extreme heat and inland flooding, social cohesion indicators such as the perceived strength of a household’s social network, having household emergency supplies as a surrogate for having an evacuation and displacement plan, having transit options nearby (less than 5 min walk), and indices of the level of community material and social deprivation. Future climate vulnerability assessments can also consider the incorporation of these indicators in their data collection and analyses and for other climate-related hazards that may be more relevant in other contexts.

Caution should be taken with the variable selection and interpretability of the vulnerability indices. This is due to general concerns with creating indices using proxies and variable selection without empirical evidence. To mitigate these concerns, systematic literature review and PCA were used to ensure objectivity and weighting of the different components, as opposed to choosing arbitrary weights or assuming equal weights, and sensitivity analyses have been done with different variables to test the robustness of the index. Nevertheless, initial and ongoing involvement with the advisory team of our partners in the provincial health authorities helped ensure relevance of these indices to the contextual concerns in British Columbia. The flexibility of this methodology allows our partners to gather more feedback on the included variables from local stakeholders and make modifications to the interactive map products.

PCA has been contested compared to factor analysis, which has been suggested that the former falls short by not discriminating between shared and unique variance, and thus inflated estimates of variance can be produced [92]. However, the overall goal of using PCA in this study was to not only simplify numerous risk factors of vulnerability into index scores, but also to explore the potential determinants and interventions that can address the inequitable health impacts from climate change. That is, the absolute index scores are not as important as it is to understand how different communities’ vulnerabilities compare with one another or with other vulnerability measures. In other words, the systematic literature review was used to identify variables that have shown strong epidemiological associations, and PCA helps identify which indicators to target from an equity lens. This can suggest, in part, where public health and urban planning may most effectively intervene to reduce health and spatial equity within the regions of interest.

The idea of measuring vulnerability has also been contested [21, 24] due to the difficulty of operationalising a complex process of adaptation – ‘a change in processes, practices, and structures to moderate potential damages or to benefit from opportunities associated with climate change’. That is, adaptive capacity is a theoretical measure of the future ability to reduce future risks through individual- and societal-level interventions, and other frameworks have adopted more risk-management framing. In these framings, the IPCC Special Report on Extreme (2012) [93] and the IPCC AR5 Synthesis Report (2014) [2] for examples, details the histories of how adaptation and disaster risk management (DRM) stem from different research fields. The adaptation field has focused on understanding and designing measures for disaster preparedness and the DRM field on how to adapt to actual or expected climate and its effects. In practice, the IPCC acknowledges that these approaches are likely overlapping and can be pursued simultaneously. For example, the WHO Operational Framework that was used for this study is based on the ‘resilience’ approach developed by UKAID department. This approach aims to guide professionals working in health systems and health determining sectors to understand and prepare for the additional climate change-related health risks [1]. Our stakeholders in the local health authorities felt it was appropriate to implement this framework given their goals. Framing in resilience has also been contested in that the current conceptualisation has not considered essential services, how access is impaired by hazards, nor how access may be spatially explicit or operational [32]. Our study, with our adaptive capacity index, attempts to provide more actional, spatially-explicit information on some of these essential services that may be impacted during a hazard in our adaptive capacity index. Future studies may consider different frameworks that are best suited for the local planning and risk management context.

Although not tested against observational data, the vulnerability indices in this study were developed for future planning and helps establish a baseline of current conditions which can then be used to forecast where and when hazard exposures and sensitivities may change, and provide insights for targeting investments to increase adaptive capacities at the local level. Previous evaluation tests of vulnerability indicators have used regression analyses to assess the change in relative risk of adverse climate-related health outcomes [94, 95], for example an independent samples test to assess the difference of mean mortality and ambulance callout with and without high heat exposure and vulnerability [95], and Poisson regression to model the interaction of vulnerability indicators and hospitality and mortality counts in five different states as a part of the Environmental Public Health Tracking network [96]. Future studies can use the above methods to test the vulnerability indicators developed in this study or for other cities using similar methodology.

Overall, the study benefits from a large data-driven approach to yield measures at the community level. Using objective assessments (e.g. systematic literature review, determinant tabulations, principle component analysis, and geospatial analysis) and expert judgment and consultations, we were able to produce index scores for 4 different hazards in 4 different categories (exposure, sensitivity, adaptive capacity, and overall vulnerability). This ultimately allowed the comparisons of 16 index scores across the entire study region to test and generate new hypotheses. As most of the data used in this study are publicly available, this methodology can be replicated in other cities and regions where similar data sources are available or as new data becomes available.

## Conclusion

In addition to assessing overall vulnerability of each hazard, category-specific indices were created to identify populations more sensitive and/or less adaptive to these health hazards. To our knowledge, the creation of category-specific indicators, such as the adaptive capacity index, has no precedent and such indicators may be useful for planners. The key to our methodology and process was engaging with stakeholders to ensure the pertinence of the data used to address specific questions and problems in the BC context. Future studies should be locally construed using systematic literature search criteria specific to the hazards and regions of interest. Our goal was to be transparent with our methodology to allow other groups to replicate and build on this study in other communities and local contexts. Not only was this helpful for our stakeholders to assess and collect data available for this and ongoing vulnerability assessments, but it was also a thoughtful exercise to assess the data missing and needed for future surveillance and monitoring.

Our hope is that the results of this study will help build the public health narrative about climate change-related health impacts, and to promote more capacity building and adaptation activities in British Columbia communities and beyond. By operationalising and quantifying vulnerability risk factors related to climate change shocks and stressors, health planners and policy makers can improve efficiency in the health system and advance urban health, equity, and social justice. These are important components of local and global health guidelines, including Sustainable Development Goals 3, 10, and 11. Specifically, the findings of this study suggest that public health and urban planners would benefit from a regional coordination of investment into adaptive capacity and a framework that enables municipalities to learn from each other. Although different management and governance contexts may approach risk assessment and adaptation differently, application of this methodology can initiate conversations about how to balance decisions based on not just ease of action, but also the evidence on relative contributions from specific determinants to spatial variability in vulnerability. These baseline data could also be used to project future health vulnerability and/or paired with health impact studies which could incorporate information on the absolute risk of each hazard derived from the probability of exposure at a specific magnitude, the relative risk of such exposures for specific health outcomes and the population level rates of those outcomes. In contrast to our analysis which identifies the drivers of variability in vulnerability to each hazard, such impact analyses would identify specific at-risk communities that are projected to have the highest health impacts. Ultimately, we hope that by understanding the locations of vulnerability and actionable areas of adaptation, we can not only moderate the harms of current or expected climate hazards, but also consider these as opportunities for communities and households to become more resilient, healthy, and equitable.

## Supplementary Information

**Additional file 1.**

**Additional file 2.**

**Additional file 3.**

**Additional file 4.**

**Additional file 5.**

**Additional file 6.**

## Abbreviations

ADA: Aggregate Dissemination Area
BC: British Columbia
CANUE: Canadian Urban Environmental Health Research Consortium
CCRHHs: Climate change-related health hazards
CCREs: Climate change-related events
DA: Dissemination Area
FH: Fraser Health
MHMC: My Health, My Community
OECD: Organisation for Economic Co-operation and Development
PCA: Principal component analysis
WHO: World Health Organisation
VCH: Vancouver Coastal Health
