# Supplementary material for: Geospatial indicators of exposure, sensitivity, and adaptive capacity to assess neighbourhood variation in vulnerability to climate change-related health hazards
Source: Environ Health. 2021 Mar 22;20:31. doi: 10.1186/s12940-021-00708-z (PMC7986027; doi:10.1186/s12940-021-00708-z)
Supplement: Supplementary file 1 — Additional file 1. [file 12940_2021_708_MOESM1_ESM.docx]

**Additional file 1 – Systematic literature review criteria and flow charts**

Key search terms were entered into two key databases: PubMed/Medline and Web of Science. Based on the original search strategy, all abstracts were read. Articles were excluded if they did not meet the primary inclusion: epidemiological studies, published in the last 10 years, conducted in North America, published in the English language, and where an explicit link was studied between climate hazards (flooding, extreme heat, wildfire and smoke, and ozone) and health impacts (see full list below). Epidemiological papers were reviewed in full and excluded if they were not relevant after full review, did not meet quality standards according to expert judgment, or if they did not sufficiently report items required in observational studies as outlined in the STROBE checklist (98).

**Extreme heat systematic review:**

**Figure 2a** summarises the results of the systematic review for extreme heat. In total, the search from the two databases returned 2264 papers using the following search terms:

| Hot Temperature/adverse effects  AND  Health/epidemiology [Mesh] OR Morbidity [Mesh] OR hospital* [ALL FIELD] OR emergency* [ALL FIELD] OR Mortality [Mesh] |
| --- |

After removing duplicates and studies that did not meet the primary inclusion, 983 abstracts were reviewed. Studies were further removed if papers were on unrelated subjects, such as biochemistry and molecular biology, and if papers focused on ozone instead of temperature itself. 102 epidemiological papers were reviewed in the final inclusion.


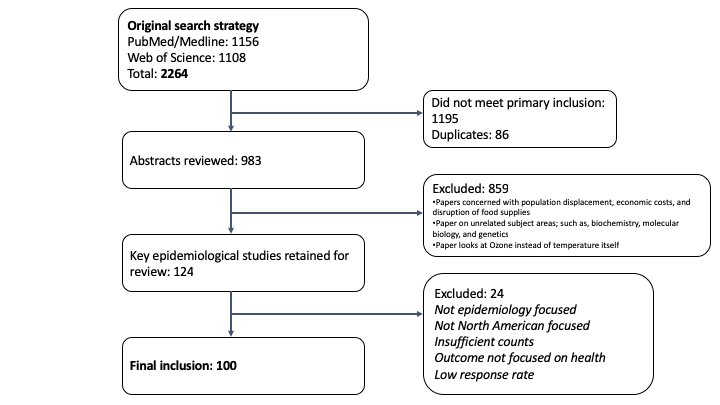
Figure 2a – Flow chart of results from the systematic review of extreme heat

List of final papers for extreme heat:

1. Anderson, G Brooke B. ML. Weather-Related Mortality: How Heat, Cold, and Heat Waves Affect Mortality in the Unite States. Epidemiology. 2009;20(2):205–13.

2. Anderson GB, Dominici F, Wang Y, McCormack MC, Bell ML, Peng RD. Heat-related emergency hospitalizations for respiratory diseases in the medicare population. Am J Respir Crit Care Med. 2013;187(10):1098–103.

3. Auger N, Fraser WD, Arbour L, Bilodeau-Bertrand M, Kosatsky T. Elevated ambient temperatures and risk of neural tube defects. Occup Environ Med. 2017;74(5):315–20.

4. Auger N, Fraser WD, Smargiassi A, Bilodeau-Bertrand M, Kosatsky T. Elevated outdoor temperatures and risk of stillbirth. Int J Epidemiol. 2017;46(1):200–8.

5. Auger N, Fraser WD, Smargiassi A, Kosatsky T. Ambient Heat and Sudden Infant Death : A Case-Crossover Study Spanning 30 Years in Montreal, Canada. Environ Health Perspect. 2015;712(7):712–6.

6. Auger N, Naimi AI, Smargiassi A, Lo E, Kosatsky T. Extreme heat and risk of early delivery among preterm and term pregnancies. Epidemiology. 2014;25(3):344–50.

7. Auger N, Rhéaume MA, Bilodeau-Bertrand M, Tang T, Kosatsky T. Climate and the eye: Case-crossover analysis of retinal detachment after exposure to ambient heat. Environ Res [Internet]. 2017;157(February):103–9. Available from: http://dx.doi.org/10.1016/j.envres.2017.05.017

8. Auger N, Siemiatycki J, Bilodeau-Bertrand M, Healy-Profitós J, Kosatsky T. Ambient Temperature and Risk of Preeclampsia: Biased Association? Paediatr Perinat Epidemiol. 2017;31(4):267–71.

9. Bassil KL, Cole DC, Moineddin R, Craig AM, Wendy Lou WY, Schwartz B, et al. Temporal and spatial variation of heat-related illness using 911 medical dispatch data. Environ Res [Internet]. 2009;109(5):600–6. Available from: http://dx.doi.org/10.1016/j.envres.2009.03.011

10. Bassil KL, Cole DC, Moineddin R, Lou W, Craig AM, Schwartz B, et al. The relationship between temperature and ambulance response calls for heat-related illness in Toronto, Ontario, 2005. J Epidemiol Community Health. 2011;65(9):829–31.

11. Basu R, Feng WY, Ostro BD. Characterizing temperature and mortality in nine California counties. Epidemiology. 2008;19(1):138–45.

12. Basu R, Gavin L, Pearson D, Ebisu K, Malig B. Examining the Association between Apparent Temperature and Mental Health-Related Emergency Room Visits in California. Am J Epidemiol. 2018;187(4):726–35.

13. Basu R, Malig B, Ostro B. High ambient temperature and the risk of preterm delivery. Am J Epidemiol. 2010;172(10):1108–17.

14. Basu R, Ostro BD. A multicounty analysis identifying the populations vulnerable to mortality associated with high ambient temperature in California. Am J Epidemiol. 2008;168(6):632–7.

15. Basu R, Pearson D, Malig B, Broadwin R, Green R. The Effect of High Ambient Temperature on Emergency Room Visits. Epidemiology. 2012;23(6).

16. Basu R, Pearson D, Sie L, Broadwin R. A Case-Crossover Study of Temperature and Infant Mortality in California. Paediatr Perinat Epidemiol. 2015;29(5):407–15.

17. Bayentin L, El Adlouni S, Ouarda TBMJ, Gosselin P, Doyon B, Chebana F. Spatial variability of climate effects on ischemic heart disease hospitalization rates for the period 1989-2006 in Quebec, Canada. Int J Health Geogr. 2010;9:9–11.

18. Bélanger D, Abdous B, Valois P, Gosselin P, Sidi EAL. A multilevel analysis to explain self-reported adverse health effects and adaptation to urban heat: A cross-sectional survey in the deprived areas of 9 Canadian cities Environmental health. BMC Public Health [Internet]. 2016;16(1):1–11. Available from: http://dx.doi.org/10.1186/s12889-016-2749-y

19. Bélanger D, Gosselin P, Valois P, Abdous B. Neighbourhood and dwelling characteristics associated with the self-reported adverse health effects of heat in most deprived urban areas: A cross-sectional study in 9 cities. Heal Place [Internet]. 2015;32:8–18. Available from: http://dx.doi.org/10.1016/j.healthplace.2014.12.014

20. Bélanger D, Gosselin P, Valois P, Abdous B. Perceived adverse health effects of heat and their determinants in deprived neighbourhoods: A cross-sectional survey of nine cities in Canada. Int J Environ Res Public Health. 2014;11(11):11028–53.

21. Berko J, Ingram DD, Saha S. Deaths Attributed to Heat, Cold, and Other Weather Events in the United States, 2006–2010. Natl Health Stat Report [Internet]. 2014;(76):2006–10. Available from: https://www.cdc.gov/nchs/data/nhsr/nhsr076.pdf

22. Bishop-Williams KE, Berke O, Pearl DL, Kelton DF. A spatial analysis of heat stress related emergency room visits in rural Southern Ontario during heat waves. BMC Emerg Med [Internet]. 2015;15(1):1–9. Available from: http://dx.doi.org/10.1186/s12873-015-0043-4

23. Bobb JF, Obermeyer Z, Wang Y, Dominici F. Cause-Specific Risk of Hospital Admission Related to Extreme Heat in Older Adults. JAMA-JOURNAL Am Med Assoc. 2014;312(24):2659–2667.

24. Bobb JF, Peng RD, Bell ML, Dominici F. Heat-related mortality and adaptation to heat in the United States. Environ Health Perspect. 2014;122(8):811–6.

25. Bonhert ASB, Prescott M, Vlahov D, Tardiff K, Galea S. Ambient temperature and risk of death from accidental drug overdose in New York City, 1990-2006. Addiction. 2012;1(3):233–45.

26. Brooke Anderson G, Bell ML. Heat waves in the United States: Mortality risk during heat waves and effect modification by heat wave characteristics in 43 U.S. communities. Environ Health Perspect. 2011;119(2):210–8.

27. Bustinza R, Label G, Gosselin P, Belanger D, Chebana F. Health impacts of the July 2010 heat wave in. BMC Public Health. 2013;(July 2010).

28. Calkins MM, Isaksen TB, Stubbs BA, Yost MG, Fenske RA. Impacts of extreme heat on emergency medical service calls in King County, Washington, 2007-2012: Relative risk and time series analyses of basic and advanced life support. Environ Heal A Glob Access Sci Source. 2016;15(1):1–13.

29. Chen T, Sarnat SE, Grundstein AJ, Winquist A, Chang HH. Time-series analysis of heat waves and emergency department visits in Atlanta, 1993 to 2012. Environ Health Perspect. 2017;125(5):1–9.

30. Cil G, Cameron TA. Potential Climate Change Health Risks from Increases in Heat Waves: Abnormal Birth Outcomes and Adverse Maternal Health Conditions. Risk Anal. 2017;37(11):2066–79.

31. Davis RE, Hondula DM, Patel AP. Temperature observation time and type influence estimates of heat-related mortality in seven U.S. cities. Environ Health Perspect. 2016;124(6):795–804.

32. DeVine AC, Vu PT, Yost MG, Seto EYW, Busch Isaksen TM. A geographical analysis of emergency medical service calls and extreme heat in King county, WA, USA (2007-2012). Int J Environ Res Public Health. 2017;14(8).

33. Fechter-Leggett ED, Vaidyanathan A, Choudhary E. Heat Stress Illness Emergency Department Visits in National Environmental Public Health Tracking States, 2005–2010. J Community Health. 2016;41(1):57–69.

34. Fletcher BA, Lin S, Fitzgerald EF, Hwang SA. Association of summer temperatures with hospital admissions for renal diseases in New York state: A case-crossover study. Am J Epidemiol. 2012;175(9):907–16.

35. Fuhrmann CM, Sugg MM, Konrad CE, Waller A. Impact of Extreme Heat Events on Emergency Department Visits in North Carolina (2007–2011). J Community Health. 2016;41(1):146–56.

36. Goldberg MS, Gasparrini A, Armstrong B, Valois MF. The short-term influence of temperature on daily mortality in the temperate climate of Montreal, Canada. Environ Res [Internet]. 2011;111(6):853–60. Available from: http://dx.doi.org/10.1016/j.envres.2011.05.022

37. Golden JS, Hartz D, Brazel A, Luber G, Phelan P. A biometeorology study of climate and heat-related morbidity in Phoenix from 2001 to 2006. Int J Biometeorol. 2008;52(6):471–80.

38. Green RS, Basu R, Malig B, Broadwin R, Kim JJ, Ostro B. The effect of temperature on hospital admissions in nine California counties. Int J Public Health. 2010;55(2):113–21.

39. Gronlund CJ, Berrocal VJ, White-Newsome JL, Conlon KC, O’Neill MS. Vulnerability to extreme heat by socio-demographic characteristics and area green space among the elderly in Michigan, 1990-2007. Environ Res [Internet]. 2015;136:449–61. Available from: http://dx.doi.org/10.1016/j.envres.2014.08.042

40. Gronlund CJ, Zanobetti A, Schwartz JD, Wellenius GA, O’Neill MS. Heat, heat waves, and hospital admissions among the elderly in the United States, 1992–2006. Environ Health Perspect. 2014;122(11):1187–92.

41. Ha S, Talbott EO, Kan H, Prins CA, Xu X. The effects of heat stress and its effect modifiers on stroke hospitalizations in Allegheny County, Pennsylvania. Int Arch Occup Environ Health. 2014;87(5):557–65.

42. Ha S, Zhu Y, Kim SS, Mendola P, Liu D, Sherman S. Ambient Temperature and Early Delivery of Singleton Pregnancies. Environ Health Perspect [Internet]. 2017;125(3):453–9. Available from: http://10.0.5.9/EHP97%0Ahttp://search.ebscohost.com/login.aspx?direct=true&db=asx&AN=121523741&site=eds-live

43. Ha S, Zhu Y, Liu D, Sherman S, Mendola P. Ambient temperature and air quality in relation to small for gestational age and term low birthweight. Environ Res [Internet]. 2017;155(February):394–400. Available from: http://dx.doi.org/10.1016/j.envres.2017.02.021

44. Harlan SL, Chowell G, Yang S, Petitti DB, Butler EJM, Ruddell BL, et al. Heat-related deaths in hot cities: Estimates of human tolerance to high temperature thresholds. Int J Environ Res Public Health. 2014;11(3):3304–26.

45. Heidari L, Winquist A, Klein M, O’Lenick C, Grundstein A, Sarnat SE. Susceptibility to heat-related fluid and electrolyte imbalance emergency department visits in Atlanta, Georgia, USA. Int J Environ Res Public Health. 2016;13(10):18–20.

46. Henderson SB, Gauld JS, Rauch SA, McLean KE, Krstic N, Hondula DM, et al. A proposed case-control framework to probabilistically classify individual deaths as expected or excess during extreme hot weather events. Environ Heal A Glob Access Sci Source [Internet]. 2016;15(1):1–10. Available from: http://dx.doi.org/10.1186/s12940-016-0195-z

47. Henderson SB, Wan V, Kosatsky T. Differences in heat-related mortality across four ecological regions with diverse urban, rural, and remote populations in British Columbia, Canada. Heal Place [Internet]. 2013;23:48–53. Available from: http://dx.doi.org/10.1016/j.healthplace.2013.04.005

48. Hess JJ, Saha S, Luber G. Summertime acute heat illness in U.S. emergency departments from 2006 through 2010: Analysis of a nationally representative sample. Environ Health Perspect. 2014;122(11):1209–16.

49. Ho HC, Knudby A, Walker BB, Henderson SB. Delineation of spatial variability in the temperature-mortality relationship on extremely hot days in greater Vancouver, Canada. Environ Health Perspect. 2017;125(1):66–75.

50. Hondula . DM, Davis RE, Rocklöv J, Saha M V. A time series approach for evaluating intra-city heat-related mortality. J Epidemiol Community Health. 2013;67(8):707–12.

51. Hondula DM, Davis RE, Leisten MJ, Saha M V., Veazey LM, Wegner CR. Fine-scale spatial variability of heat-related mortality in Philadelphia County, USA, from 1983-2008: A case-series analysis. Environ Heal A Glob Access Sci Source [Internet]. 2012;11(1):16. Available from: http://www.ehjournal.net/content/11/1/16

52. Hondula DM, Davis RE, Saha M V., Wegner CR, Veazey LM. Geographic dimensions of heat-related mortality in seven U.S. cities. Environ Res [Internet]. 2015;138:439–52. Available from: http://dx.doi.org/10.1016/j.envres.2015.02.033

53. Hopp S, Dominici F, Bobb JF. Medical diagnoses of heat wave-related hospital admissions in older adults. Prev Med (Baltim) [Internet]. 2018;110(February):81–5. Available from: http://linkinghub.elsevier.com/retrieve/pii/S0091743518300288

54. Hoshiko S, English ÆP, Smith ÆD. A simple method for estimating excess mortality due to heat waves , as applied to the 2006 California heat wave. Intern J Public Heal. 2010;55:133–7.

55. Isaksen TB, Fenske RA, Hom EK, Ren Y, Lyons H, Yost MG. Increased mortality associated with extreme-heat exposure in King County, Washington, 1980–2010. Int J Biometeorol. 2016;60(1):85–98.

56. Jagai JS, Grossman E, Navon L, Sambanis A, Dorevitch S. Hospitalizations for heat-stress illness varies between rural and urban areas: An analysis of Illinois data, 1987-2014. Environ Heal A Glob Access Sci Source. 2017;16(1):1–10.

57. Jian Y, Wu CYH, Gohlke JM. Effect modification by environmental quality on the association between heatwaves and mortality in Alabama, United States. Int J Environ Res Public Health. 2017;14(10).

58. Joe L, Hoshiko S, Dobraca D, Jackson R, Smorodinsky S, Smith D, et al. Mortality during a Large-Scale Heat Wave by Place, Demographic Group, Internal and External Causes of Death, and Building Climate Zone Lauren. Int J Environ Res Public Health. 2016;13:299.

59. Kent ST, McClure LA, Zaitchik BF, Smith TT, Gohlke JM. Heat waves and health outcomes in Alabama (USA): The importance of heat wave definition. Environ Health Perspect. 2014;122(2):151–8.

60. Kingsley SL, Eliot MN, Gold J, Vanderslice RR, Wellenius GA. Current and projected heat-related morbidity and mortality in Rhode Island. Environ Health Perspect. 2016;124(4):460–7.

61. Klein Rosenthal J, Kinney PL, Metzger KB. Intra-urban vulnerability to heat-related mortality in New York City, 1997-2006. Heal Place. 2014;30:45–60.

62. Knowlton K, Rotkin-Ellman M, King G, Margolis HG, Smith D, Solomon G, et al. The 2006 California heat wave: Impacts on hospitalizations and emergency department visits. Environ Health Perspect. 2009;117(1):61–7.

63. Kosatsky T, Henderson SB, Pollock SL. Shifts in mortality during a hot weather event in Vancouver, British columbia: Rapid assessment with case-only analysis. Am J Public Health. 2012;102(12):2367–71.

64. Laverdière É, Généreux M, Gaudreau P, Morais JA, Shatenstein B, Payette H. Prevalence of risk and protective factors associated with heat-related outcomes in Southern Quebec: A secondary analysis of the NuAge study. Can J Public Heal. 2015;106(5):e315–21.

65. Lavigne E, Gasparrini A, Wang X, Chen H, Yagouti A, Fleury MD, et al. Extreme ambient temperatures and cardiorespiratory emergency room visits: Assessing risk by comorbid health conditions in a time series study. Environ Heal A Glob Access Sci Source [Internet]. 2014;13(1):1–8. Available from: Environmental Health

66. Lee M, Shi L, Zanobetti A, Schwartz JD. Study on the association between ambient temperature and mortality using spatially resolved exposure data. Environ Res [Internet]. 2016;151:610–7. Available from: http://dx.doi.org/10.1016/j.envres.2016.08.029

67. Lin S, Insaf TZ, Luo M, Hwang SA. The effects of ambient temperature variation on respiratory hospitalizations in summer, New York State. Int J Occup Environ Health. 2012;18(3):188–97.

68. Lin S, Luo M, Walker RJ, Liu X, Hwang SA, Chinery R. Extreme high temperatures and hospital admissions for respiratory and cardiovascular diseases. Epidemiology. 2009;20(5):738–46.

69. Lippmann SJ, Fuhrmann CM, Waller AE, Richardson DB. Ambient temperature and emergency department visits for heat-related illness in North Carolina, 2007-2008. Environ Res. 2013;124:35–42.

70. Liu X, Bertazzon S. Exploratory temporal and spatial analysis of myocardial infarction hospitalizations in Calgary, Canada. Int J Environ Res Public Health. 2017;14(12).

71. Madrigano J, Ito K, Johnson S, Kinney PL, Matte T. A case-only study of vulnerability to heat wave–related mortality in New York City (2000–2011). Environ Health Perspect. 2015;123(7):672–8.

72. Madrigano J, Jack D, Anderson GB, Bell ML, Kinney PL. Temperature , ozone , and mortality in urban and non-urban counties in the northeastern United States. Environ Heal. 2015;14(3):1–11.

73. Madrigano J, Mittleman AM, Baccarelli A, Goldberg R, Melly S, Klot S Von, et al. Temperature, Myocardial Infarction, and Mortality: Effect Modification by Individual and Area-Level Characteristics. Epidemiology. 2014;24(3):439–46.

74. Medina-Ramón M, Schwartz J. Temperature, temperature extremes, and mortality: A study of acclimatisation and effect modification in 50 US cities. Occup Environ Med. 2007;64(12):827–33.

75. Ngo NS, Horton RM. Climate change and fetal health: The impacts of exposure to extreme temperatures in New York City. Environ Res [Internet]. 2016;144:158–64. Available from: http://dx.doi.org/10.1016/j.envres.2015.11.016

76. Noelke C, McGovern M, Corsi DJ, Jimenez MP, Stern A, Wing IS, et al. Increasing ambient temperature reduces emotional well-being. Environ Res [Internet]. 2016;151:124–9. Available from: http://dx.doi.org/10.1016/j.envres.2016.06.045

77. O’Lenick CR, Winquist A, Chang HH, Kramer MR, Mulholland JA, Grundstein A, et al. Evaluation of individual and area-level factors as modifiers of the association between warm-season temperature and pediatric asthma morbidity in Atlanta, GA. Environ Res [Internet]. 2017;156(March):132–44. Available from: http://dx.doi.org/10.1016/j.envres.2017.03.021

78. Ostro BD, Roth LA, Green RS, Basu R. Estimating the mortality effect of the July 2006 California heat wave. Environ Res [Internet]. 2009;109(5):614–9. Available from: http://dx.doi.org/10.1016/j.envres.2009.03.010

79. Ostro B, Rauch S, Green R, Malig B, Basu R. The effects of temperature and use of air conditioning on hospitalizations. Am J Epidemiol. 2010;172(9):1053–61.

80. Pengelly LD, Campbell ME, Cheng CS, Gingrich SE, Macfarlane R. Anatomy of Heat Waves and Lessons for Public Health Protection. Can J Public Heal. 2007;98(5):1–6.

81. Petitti DB, Harlan SL, Chowell-Puente G, Ruddell D. Occupation and Environmental Heat-Associated Deaths in Maricopa County, Arizona: A Case-Control Study. PLoS One. 2013;8(5).

82. Petitti DB, Hondula DM, Yang S, Harlan SL, Chowell G. Multiple trigger points for quantifying heat-health impacts: New evidence from a hot climate. Environ Health Perspect. 2016;124(2):176–83.

83. Petkova EP, Gasparrini A, Kinney PL. Heat and mortality in new york city since the beginning of the 20th century. Epidemiology. 2014;25(4):554–60.

84. Pillai SK, Noe RS, Murphy MW, Vaidyanathan A, Young R, Kieszak S, et al. Heat illness: Predictors of hospital admissions among emergency department visits - Georgia, 2002-2008. J Community Health. 2014;39(1):90–8.

85. S. H, D. L, Y. Z, S.S. K, S. S, K.L. G. Ambient temperature and stillbirth: A multi-center retrospective cohort study. Environ Health Perspect [Internet]. 2017;125(6):67011. Available from: https://ehp.niehs.nih.gov/wp-content/uploads/2017/06/EHP945.alt_.pdf%0Ahttp://ovidsp.ovid.com/ovidweb.cgi?T=JS&PAGE=reference&D=emexb&NEWS=N&AN=619170033

86. Saha S, Brock JW, Vaidyanathan A, Easterling DR, Luber G. Spatial variation in hyperthermia emergency department visits among those with employerbased insurance in the United States - A casecrossover analysis. Environ Heal A Glob Access Sci Source. 2015;14(1):1–9.

87. Sherbakov T, Malig B, Guirguis K, Gershunov A, Basu R. Ambient temperature and added heat wave effects on hospitalizations in California from 1999 to 2009. Environ Res [Internet]. 2018;160(May 2017):83–90. Available from: https://doi.org/10.1016/j.envres.2017.08.052

88. Sheridan SC, Lin S. Assessing Variability in the Impacts of Heat on Health Outcomes in New York City Over Time, Season, and Heat-Wave Duration. Ecohealth. 2014;11(4):512–25.

89. Smargiassi A, Goldberg MS, Plante C, Fournier M, Baudouin Y, Kosatsky T. Variation of daily warm season mortality as a function of micro-urban heat islands. J Epidemiol Community Health. 2009;63(8):659–64.

90. Sugg MM, Konrad CE, Fuhrmann CM. Relationships between maximum temperature and heat-related illness across North Carolina, USA. Int J Biometeorol [Internet]. 2016;60(5):663–75. Available from: http://dx.doi.org/10.1007/s00484-015-1060-4

91. Uejio CK, Tamerius JD, Vredenburg J, Asaeda G, Isaacs DA, Braun J, et al. Summer indoor heat exposure and respiratory and cardiovascular distress calls in New York City, NY, U.S. Indoor Air. 2016;26(4):594–604.

92. Uejio CK, Wilhelmi O V., Golden JS, Mills DM, Gulino SP, Samenow JP. Intra-urban societal vulnerability to extreme heat: The role of heat exposure and the built environment, socioeconomics, and neighborhood stability. Heal Place [Internet]. 2011;17(2):498–507. Available from: http://dx.doi.org/10.1016/j.healthplace.2010.12.005

93. van Zutphen AR, Lin S, Fletcher BA, Hwang SA. A population-based case-control study of extreme summer temperature and birth defects. Environ Health Perspect. 2012;120(10):1443–9.

94. Vida S, Sc M, Durocher M, Sc M. Relationship Between Ambient Temperature and Humidity and Visits to Mental Health Emergency Departments in Québec. Psychiatr Serv. 2012;63(11).

95. Wang X, Lavigne E, Ouellette-Kuntz H, Chen BE. Acute impacts of extreme temperature exposure on emergency room admissions related to mental and behavior disorders in Toronto, Canada. J Affect Disord [Internet]. 2014;155(1):154–61. Available from: http://dx.doi.org/10.1016/j.jad.2013.10.042

96. Wang Y, Bobb JF, Papi B, Wang Y, Kosheleva A, Di Q, et al. Heat stroke admissions during heat waves in 1,916 US counties for the period from 1999 to 2010 and their effect modifiers. Environ Heal A Glob Access Sci Source [Internet]. 2016;15(1):1–9. Available from: http://dx.doi.org/10.1186/s12940-016-0167-3

97. Wellenius GA, Eliot MN, Bush KF, Holt D, Lincoln RA, Smith AE, et al. Heat-related morbidity and mortality in New England: Evidence for local policy. Environ Res [Internet]. 2017;156(February):845–53. Available from: http://dx.doi.org/10.1016/j.envres.2017.02.005

98. Winquist A, Grundstein A, Chang HH, Hess J, Sarnat SE. Warm season temperatures and emergency department visits in Atlanta, Georgia. Environ Res [Internet]. 2016;147:314–23. Available from: http://dx.doi.org/10.1016/j.envres.2016.02.022

99. Zanobetti A, O’Neill MS, Gronlund CJ, Schwartz JD. Susceptibility to Mortality in Weather Extremes. Epidemiology [Internet]. 2013;24(6):809–19. Available from: http://content.wkhealth.com/linkback/openurl?sid=WKPTLP:landingpage&an=00001648-201311000-00004

100. Zhang K, Chen TH, Begley CE. Impact of the 2011 heat wave on mortality and emergency department visits in Houston, Texas -No section-. Environ Heal A Glob Access Sci Source. 2015;14(1):1–7.

**Flooding systematic review:**

**Figure 2b** summarises the results of the systematic review for flooding. In total, the search from the two databases returned 1749 papers using the following search terms:

| *Coastal flood*OR flood* OR inundation OR river flood* OR SLR OR storm surge*  *AND*  *Accident* OR contamination OR death* OR disease* OR environmental health OR epidemic* OR illness OR morbidity OR mortality OR outbreak* OR physical health OR pollut* OR public health OR stress OR vector* OR water** |
| --- |

After removing duplicates and studies that did not meet the primary inclusion, 147 abstracts were reviewed. Studies were further removed when the focus of the paper was in a different subject area (e.g. toxicology, emergency management) and when health impacts were not related to flooding. 37 epidemiological papers were reviewed in the final inclusion.

Figure 2b – Flow chart of results from the systematic review of flooding:


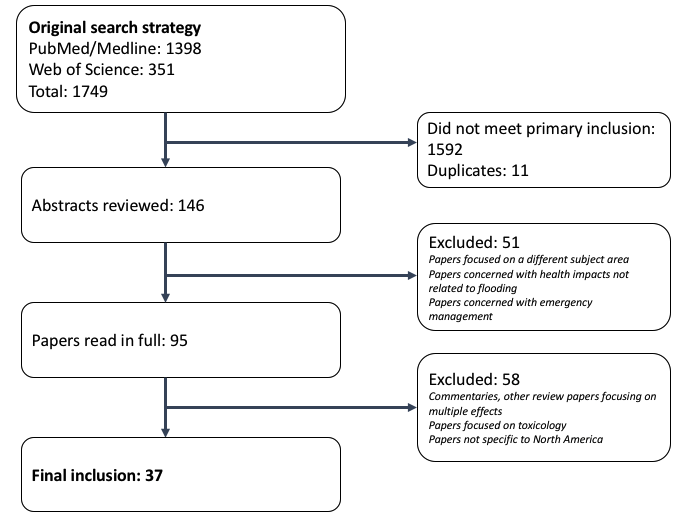


**List of final papers for flooding:**

1. Akerlof KL, Delamater PL, Boules CR, Upperman CR, Mitchell CS. Vulnerable populations perceive their health as at risk from climate change. Int J Environ Res Public Health. 2015;12(12):15419–33.

2. Brackbill RM, Caramanica K, Maliniak M, Stellman SD, Fairclough MA. Nonfatal Injuries 1 Week After Hurricane Sandy — New York City Metropolitan Area , October 2012. Morb Mortal Wkly Rep. 2014;63(42):950–4.

3. Cardenas VM, Jaime J, Ford PB, J. F, Gonzalez, Carrillo I, et al. Yard flooding by irrigation canals increased the risk of West Nile disease in El Paso, Texas. Ann Epidemiol. 2015;85(0 1):1–27.

4. Collins TW, Jimenez AM, Grineski SE. Hispanic Health Disparities After a Flood Disaster : Results of a Population-Based Survey of Individuals Experiencing Home Site Damage in El Paso ( Texas , USA ). J Immigr Minor Heal. 2013;15:415–26.

5. Dancause KN, Laplante DP, Hart KJ, Hara MWO, Elgbeili G, Brunet A, et al. Prenatal Stress due to a Natural Disaster Predicts Adiposity in Childhood : The Iowa Flood Study. J Obes. 2015;2015:1–10.

6. Diakakis M, Deligiannakis G, Katsetsiadou K, Lekkas E. Hurricane Sandy mortality in the Caribbean and continental North America. Disaster Prev Manag. 2015;24(1):132–48.

7. Doran KM, Mccormack RP, Johns EL, Carr BG, Smith SW, Goldfrank LR, et al. Emergency Department Visits for Homelessness or Inadequate Housing in New York City before and after Hurricane Sandy. J Urban Heal. 2016;93(2):331–44.

8. Dursun P, Steger MF, Bentele C, Schulenberg SE. Meaning and Posttraumatic Growth Among Survivors of the September 2013 Colorado Floods. J Clin Psychol. 2016;72(September 2013):1247–63.

9. Ehrlich M, Harville E, Xiong X, Buekens P, Pridjian G, Elkind-Hirsch K. Loss of Resources and Hurricane Experience as Predictors of Postpartum Depression Among Women in Southern Louisiana. J Women’s Heal. 2010;19(5):877–84.

10. Eiffert S, Noibi Y, Vesper S, Downs J, Fulk F, Wallace J, et al. A Citizen-Science Study Documents Environmental Exposures and Asthma Prevalence in Two Communities. J Environ Public Health. 2016;2016.

11. Gotanda H, Fogel J, Husk G, Levine JM, Peterson M, Baumlin K, et al. Hurricane Sandy : Impact on Emergency Department and Hospital Utilization by Older Adults in Lower Manhattan , New York ( USA ). Prehosp Disaster Med. 2018;30(5).

12. Grimsley LF, Chulada PC, Kennedy S, White L, Wildfire J, Cohn RD, et al. Indoor Environmental Exposures for Children with Asthma Enrolled in the HEAL Study, Post-Katrina New Orleans. Child Heal. 2012;1600(11):1600–6.

13. Hamilton BE, Ph D, Sutton PD, Ph D, Mathews TJ, Martin JA, et al. National Vital Statistics Reports The Effect of Hurricane Katrina : Births in the U . S . Gulf Coast Region , Before and After the Storm. Natl Vital Stat Reports. 2009;58(2):1–9.

14. Harrison BA, Whitt PB, Roberts LF, Jennifer A, Lindsey NP, Nasci RS, et al. Rapid Assessment of Mosquitoes and Arbovirus Activity after Floods in Southeastern Kansas , 2007 Your use of this PDF , the BioOne Web site , and all posted and associated content RAPID ASSESSMENT OF MOSQUITOES AND ARBOVIRUS ACTIVITY AFTER FLOODS IN SOUTH. J Am Mosq Control Assoc. 2007;25(3):265–71.

15. Hoppe KA, Metwali1 N, Perry SS, Hart T, Kostle PA, Thorne PS. Assessment of airborne exposures and health in flooded homes undergoing renovation. Indoor Air. 2012;22:446–56.

16. Horney JA, Casillas GA, Baker E, Stone KW, Kirsch R, Camargo K, et al. Comparing residential contamination in a Houston environmental justice neighborhood before and after Hurricane Harvey. PLoS One. 2018;13(2):1–16.

17. Jonkman SN, Maaskant B, Boyd E, Levitan ML. Loss of Life Caused by the Flooding of New Orleans After Hurricane Katrina : Analysis of the Relationship Between Flood Characteristics and Mortality. Risk Anal. 2009;29(5):676–98.

18. King R V, Polatin PB, Hogan D, Downs DL, North CS. Needs Assessment of Hurricane Katrina Evacuees Residing Temporarily in Dallas. Community Ment Health J. 2016;52(1):18–24.

19. Lieberman-Cribbin W, Liu B, Schneider S, Schwartz R, Taioli E. Self-reported and FEMA flood exposure assessment after hurricane sandy: Association with mental health outcomes. PLoS One. 2017;12(1):1–15.

20. Lin CJ, Wade TJ, Hilborn ED. Flooding and Clostridium difficile infection: A case-crossover analysis. Int J Environ Res Public Health. 2015;12(6):6948–64.

21. Liu H, Behr JG, Diaz R. Population Vulnerability to Storm Surge Flooding in Coastal Virginia, USA. Integr Env Assess Manag. 2016;12(3):500–9.

22. Maldonado A, Collins TW, Grineski SE, Chakraborty J. Exposure to Flood Hazards in Miami and Houston : Are Hispanic Immigrants at Greater Risk than Other Social Groups ? Int J Environ Res Public Heal. 2016;

23. Martin NC, Felton JW, Cole DA, Martin NC, Felton JW, Cole DA, et al. Predictors of Youths ’ Posttraumatic Stress Symptoms Following a Natural Disaster : The 2010 Predictors of Youths ’ Posttraumatic Stress Symptoms Following a Natural Disaster : The 2010 Nashville , Tennessee , Flood. J Clin Child Adolesc Psychol. 2016;4416(45):3.

24. McLaughlin KA, Fairbank JA, Gruber MJ, Jones RT, Osofsky JD, Pfefferbaum B, et al. Trends in Serious Emotional Disturbance among Youths Exposed to Hurricane Katrina. J Am Acad Child Adolesc Psychiatry. 2011;49(10):1–18.

25. Mielke HW, Gonzales CR, Powell ET. Soil Lead and Children’s Blood Lead Disparities in Pre- and Post-Hurricane Katrina New Orleans (USA). Int J Environ Res Public Heal. 2017;14.

26. Moise IK, Ruiz MO. Hospitalizations for Substance Abuse Disorders Before and After Hurricane Katrina : Spatial Clustering and Area-Level. Prev Chronic Dis Public Heal Res Pract Policy. 2016;13(145):1–11.

27. Peek-asa C, Ramirez M, Young T, Cao Y. Flood-Related Work Disruption and Poor Health Outcomes Among University Students. Prehosp Disaster Med. 2018;(December 2012).

28. Quinlisk P, Jones MJ, Bostick NA, Walsh LE, Curtiss R, Walker R, et al. Results of Rapid Needs Assessments in Rural and Urban Iowa Following Large-scale Flooding Events in 2008. Disaster Med Public Health Prep. 2018;5(4):287–92.

29. Rando RJ, Kwon C-W, Lefante JJ. Exposures to Thoracic Particulate Matter, Endotoxin, and Glucan During Post-Hurricane Katrina Restoration Work, New Orleans 2005–2012. J Occup Environ Hyg. 2014;11(1):9–18.

30. Rath B, Young EA, Harris A, Perrin K, Bronfin DR, Ratard R, et al. Adverse Respiratory Symptoms and Environmental Exposures Among Children and Adolescents Following Hurricane Katrina. Public Health Rep. 2011;126:853–60.

31. Sarfaty M, Mitchell M, Bloodhart B, Maibach EW. A Survey of African American Physicians on the Health Effects of Climate Change. Int J Environ Res Public Health. 2014;11:12473–85.

32. Soneja S, Jiang C, Romeo C, Murtugudde R, Mitchell CS, Blythe D, et al. Extreme precipitation events and increased risk of campylobacteriosis in Maryland , U . S . A. Environ Res. 2016;149:216–21.

33. Tong VT, Zotti ME, Hsia J. Impact of the red river catastrophic flood on women giving birth in North Dakota, 1994-2000. Matern Child Health J. 2011;15(3):281–8.

34. Vanasse A, Cohen A, Courteau J, Bergeron P, Dault R, Gosselin P, et al. Association between floods and acute cardiovascular diseases: A population-based cohort study using a geographic information system approach. Int J Environ Res Public Health. 2016;13(2):1–12.

35. Wade TJ, Lin CJ, Jagai JS, Hilborn ED. Flooding and Emergency Room Visits for Gastrointestinal Illness in Massachusetts : A Case-Crossover Study. PLoS One. 2014;9(10):1–9.

36. Zahran S, Brody S, Peacock W, Vedlitz A, Grover H. Social vulnerability and the natural and built environment: a model of flood casualties in Texas. Disasters. 2008;32(4):537–60.

37. Zane DF, Bayleyegn TM, Hellsten J, Beal R, Beasley C, Haywood T, et al. Tracking Deaths Related to Hurricane Ike, Texas, 2008. Disaster Med Public Health Prep. 2011;5:23–8.

**Wildfire smoke systematic review:**

**Figure 2c** summarises the results of the systematic review for wildfire smoke. In total, the search from the two databases returned 1315 papers using the following search terms:

| *forest fire*OR wildfire* OR bushfire OR peat bog fire OR urban fire OR landscape fire OR grassfire OR vegetation fire*  *AND*  *health OR respir* OR pulmon* OR asthm* OR hospital* OR emergency* OR mortality OR cardiac OR cardiovascular OR reproductive OR mental health OR vulnerable OR vulnerability OR stress OR morbidity* |
| --- |

After removing duplicates and studies that did not meet the primary inclusion, 542 abstracts were reviewed. Studies were further removed when the focus of the paper was in a different subject area (e.g. toxicology, biochemistry, molecular biology, genetics, health burden/economic costs) and not related to human exposure to wildfire smoke. 25 epidemiological papers were reviewed in the final inclusion.

Figure 2c – Flow chart of results from the systematic review of wildfire smoke


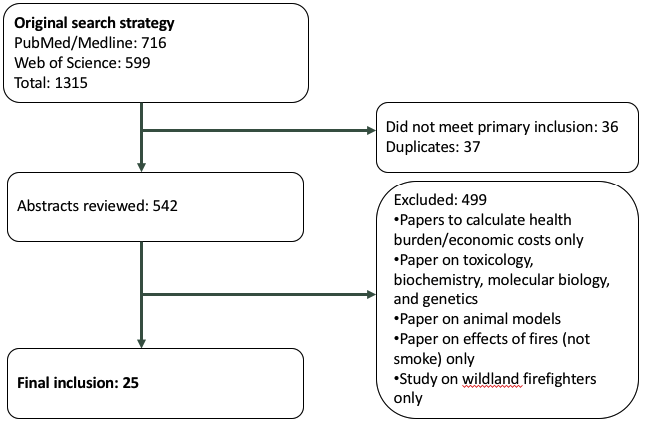


List of final papers for wildfire smoke:

1. Zu K, Tao G, Long C, Goodman J, Valberg P. Long-range fine particulate matter from the 2002 Quebec forest fires and daily mortality in Greater Boston and New York City. Air Qual Atmos Heal. 2016;9(3):213–21.

2. Yao J, Eyamie J, Henderson SB. Evaluation of a spatially resolved forest fire smoke model for population-based epidemiologic exposure assessment. J Expo Sci Environ Epidemiol [Internet]. 2016;26(3):233–40. Available from: http://dx.doi.org/10.1038/jes.2014.67

3. Yao J, Brauer M, Henderson SB. Evaluation of a wildfire smoke forecasting system as a tool for public health protection. Environ Health Perspect. 2013;121(10):1142–7.

4. Tse K, Chen L, Tse M, Zuraw B, Christiansen S. Effect of catastrophic wildfires on asthmatic outcomes in obese children: Breathing fire. Ann Allergy Asthma Immunol. 2017;114(4):308–11.

5. Tinling MA, West JJ, Cascio WE, Kilaru V, Rappold AG. Repeating cardiopulmonary health effects in rural North Carolina population during a second large peat wildfire. Environ Heal A Glob Access Sci Source [Internet]. 2016;15(1):1–12. Available from: http://dx.doi.org/10.1186/s12940-016-0093-4

6. Thelen B, French NH, Koziol BW, Billmire M, Owen RC, Johnson J, et al. Modeling acute respiratory illness during the 2007 San Diego wildland fires using a coupled emissions-transport system and generalized additive modeling. Environ Heal A Glob Access Sci Source. 2013;12(1):1–22.

7. Schranz CI, Castillo EM, Vilke GM. The 2007 San Diego wildfire impact on the emergency Department of the University of California, San Diego hospital system. Prehosp Disaster Med. 2010;25(5):472–6.

8. Resnick A, Woods B, Krapfl H, Toth B. Health outcomes associated with smoke exposure in Albuquerque, New Mexico, during the 2011 Wallow fire. J Public Heal Manag Pract. 2015;21:S55–61.

9. Reid CE, Jerrett M, Tager IB, Petersen ML, Mann JK, Balmes JR. Differential respiratory health effects from the 2008 northern California wildfires: A spatiotemporal approach. Environ Res [Internet]. 2016;150:227–35. Available from: http://dx.doi.org/10.1016/j.envres.2016.06.012

10. Rappold AG, Reyes J, Pouliot G, Cascio WE, Diaz-Sanchez D. Community Vulnerability to Health Impacts of Wildland Fire Smoke Exposure. Environ Sci Technol. 2017;51(12):6674–82.

11. A.G. R, W.E. C, V.J. K, S.L. S, L.M. N, R.B. D, et al. Cardio-respiratory outcomes associated with exposure to wildfire smoke are modified by measures of community health. Environ Heal A Glob Access Sci Source [Internet]. 2012;11(1):71. Available from: http://www.embase.com/search/results?subaction=viewrecord&from=export&id=L366142879%0Ahttp://dx.doi.org/10.1186/1476-069X-11-71%0Ahttp://sfxhosted.exlibrisgroup.com/sfxtul?sid=EMBASE&issn=1476069X&id=doi:10.1186%2F1476-069X-11-71&atitle=Cardio-respiratory+out

12. Rappold AG, Stone SL, Cascio WE, Neas LM, Kilaru VJ, Carraway MS, et al. Peat bog wildfire smoke exposure in rural North Carolina is associated with cardiopulmonary emergency department visits assessed through syndromic surveillance. Environ Health Perspect. 2011;119(10):1415–20.

13. Moeltner K, Kim MK, Zhu E, Yang W. Wildfire smoke and health impacts: A closer look at fire attributes and their marginal effects. J Environ Econ Manage [Internet]. 2013;66(3):476–96. Available from: http://dx.doi.org/10.1016/j.jeem.2013.09.004

14. Mirabelli MC, Künzlia N, Avole E, Gillilande FD, Gaudermane WJ, McConnelle R, et al. Respiratory Symptoms Following Wildfire Smoke Exposure: Airway Size as a Susceptibility Factor. Epidemiology. 2015;27(4):215–25.

15. Liu JC, Wilson A, Mickley LJ, Ebisu K, Sulprizio MP, Wang Y, et al. Who among the Elderly Is Most Vulnerable to Exposure to and Health Risks of Fine Particulate Matter from Wildfire Smoke? Am J Epidemiol. 2017;186(6):730–5.

16. Liu JC, Wilson A, Mickley LJ, Dominici F, Ebisu K, Wang Y, et al. Wildfire-specific fine particulate matter and risk of hospital admissions in urban and rural counties. Epidemiology. 2017;28(1):77–85.

17. Lee TS, Falter K, Meyer P, Mott J, Gwynn C. Risk factors associated with clinic visits during the 1999 forest fires near the Hoopa Valley Indian Reservation, California, USA. Int J Environ Health Res. 2009;19(5):315–27.

18. Le G, Breysse P, McDermott A, Eftim S, Geyh A, Berman J, et al. Canadian Forest Fires and the Effects of Long-Range Transboundary Air Pollution on Hospitalizations among the Elderly. ISPRS Int J Geo-Information [Internet]. 2014;3(2):713–31. Available from: http://www.mdpi.com/2220-9964/3/2/713/

19. Holstius DM, Reid CE, Jesdale BM, Morello-Frosch R. Birth weight following pregnancy during the 2003 southern California wildfires. Environ Health Perspect. 2012;120(9):1340–5.

20. Henderson SB, Brauer M, MacNab YC, Kennedy SM. Three measures of forest fire smoke exposure and their associations with respiratory and cardiovascular health outcomes in a population-based cohort. Environ Health Perspect. 2011;119(9):1266–71.

21. Gan RW, Ford B, Lassman W, Gabriele P, Vaidyanathan A, Fischer E, et al. Comparison of wildfire smoke estimation methods and associations with cardiopulmonary-related hospital admissions. GeoHealth. 2017;1:122–36.

22. C.T. E, S.B. H, V. W. Time series analysis of fine particulate matter and asthma reliever dispensations in populations affected by forest fires. Environ Heal A Glob Access Sci Source [Internet]. 2014;12(1):1–9. Available from: http://www.embase.com/search/results?subaction=viewrecord&from=export&id=L600372386%0Ahttp://dx.doi.org/10.1186/1476-069X-12-11%0Ahttps://tdnetdiscover.com/shibboleth/go/773/resolver/full?sid=EMBASE&issn=1476069X&id=doi:10.1186/1476-069X-12-11&atitle=Time+s

23. Dohrenwend P, Le M, Bush J, Thomas C. The Impact on Emergency Department Visits for Respiratory Illness During the Southern California Wildfires. West J Emerg Med [Internet]. 2013;14(2):79–84. Available from: http://www.escholarship.org/uc/item/0ds9h7j2#page-1

24. Delfino RJ, Brummel S, Wu J, Stern H, Ostro B, Lipsett M, et al. The relationship of respiratory and cardiovascular hospital admissions to the southern California wildfires of 2003. Occup Environ Med. 2009;66(3):189–97.

25. Alman BL, Pfister G, Hao H, Stowell J, Hu X, Liu Y, et al. The association of wildfire smoke with respiratory and cardiovascular emergency department visits in Colorado in 2012: A case crossover study. Environ Heal A Glob Access Sci Source [Internet]. 2016;15(1):1–9. Available from: http://dx.doi.org/10.1186/s12940-016-0146-8

**Ozone systematic review:**

**Figure 2d** summarises the results of the systematic review for ozone. In total, the searches from the two databases returned 23 164 papers using the following search terms:

Search 1

| *modif* AND (effect OR effects) AND (ozone OR O3)* |
| --- |

Search 2

| *Ozone*  *AND*  *emergency department OR emergency visit* OR emergency room OR hospital* OR physician visit* OR mortality OR morbidity OR death OR health OR public health OR disease**  *OR respiratory OR pulmonary OR asthma OR lung function OR respiratory tract infection* OR COPD OR chronic obstructive pulmonary disease*  *OR cardiac OR cardiovascular OR stroke*  *OR reproductive OR developmental OR mental health OR immunity OR allerg* OR central nervous system OR cancer OR neoplasm OR symptom* OR medication*  *OR vulnerable population* OR vulnerability OR susceptible OR susceptibility* |
| --- |

MESH terms were included in addition to key words when the search was performed using PubMed/Medline. These terms were: "Ozone" [MESH], "Emergency Service, Hospital/" [Mesh], "Hospitalization"[Mesh], "Mortality" [Mesh], “Morbidity" [Mesh], "Death" [Mesh], "Health" [Mesh], "Public Health" [Mesh], "Disease" [Mesh], "Asthma" [Mesh], "Respiratory Tract Infections" [Mesh], "Pulmonary Disease, Chronic Obstructive/" [Mesh], "Stroke" [Mesh], "Mental Health" [Mesh], "Immunity" [Mesh], "Hypersensitivity" [Mesh], "Central Nervous System" [Mesh], "Neoplasms" [Mesh], "Vulnerable Populations" [Mesh], and "Disease Susceptibility" [Mesh].

After removing duplicates and studies that did not meet the primary inclusion, 733 abstracts were reviewed. Studies were further removed when the focus of the paper was in a different subject area (e.g. toxicology, genetics) and when health impacts were not related to ozone. 231 epidemiological papers were reviewed in the final inclusion.

Figure 2d – Flow chart of results from the systematic review of ozone


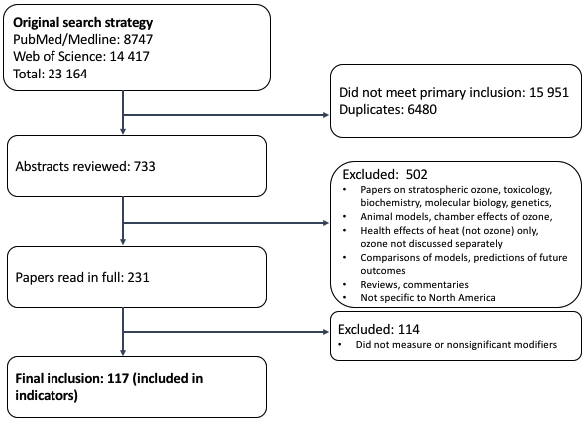


List of final papers for ozone:

1. Adam-Poupart A, Labreche F, Busque M, Brand A, Duguay P, Fournier M, et al. Association between outdoor ozone and compensated acute respiratory diseases among workers in Quebec (Canada). Ind Health 2015;53(2):171-175.
2. Alexeeff SE, Litonjua AA, Suh H, Sparrow D, Vokonas PS, Schwartz J. Ozone exposure and lung function: effect modified by obesity and airways hyperresponsiveness in the VA normative aging study. Chest 2007;132(6):1890-1897.
3. Alhanti BA, Chang HH, Winquist A, Mulholland JA, Darrow LA, Sarnat SE. Ambient air pollution and emergency department visits for asthma: a multi-city assessment of effect modification by age. J Expo Sci Environ Epidemiol 2016;26(2):180-188.
4. Amadeo B, Robert C, Rondeau V, Mounouchy M, Cordeau L, Birembaux X, et al. Impact of close-proximity air pollution on lung function in schoolchildren in the French West Indies. BMC Public Health 2015;15:45.
5. Babin SM, Burkom HS, Holtry RS, Tabernero NR, Stokes LD, Davies-Cole JO, et al. Pediatric patient asthma-related emergency department visits and admissions in Washington, DC, from 2001-2004, and associations with air quality, socio-economic status and age group. Environmental Health: A Global Access Science Source 2007;6:9.
6. Babin S, Burkom H, Holtry R, Tabernero N, Davies-Cole J, Stokes L, et al. Medicaid patient asthma-related acute care visits and their associations with ozone and particulates in Washington, DC, from 1994-2005. Int J Environ Health Res 2008;18(3):209-221.
7. Becerra TA, Wilhelm M, Olsen J, Cockburn M, Ritz B. Ambient air pollution and autism in Los Angeles county, California. Environ Health Perspect 2013;121(3):380-386.
8. Bell ML, Dominici F. Effect modification by community characteristics on the short-term effects of ozone exposure and mortality in 98 US communities. Am J Epidemiol 2008;167(8):986-997.
9. Berhane K, Chang C, McConnell R, Gauderman WJ, Avol E, Rapapport E, et al. Association of changes in air quality with bronchitic symptoms in children in California, 1993-2012. JAMA 2016;315(14):1491-1501.
10. Brown JM, Harris G, Pantea C, Hwang S, Talbot TO. Linking air pollution data and adverse birth outcomes: environmental public health tracking in New York State. J Public Health Manag Pract 2015;21(Suppl 2):S68-74.
11. Burra TA, Moineddin R, Agha MM, Glazier RH. Social disadvantage, air pollution, and asthma physician visits in Toronto, Canada. Environ Res 2009;109(5):567-574.
12. Byers N, Ritchey M, Vaidyanathan A, Brandt AJ, Yip F. Short-term effects of ambient air pollutants on asthma-related emergency department visits in Indianapolis, Indiana, 2007-2011. J Asthma 2016;53(3):245-252.
13. Cakmak S, Hebbern C, Vanos J, Crouse DL, Burnett R. Ozone exposure and cardiovascular-related mortality in the Canadian Census Health and Environment Cohort (CANCHEC) by spatial synoptic classification zone. Environ Pollut 2016;214:589-599.
14. Carbajal-Arroyo L, Miranda-Soberanis V, Medina-Ramon M, Rojas-Bracho L, Tzintzun G, Solis-Gutierrez P, et al. Effect of PM(10) and O(3) on infant mortality among residents in the Mexico City Metropolitan Area: a case-crossover analysis, 1997-2005. J Epidemiol Community Health 2011;65(8):715-721.
15. Chen C, Zhao B, Weschler CJ. Assessing the influence of indoor exposure to "outdoor ozone" on the relationship between ozone and short-term mortality in U.S. communities. Environ Health Perspect 2012;120(2):235-240.
16. Crouse DL, Peters PA, Hystad P, Brook JR, van Donkelaar A, Martin RV, et al. Ambient PM2.5, O3, and NO2 exposures and associations with mortality over 16 Years of follow-up in the Canadian Census Health and Environment Cohort (CanCHEC). Environ Health Perspect 2015;123(11):1180-1186.
17. Dales RE, Cakmak S. Does mental health status influence susceptibility to the physiologic effects of air pollution? A population based study of Canadian children. PLoS ONE [Electronic Resource] 2016;11(12):e0168931.
18. Darrow LA, Klein M, Flanders WD, Mulholland JA, Tolbert PE, Strickland MJ. Air pollution and acute respiratory infections among children 0-4 years of age: an 18-year time-series study. Am J Epidemiol 2014;180(10):968-977.
19. De Roos AJ, Koehoorn M, Tamburic L, Davies HW, Brauer M. Proximity to traffic, ambient air pollution, and community noise in relation to incident rheumatoid arthritis. Environ Health Perspect 2014;122(10):1075-1080.
20. Delfino RJ, Wu J, Tjoa T, Gullesserian SK, Nickerson B, Gillen DL. Asthma morbidity and ambient air pollution: effect modification by residential traffic-related air pollution. Epidemiology 2014;25(1):48-57.
21. Di Q, Dai L, Wang Y, Zanobetti A, Choirat C, Schwartz JD, et al. Association of short-term exposure to air pollution with mortality in older adults. JAMA 2017;318(24):2446-2456.
22. Di Q, Wang Y, Zanobetti A, Wang Y, Koutrakis P, Choirat C, et al. Air pollution and mortality in the Medicare population. N Engl J Med 2017;376(26):2513-2522.
23. Ebisu K, Bell ML. Airborne PM2.5 chemical components and low birth weight in the northeastern and mid-Atlantic regions of the United States. Environ Health Perspect 2012;120(12):1746-1752.
24. Eckel SP, Louis TA, Chaves PHM, Fried LP, Margolis AHG. Modification of the association between ambient air pollution and lung function by frailty status among older adults in the Cardiovascular Health Study. Am J Epidemiol 2012;176(3):214-223.
25. Ensor KB, Raun LH, Persse D. A case-crossover analysis of out-of-hospital cardiac arrest and air pollution. Circulation 2013;127(11):1192-1199.
26. Gleason JA, Bielory L, Fagliano JA. Associations between ozone, PM2.5, and four pollen types on emergency department pediatric asthma events during the warm season in New Jersey: a case-crossover study. Environ Res 2014;132:421-429.
27. Goodman JE, Loftus CT, Liu X, Zu K. Impact of respiratory infections, outdoor pollen, and socioeconomic status on associations between air pollutants and pediatric asthma hospital admissions. PLoS ONE [Electronic Resource] 2017;12(7):e0180522.
28. Gorai AK, Tchounwou PB, Tuluri F. Association between ambient air pollution and asthma prevalence in different population groups residing in eastern Texas, USA. Int J Environ Res Public Health [Electronic Resource] 2016;13(4):378.
29. Green R, Sarovar V, Malig B, Basu R. Association of stillbirth with ambient air pollution in a California cohort study. Am J Epidemiol 2015;181(11):874-882.
30. Ha S, Hu H, Roussos-Ross D, Haidong K, Roth J, Xu X. The effects of air pollution on adverse birth outcomes. Environ Res 2014;134:198-204.
31. Hackbarth AD, Romley JA, Goldman DP. Racial and ethnic disparities in hospital care resulting from air pollution in excess of federal standards. Soc Sci Med 2011;73(8):1163-1168.
32. Hankey S, Marshall JD, Brauer M. Health impacts of the built environment: within-urban variability in physical inactivity, air pollution, and ischemic heart disease mortality. Environ Health Perspect 2012;120(2):247-253.
33. Hanna AF, Yeatts KB, Xiu A, Zhu Z, Smith RL, Davis NN, et al. Associations between ozone and morbidity using the Spatial Synoptic Classification system. Environmental Health: A Global Access Science Source 2011;10:49.
34. Hebbern C, Cakmak S. Synoptic weather types and aeroallergens modify the effect of air pollution on hospitalisations for asthma hospitalisations in Canadian Cities. Environ Pollut 2015;204:9-16.
35. Hernandez-Cadena L, Holguin F, Barraza-Villarreal A, Del Rio-Navarro BE, Sienra-Monge JJ, Romieu I. Increased levels of outdoor air pollutants are associated with reduced bronchodilation in children with asthma. Chest 2009;136(6):1529-1536.
36. Hu H, Ha S, Xu X. Ozone and hypertensive disorders of pregnancy in Florida: Identifying critical windows of exposure. Environ Res 2017;153:120-125.
37. Jerrett M, Brook R, White LF, Burnett RT, Yu J, Su J, et al. Ambient ozone and incident diabetes: A prospective analysis in a large cohort of African American women. Environ Int 2017;102:42-47.
38. Jerrett M, Burnett RT, Pope CA3, Ito K, Thurston G, Krewski D, et al. Long-term ozone exposure and mortality. N Engl J Med 2009;360(11):1085-1095.
39. Jhun I, Fann N, Zanobetti A, Hubbell B. Effect modification of ozone-related mortality risks by temperature in 97 US cities. Environ Int 2014;73:128-134.
40. Jones RR, Oezkaynak H, Nayak SG, Garcia V, Hwang S, Lin S. Associations between summertime ambient pollutants and respiratory morbidity in New York City: Comparison of results using ambient concentrations versus predicted exposures. J Expo Sci Environ Epidemiol 2013;23(6):616-626.
41. Kaplan GG, Dixon E, Panaccione R, Fong A, Chen L, Szyszkowicz M, et al. Effect of ambient air pollution on the incidence of appendicitis. CMAJ 2009;181(9):591-597.
42. Kaplan GG, Szyszkowicz M, Fichna J, Rowe BH, Porada E, Vincent R, et al. Non-specific abdominal pain and air pollution: a novel association. PLoS ONE [Electronic Resource] 2012;7(10):e47669.
43. Kioumourtzoglou M, Power MC, Hart JE, Okereke OI, Coull BA, Laden F, et al. The association between air pollution and onset of depression among middle-aged and older women. Am J Epidemiol 2017;185(9):801-809.
44. Kirrane EF, Bowman C, Davis JA, Hoppin JA, Blair A, Chen H, et al. Associations of ozone and PM2.5 concentrations with Parkinson's Disease among participants in the Agricultural Health Study. J Occup Environ Med 2015;57(5):509-517.
45. Kousha T, Rowe BH. Ambient ozone and emergency department visits due to lower respiratory condition. Int J Occup Environ Health 2014;27(1):50-59.
46. Laurent O, Wu J, Li L, Chung J, Bartell S. Investigating the association between birth weight and complementary air pollution metrics: a cohort study. Environmental Health: A Global Access Science Source 2013;12:18.
47. Lavigne E, Yasseen AS3, Stieb DM, Hystad P, van Donkelaar A, Martin RV, et al. Ambient air pollution and adverse birth outcomes: Differences by maternal comorbidities. Environ Res 2016;148:457-466.
48. Legro RS, Sauer MV, Mottla GL, Richter KS, Li X, Dodson WC, et al. Effect of air quality on assisted human reproduction. Hum Reprod 2010;25(5):1317-1324.
49. Lewis TC, Robins TG, Mentz GB, Zhang X, Mukherjee B, Lin X, et al. Air pollution and respiratory symptoms among children with asthma: vulnerability by corticosteroid use and residence area. Sci Total Environ 2013;448:48-55.
50. Li T, Lin G. Examining the role of location-specific associations between ambient air pollutants and adult asthma in the United States. Health Place 2014;25:26-33.
51. Lin S, Liu X, Le LH, Hwang S. Chronic exposure to ambient ozone and asthma hospital admissions among children. Environ Health Perspect 2008;116(12):1725-1730.
52. Liu S, Krewski D, Shi Y, Chen Y, Burnett RT. Association between maternal exposure to ambient air pollutants during pregnancy and fetal growth restriction. J Expo Sci Environ Epidemiol 2007;17(5):426-432.
53. Liu T, Zeng W, Lin H, Rutherford S, Xiao J, Li X, et al. Tempo-spatial variations of ambient ozone-mortality associations in the USA: Results from the NMMAPS data. Int J Environ Res Public Health [Electronic Resource] 2016;13(9):08 26.
54. Madrigano J, Jack D, Anderson GB, Bell ML, Kinney PL. Temperature, ozone, and mortality in urban and non-urban counties in the northeastern United States. Environmental Health: A Global Access Science Source 2015;14:3.
55. Magzamen S, Moore BF, Yost MG, Fenske RA, Karr CJ. Ozone-related respiratory morbidity in a low-pollution region. J Occup Environ Med 2017;59(7):624-630.
56. Malig BJ, Pearson DL, Chang YB, Broadwin R, Basu R, Green RS, et al. A time-stratified case-crossover study of ambient ozone exposure and emergency department visits for specific respiratory diagnoses in California (2005-2008). Environ Health Perspect 2016;124(6):745-753.
57. Mar TF, Koenig JQ. Relationship between visits to emergency departments for asthma and ozone exposure in greater Seattle, Washington. Ann Allergy Asthma Immunol 2009;103(6):474-479.
58. Medina-Ramon M, Schwartz J. Who is more vulnerable to die from ozone air pollution?. Epidemiology 2008;19(5):672-679.
59. Mendola P, Wallace M, Hwang BS, Liu D, Robledo C, Mannisto T, et al. Preterm birth and air pollution: Critical windows of exposure for women with asthma. J Allergy Clin Immunol 2016;138(2):432-440.e5.
60. Meng Y, Wilhelm M, Rull RP, English P, Ritz B. Traffic and outdoor air pollution levels near residences and poorly controlled asthma in adults. Ann Allergy Asthma Immunol 2007;98(5):455-463.
61. Mobasher Z, Salam MT, Goodwin TM, Lurmann F, Ingles SA, Wilson ML. Associations between ambient air pollution and Hypertensive Disorders of Pregnancy. Environ Res 2013;123:9-16.
62. Montresor-Lopez JA, Yanosky JD, Mittleman MA, Sapkota A, He X, Hibbert JD, et al. Short-term exposure to ambient ozone and stroke hospital admission: A case-crossover analysis. J Expo Sci Environ Epidemiol 2016;26(2):162-166.
63. Morello-Frosch R, Jesdale BM, Sadd JL, Pastor M. Ambient air pollution exposure and full-term birth weight in California. Environmental Health: A Global Access Science Source 2010;9:44.
64. O'Lenick CR, Chang HH, Kramer MR, Winquist A, Mulholland JA, Friberg MD, et al. Ozone and childhood respiratory disease in three US cities: evaluation of effect measure modification by neighborhood socioeconomic status using a Bayesian hierarchical approach. Environmental Health: A Global Access Science Source 2017;16(1):36.
65. O'Lenick CR, Winquist A, Mulholland JA, Friberg MD, Chang HH, Kramer MR, et al. Assessment of neighbourhood-level socioeconomic status as a modifier of air pollution-asthma associations among children in Atlanta. J Epidemiol Community Health 2017;71(2):129-136.
66. Parker JD, Akinbami LJ, Woodruff TJ. Air pollution and childhood respiratory allergies in the United States. Environ Health Perspect 2009;117(1):140-147.
67. Paulu C, Smith AE. Tracking associations between ambient ozone and asthma-related emergency department visits using case-crossover analysis. J Public Health Manag Pract 2008;14(6):581-591.
68. Peel JL, Metzger KB, Klein M, Flanders WD, Mulholland JA, Tolbert PE. Ambient air pollution and cardiovascular emergency department visits in potentially sensitive groups. Am J Epidemiol 2007;165(6):625-633.
69. Peng RD, Samoli E, Pham L, Dominici F, Touloumi G, Ramsay T, et al. Acute effects of ambient ozone on mortality in Europe and North America: results from the APHENA study. Air Qual Atmos Health 2013;6(2):445-453.
70. Pride KR, Peel JL, Robinson BF, Busacker A, Grandpre J, Bisgard KM, et al. Association of short-term exposure to ground-level ozone and respiratory outpatient clinic visits in a rural location - Sublette County, Wyoming, 2008-2011. Environ Res 2015;137:1-7.
71. Raun LH, Ensor KB, Persse D. Using community level strategies to reduce asthma attacks triggered by outdoor air pollution: a case crossover analysis. Environmental Health: A Global Access Science Source 2014;13:58.
72. Ren C, Williams GM, Mengersen K, Morawska L, Tong S. Temperature enhanced effects of ozone on cardiovascular mortality in 95 large US communities, 1987-2000: Assessment using the NMMAPS data. Arch Environ Occup Health 2009;64(3):177-184.
73. Ren C, Williams GM, Mengersen K, Morawska L, Tong S. Does temperature modify short-term effects of ozone on total mortality in 60 large eastern US communities? An assessment using the NMMAPS data. Environ Int 2008;34(4):451-458.
74. Rice MB, Ljungman PL, Wilker EH, Gold DR, Schwartz JD, Koutrakis P, et al. Short-term exposure to air pollution and lung function in the Framingham Heart Study. Am J Respir Crit Care Med 2013;188(11):1351-1357.
75. Roberts JD, Voss JD, Knight B. The association of ambient air pollution and physical inactivity in the United States. PLoS ONE [Electronic Resource] 2014;9(3):e90143.
76. Robledo CA, Mendola P, Yeung E, Mannisto T, Sundaram R, Liu D, et al. Preconception and early pregnancy air pollution exposures and risk of gestational diabetes mellitus. Environ Res 2015;137:316-322.
77. Rodopoulou S, Chalbot M, Samoli E, Dubois DW, San Filippo BD, Kavouras IG. Air pollution and hospital emergency room and admissions for cardiovascular and respiratory diseases in Dona Ana County, New Mexico. Environ Res 2014;129:39-46.
78. Rodopoulou S, Samoli E, Chalbot MG, Kavouras IG. Air pollution and cardiovascular and respiratory emergency visits in Central Arkansas: A time-series analysis. Sci Total Environ 2015;536:872-879.
79. Rojas-Martinez R, Perez-Padilla R, Olaiz-Fernandez G, Mendoza-Alvarado L, Moreno-Macias H, Fortoul T, et al. Lung function growth in children with long-term exposure to air pollutants in Mexico City. Am J Respir Crit Care Med 2007;176(4):377-384.
80. Romieu I, Barraza-Villarreal A, Escamilla-Nunez C, Texcalac-Sangrador JL, Hernandez-Cadena L, Diaz-Sanchez D, et al. Dietary intake, lung function and airway inflammation in Mexico City school children exposed to air pollutants. Respir Res 2009;10:122.
81. Sacks JD, Rappold AG, Davis JAJ, Richardson DB, Waller AE, Luben TJ. Influence of urbanicity and county characteristics on the association between ozone and asthma emergency department visits in North Carolina. Environ Health Perspect 2014;122(5):506-512.
82. Sheffield PE, Zhou J, Shmool JLC, Clougherty JE. Ambient ozone exposure and children's acute asthma in New York City: a case-crossover analysis. Environmental Health: A Global Access Science Source 2015;14:25.
83. Shmool JLC, Kinnee E, Sheffield PE, Clougherty JE. Spatio-temporal ozone variation in a case-crossover analysis of childhood asthma hospital visits in New York City. Environ Res 2016;147:108-114.
84. Silverman RA, Ito K. Age-related association of fine particles and ozone with severe acute asthma in New York City. J Allergy Clin Immunol 2010;125(2):367-373.e5.
85. Smith RL, Xu B, Switzer P. Reassessing the relationship between ozone and short-term mortality in U.S. urban communities. Inhal Toxicol 2009;21(Suppl 2):37-61.
86. Smith ND, Prasad SM, Patel AR, Weiner AB, Pariser JJ, Razmaria A, et al. Bladder cancer mortality in the United States: A geographic and temporal analysis of socioeconomic and environmental factors. J Urol 2016;195(2):290-296.
87. Stieb DM, Szyszkowicz M, Rowe BH, Leech JA. Air pollution and emergency department visits for cardiac and respiratory conditions: a multi-city time-series analysis. Environmental Health: A Global Access Science Source 2009;8:25.
88. Strickland MJ, Klein M, Flanders WD, Chang HH, Mulholland JA, Tolbert PE, et al. Modification of the effect of ambient air pollution on pediatric asthma emergency visits: susceptible subpopulations. Epidemiology 2014;25(6):843-850.
89. Szyszkowicz M, Shutt R, Kousha T, Rowe BH. Air pollution and emergency department visits for epistaxis. Clin Otolaryngol 2014;39(6):345-351.
90. Szyszkowicz M. Ambient air pollution and daily emergency department visits for asthma in Edmonton, Canada. Int J Occup Med Environ Health 2008;21(1):25-30.
91. Szyszkowicz M. Air pollution and emergency department visits for depression in Edmonton, Canada. Int J Occup Med Environ Health 2007;20(3):241-245.
92. Szyszkowicz M, Kousha T, Castner J. Air pollution and emergency department visits for conjunctivitis: A case-crossover study. Int J Occup Med Environ Health 2016;29(3):381-393.
93. Szyszkowicz M, Kousha T, Castner J, Dales R. Air pollution and emergency department visits for respiratory diseases: A multi-city case crossover study. Environ Res 2018;163:263-269.
94. Szyszkowicz M, Kousha T, Kingsbury M, Colman I. Air Pollution and emergency department visits for depression: A multicity case-crossover study. Environ Health Insights 2016;10:155-161.
95. Szyszkowicz M, Porada E, Kaplan GG, Rowe BH. Ambient ozone and emergency department visits for cellulitis. Int J Environ Res Public Health [Electronic Resource] 2010;7(11):4078-4088.
96. Szyszkowicz M, Porada E, Searles G, Rowe BH. Ambient ozone and emergency department visits for skin conditions. Air Qual Atmos Health 2012;5(3):303-309.
97. Thaller EI, Petronella SA, Hochman D, Howard S, Chhikara RS, Brooks EG. Moderate increases in ambient PM2.5 and ozone are associated with lung function decreases in beach lifeguards. J Occup Environ Med 2008;50(2):202-211.
98. To T, Feldman L, Simatovic J, Gershon AS, Dell S, Su J, et al. Health risk of air pollution on people living with major chronic diseases: a Canadian population-based study. BMJ Open 2015;5(9):e009075.
99. Tu J, Tu W, Tedders SH. Spatial variations in the associations of term birth weight with ambient air pollution in Georgia, USA. Environ Int 2016;92-93:146-156.
100. Turner MC, Jerrett M, Pope CA3, Krewski D, Gapstur SM, Diver WR, et al. Long-term ozone exposure and mortality in a large prospective study. Am J Respir Crit Care Med 2016;193(10):1134-1142.
101. Vanos JK, Cakmak S, Kalkstein LS. Association of weather and air pollution interactions on daily mortality in 12 Canadian cities. Air Pollution 2013;174:15-26.
102. Vanos JK, Cakmak S, Bristow C, Brion V, Tremblay N, Martin SL, et al. Synoptic weather typing applied to air pollution mortality among the elderly in 10 Canadian cities. Environ Res 2013;126:66-75.
103. Vanos JK, Hebbern C, Cakmak S. Risk assessment for cardiovascular and respiratory mortality due to air pollution and synoptic meteorology in 10 Canadian cities. Environmental Pollution 2014;185:322-332.
104. Villeneuve PJ, Chen L, Rowe BH, Coates F. Outdoor air pollution and emergency department visits for asthma among children and adults: a case-crossover study in northern Alberta, Canada. Environmental Health: A Global Access Science Source 2007;6:40.
105. Vinikoor-Imler LC, Davis JA, Meyer RE, Messer LC, Luben TJ. Associations between prenatal exposure to air pollution, small for gestational age, and term low birthweight in a state-wide birth cohort. Environ Res 2014;132:132-139.
106. Wang X, Kindzierski W, Kaul P. Air pollution and acute myocardial infarction hospital admission in Alberta, Canada: A three-step procedure case-crossover study. PLoS ONE [Electronic Resource] 2015;10(7):e0132769.
107. Ware LB, Zhao Z, Koyama T, May AK, Matthay MA, Lurmann FW, et al. Long-term ozone exposure increases the risk of developing the acute respiratory distress syndrome. Am J Respir Crit Care Med 2016;193(10):1143-1150.
108. Warren J, Fuentes M, Herring A, Langlois P. Spatial-temporal modeling of the association between air pollution exposure and preterm birth: identifying critical windows of exposure. Biometrics 2012;68(4):1157-1167.
109. Wendt JK, Symanski E, Stock TH, Chan W, Du XL. Association of short-term increases in ambient air pollution and timing of initial asthma diagnosis among Medicaid-enrolled children in a metropolitan area. Environ Res 2014;131:50-58.
110. Wing JJ, Adar SD, Sanchez BN, Morgenstern LB, Smith MA, Lisabeth LD. Ethnic differences in ambient air pollution and risk of acute ischemic stroke. Environ Res 2015;143(Pt A):62-67.
111. Wing JJ, Sanchez BN, Adar SD, Meurer WJ, Morgenstern LB, Smith MA, et al. Synergism of short-term air pollution exposures and neighborhood disadvantage on initial stroke severity. Stroke 2017;48(11):3126-3129.
112. Winquist A, Klein M, Tolbert P, Flanders WD, Hess J, Sarnat SE. Comparison of emergency department and hospital admissions data for air pollution time-series studies. Environmental Health: A Global Access Science Source 2012;11:70.
113. Wu J, Laurent O, Li L, Hu J, Kleeman M. Adverse reproductive health outcomes and exposure to gaseous and particulate-matter air pollution in pregnant women. Res Rep Health Eff Inst 2016.
114. Zanobetti A, Schwartz J. Ozone and survival in four cohorts with potentially predisposing diseases. Am J Respir Crit Care Med 2011;184(7):836-841.
115. Zanobetti A, Schwartz J. Is there adaptation in the ozone mortality relationship: a multi-city case-crossover analysis. Environmental Health: A Global Access Science Source 2008;7:22.
116. Zemek R, Szyszkowicz M, Rowe BH. Air pollution and emergency department visits for otitis media: a case-crossover study in Edmonton, Canada. Environ Health Perspect 2010;118(11):1631-1636.
117. Zu K, Liu X, Shi L, Tao G, Loftus CT, Lange S, et al. Concentration-response of short-term ozone exposure and hospital admissions for asthma in Texas. Environ Int 2017;104:139-145.
