# Supplementary material for: Geospatial indicators of exposure, sensitivity, and adaptive capacity to assess neighbourhood variation in vulnerability to climate change-related health hazards
Source: Environ Health. 2021 Mar 22;20:31. doi: 10.1186/s12940-021-00708-z (PMC7986027; doi:10.1186/s12940-021-00708-z)
Supplement: Supplementary file 2 — Additional file 2. [file 12940_2021_708_MOESM2_ESM.docx]

**Additional file 2– Results of flood guidelines search**

An online search was done to investigate the availability of flood guidelines, such as official community plans and hazard risk and vulnerability assessments, were publicly available for the following census divisions (regional districts) and census subdivisions (municipalities). A broad search criterion was used (‘municipality’ and ‘flood planning guidelines’) using Google search engine and [Resilient Coasts Canada](https://resilient-c.ubc.ca/) database.

| **Census divisions (regional districts)** | **Census subdivisions (municipalities)** | **Yes (1) / No (0)** |
| --- | --- | --- |
| Fraser Valley | Abbotsford | 1 |
| Fraser Valley | Aitchelitch 9 | 0 |
| Fraser Valley | Albert Flat 5 | 0 |
| Greater Vancouver | Anmore | 1 |
| Greater Vancouver | Barnston Island 3 | 1 |
| Greater Vancouver | Belcarra | 1 |
| Central Coast | Bella Bella 1 | 1 |
| Central Coast | Bella Coola 1 | 1 |
| Cariboo | Betty Creek 18 | 0 |
| Cariboo | Blackwater Meadow 11 | 1 |
| Fraser Valley | Boston Bar 1A | 0 |
| Greater Vancouver | Bowen Island | 1 |
| Fraser Valley | Bucktum 4 | 0 |
| Bulkley-Nechako | Bulkley-Nechako E | 1 |
| Greater Vancouver | Burnaby | 1 |
| Greater Vancouver | Burrard Inlet 3 | 1 |
| Cariboo | Cahoose 10 | 0 |
| Cariboo | Cahoose 12 | 0 |
| Greater Vancouver | Capilano 5 | 1 |
| Cariboo | Cariboo J | 1 |
| Central Coast | Central Coast A | 0 |
| Central Coast | Central Coast C | 0 |
| Central Coast | Central Coast D | 0 |
| Central Coast | Central Coast E | 0 |
| Fraser Valley | Chawathil 4 | 1 |
| Squamish-Lillooet | Cheakamus 11 | 1 |
| Fraser Valley | Cheam 1 | 0 |
| Fraser Valley | Chehalis 5 | 1 |
| Sunshine Coast | Chekwelp 26 | 0 |
| Fraser Valley | Chilliwack | 1 |
| Greater Vancouver | Coquitlam | 1 |
| Greater Vancouver | Coquitlam 1 | 1 |
| Greater Vancouver | Coquitlam 2 | 1 |
| Greater Vancouver | Delta | 1 |
| Fraser Valley | Douglas 8 | 0 |
| Cariboo | Fishtrap 19 | 0 |
| Fraser Valley | Fraser Valley A | 1 |
| Fraser Valley | Fraser Valley B | 1 |
| Fraser Valley | Fraser Valley C | 1 |
| Fraser Valley | Fraser Valley D | 1 |
| Fraser Valley | Fraser Valley E | 1 |
| Fraser Valley | Fraser Valley F | 1 |
| Fraser Valley | Fraser Valley G | 1 |
| Fraser Valley | Fraser Valley H | 1 |
| Sunshine Coast | Gibsons | 1 |
| Greater Vancouver | Greater Vancouver A | 1 |
| Fraser Valley | Harrison Hot Springs | 1 |
| Fraser Valley | Holachten 8 | 1 |
| Fraser Valley | Hope | 1 |
| Central Coast | Katit 1 | 0 |
| Greater Vancouver | Katzie 1 | 1 |
| Greater Vancouver | Katzie 2 | 1 |
| Fraser Valley | Kent | 1 |
| Kitimat-Stikine | Kitasoo 1 | 0 |
| Kitimat-Stikine | Kitimat-Stikine C (Part 2) | 1 |
| Fraser Valley | Kopchitchin 2 | 0 |
| Squamish-Lillooet | Kowtain 17 | 1 |
| Fraser Valley | Kwawkwawapilt 6 | 0 |
| Fraser Valley | Lakahahmen 11 | 0 |
| Greater Vancouver | Langley | 1 |
| Greater Vancouver | Langley 5 | 1 |
| Greater Vancouver | Lions Bay | 1 |
| Cariboo | Louis Squinas Ranch 14 | 0 |
| Fraser Valley | Lukseetsissum 9 | 0 |
| Greater Vancouver | Maple Ridge | 1 |
| Fraser Valley | Matsqui Main 2 | 1 |
| Greater Vancouver | McMillan Island 6 | 1 |
| Fraser Valley | Mission | 1 |
| Greater Vancouver | Mission 1 | 1 |
| Squamish-Lillooet | Mount Currie | 0 |
| Mount Waddington | Mount Waddington A | 1 |
| Greater Vancouver | Musqueam 2 | 0 |
| Greater Vancouver | Musqueam 4 | 0 |
| Squamish-Lillooet | Nequatque | 0 |
| Squamish-Lillooet | Nesuch 3 | 0 |
| Greater Vancouver | New Westminster | 1 |
| Greater Vancouver | North Vancouver | 1 |
| Fraser Valley | Ohamil 1 | 0 |
| Okanagan-Similkameen | Okanagan-Similkameen H | 1 |
| Fraser Valley | Paqulh | 0 |
| Squamish-Lillooet | Pemberton | 1 |
| Fraser Valley | Peters 1 | 0 |
| Greater Vancouver | Pitt Meadows | 1 |
| Fraser Valley | Popkum 1 | 1 |
| Greater Vancouver | Port Coquitlam | 1 |
| Greater Vancouver | Port Moody | 1 |
| Powell River | Powell River | 1 |
| Powell River | Powell River A | 1 |
| Powell River | Powell River B | 1 |
| Powell River | Powell River C | 1 |
| Powell River | Powell River D | 1 |
| Fraser Valley | Puckatholetchin 11 | 0 |
| Fraser Valley | Q'alatkú7em | 0 |
| Greater Vancouver | Richmond | 1 |
| Fraser Valley | Sachteen | 0 |
| Fraser Valley | Saddle Rock 9 | 0 |
| Cariboo | Salmon River Meadow 7 | 0 |
| Fraser Valley | Schelowat 1 | 0 |
| Fraser Valley | Schkam 2 | 0 |
| Fraser Valley | Scowlitz 1 | 0 |
| Fraser Valley | Seabird Island | 1 |
| Squamish-Lillooet | Seaichem 16 | 0 |
| Sunshine Coast | Sechelt | 1 |
| Powell River | Sechelt (Part) | 1 |
| Sunshine Coast | Sechelt (Part) | 1 |
| Greater Vancouver | Semiahmoo | 0 |
| Greater Vancouver | Seymour Creek 2 | 1 |
| Fraser Valley | Skawahlook 1 | 1 |
| Fraser Valley | Skookumchuck 4 | 1 |
| Fraser Valley | Skowkale | 1 |
| Fraser Valley | Skwah 4 | 0 |
| Fraser Valley | Skwahla 2 | 0 |
| Fraser Valley | Skwali 3 | 0 |
| Fraser Valley | Skway 5 | 0 |
| Fraser Valley | Skweahm 10 | 0 |
| Powell River | Sliammon 1 | 1 |
| Fraser Valley | Soowahlie 14 | 0 |
| Fraser Valley | Spuzzum 1 | 1 |
| Squamish-Lillooet | Squamish | 1 |
| Squamish-Lillooet | Squamish-Lillooet C | 1 |
| Squamish-Lillooet | Squamish-Lillooet D | 1 |
| Fraser Valley | Squawkum Creek 3 | 0 |
| Fraser Valley | Squiaala | 0 |
| Cariboo | Squinas 2 | 0 |
| Squamish-Lillooet | Stawamus 24 | 0 |
| Strathcona | Strathcona B | 1 |
| Strathcona | Strathcona C | 1 |
| Fraser Valley | Stullawheets 8 | 1 |
| Sunshine Coast | Sunshine Coast A | 1 |
| Sunshine Coast | Sunshine Coast B | 1 |
| Sunshine Coast | Sunshine Coast D | 1 |
| Sunshine Coast | Sunshine Coast E | 1 |
| Sunshine Coast | Sunshine Coast F | 1 |
| Greater Vancouver | Surrey | 1 |
| Cariboo | Thomas Squinas Ranch 2A | 0 |
| Thompson-Nicola | Thompson-Nicola I (Blue Sky Country) | 1 |
| Thompson-Nicola | Thompson-Nicola N (Beautiful Nicola Valley - South) | 1 |
| Fraser Valley | Tipella 7 | 0 |
| Cariboo | Towdystan Lake 3 | 0 |
| Greater Vancouver | Tsawwassen | 1 |
| Fraser Valley | Tseatah 2 | 0 |
| Fraser Valley | Tuckkwiowhum 1 | 0 |
| Fraser Valley | Tzeachten 13 | 1 |
| Cariboo | Ulkatcho 13 | 0 |
| Cariboo | Ulkatcho 14A | 0 |
| Fraser Valley | Upper Sumas 6 | 1 |
| Greater Vancouver | Vancouver | 1 |
| Squamish-Lillooet | Waiwakum 14 | 0 |
| Greater Vancouver | West Vancouver | 1 |
| Squamish-Lillooet | Whistler | 1 |
| Greater Vancouver | White Rock | 1 |
| Greater Vancouver | Whonnock 1 | 0 |
| Fraser Valley | Yakweakwioose 12 | 0 |
| Fraser Valley | Yale Town 1 | 0 |
| Squamish-Lillooet | Yekwaupsum 18 | 0 |
