# Supplementary material for: Geospatial indicators of exposure, sensitivity, and adaptive capacity to assess neighbourhood variation in vulnerability to climate change-related health hazards
Source: Environ Health. 2021 Mar 22;20:31. doi: 10.1186/s12940-021-00708-z (PMC7986027; doi:10.1186/s12940-021-00708-z)
Supplement: Supplementary file 3 — Additional file 3. [file 12940_2021_708_MOESM3_ESM.docx]

**Additional file 3 – Indicator decisions and data sources**

Table 4a – Extreme Heat indicator decisions and data source:

**All indicators from the systematic literature review are identified below. Indicators that remained after running the two-step PCA process and where data were available are* ***bolded*** *in the table below. Unless otherwise specified****,*** *the proportion of households in a community (dissemination area) that was recorded to have a given trait of a binary variable was computed.*

| **Categories** | **Determinants** | **Indicators** | **Data source** |
| --- | --- | --- | --- |
| **Exposure** | Climate | **Extreme heat - % of days per year >25 degrees Celsius** | [CANUE (2015)](https://canue.ca/) |
| **Sensitivity** | Age | **<5 years old** | [Statistics Canada - Census (2016)](https://www12.statcan.gc.ca/census-recensement/2016/dp-pd/prof/details/download-telecharger/comp/page_dl-tc.cfm?Lang=E) |
|  |  | **>65 years old** | [Statistics Canada - Census (2016)](https://www12.statcan.gc.ca/census-recensement/2016/dp-pd/prof/details/download-telecharger/comp/page_dl-tc.cfm?Lang=E) |
|  | Pre-existing health conditions | **Cardiovascular - Acute Myocardial Infarction (Crude Rate per 1000)** | [Chronic Disease Registry (2015/16)](http://www.bccdc.ca/health-professionals/data-reports/chronic-disease-dashboard) |
|  |  | **Cardiovascular - Coronary Artery Bypass Graft (Crude Rate per 1000)** | [Chronic Disease Registry (2015/16)](http://www.bccdc.ca/health-professionals/data-reports/chronic-disease-dashboard) |
|  |  | **Cardiovascular - Hospitalized Stroke (Crude Rate per 1000)** | [Chronic Disease Registry (2015/16)](http://www.bccdc.ca/health-professionals/data-reports/chronic-disease-dashboard) |
|  |  | **Respiratory - Asthma (Crude Rate per 1000)** | [Chronic Disease Registry (2015/16)](http://www.bccdc.ca/health-professionals/data-reports/chronic-disease-dashboard) |
|  |  | **Renal - Chronic Kidney Disease (Crude Rate per 1000)** | [Chronic Disease Registry (2015/16)](http://www.bccdc.ca/health-professionals/data-reports/chronic-disease-dashboard) |
|  |  | **Mental Health -Episodic Mood Anxiety Disorders (Crude Rate per 1000)** | [Chronic Disease Registry (2015/16)](http://www.bccdc.ca/health-professionals/data-reports/chronic-disease-dashboard) |
|  |  | **Mental Health -Depression (Crude Rate per 1000)** |  |
|  |  | **Cerebrovascular - Hospitalized Stroke (Crude Rate per 1000)** | [Chronic Disease Registry (2015/16)](http://www.bccdc.ca/health-professionals/data-reports/chronic-disease-dashboard) |
|  |  | **Chronic health problems (general) - Multiple chronic conditions** | [My Health, My Community (2014)](https://www.myhealthmycommunity.org/Results/CommunityProfiles.aspx) |
|  |  | Pregnancy (low birth weights, congenital malformations) | Data unavailable |
| **Adaptive Capacity** | Socioeconomic | **Low education -**  **No certificate, diploma or degree** | [Statistics Canada - Census (2016)](https://www12.statcan.gc.ca/census-recensement/2016/dp-pd/prof/details/download-telecharger/comp/page_dl-tc.cfm?Lang=E) |
|  |  | Below poverty – Material and Social Deprivation Index | [CANUE (2011)](https://canue.ca/) |
|  |  | Occupation type (outdoors) | Data unavailable |
|  | Race/ethnicity | **Visible minority groups -**  **Total visible minority population** | [Statistics Canada - Census (2016)](https://www12.statcan.gc.ca/census-recensement/2016/dp-pd/prof/details/download-telecharger/comp/page_dl-tc.cfm?Lang=E) |
|  |  | **Indigenous status -**  **Aboriginal identity** | [Statistics Canada - Census (2016)](https://www12.statcan.gc.ca/census-recensement/2016/dp-pd/prof/details/download-telecharger/comp/page_dl-tc.cfm?Lang=E) |
|  | Built environment | **Poor housing condition -**  **Housing not suitable** | [Statistics Canada - Census (2016)](https://www12.statcan.gc.ca/census-recensement/2016/dp-pd/prof/details/download-telecharger/comp/page_dl-tc.cfm?Lang=E) |
|  |  | **Population density per square kilometer**  (continuous variable) | [Statistics Canada - Census (2016)](https://www12.statcan.gc.ca/census-recensement/2016/dp-pd/prof/details/download-telecharger/comp/page_dl-tc.cfm?Lang=E) |
|  |  | **Impervious surfaces - annual mean normalized difference vegetation index (inverted)**  (continuous variable) | [CANUE (2015)](https://canue.ca/) |
|  |  | No air conditioning | Data unavailable |
|  | Social cohesion | **Living alone -**  **Dwelling characteristics - 1 person** | [Statistics Canada - Census (2016)](https://www12.statcan.gc.ca/census-recensement/2016/dp-pd/prof/details/download-telecharger/comp/page_dl-tc.cfm?Lang=E) |
|  | Institutional | **Heat strategy guidelines (Absence)** | [BCCDC (2017)](http://www.bccdc.ca/resource-gallery/Documents/Guidelines%20and%20Forms/Guidelines%20and%20Manuals/Health-Environment/BC%20Municipal%20Heat%20Response%20Planning.pdf) |
|  |  | Adaptation Index (or level of adaptation) | Data unavailable |

Table 4b – Flooding indicator decisions and data source:

**All indicators from the systematic literature review are identified below. Final indicators that remained after running the two-step PCA process and where data were available are* ***bolded*** *in the table below. Unless otherwise specified****,*** *the proportion of households in a community (dissemination area) that was recorded to have a given trait of a binary variable was computed.*

| **Categories** | **Determinants** | **Indicators** | **Data source** |
| --- | --- | --- | --- |
| **Exposure** | Climate | **Inland flooding and predicted sea-level rise** | [City of Vancouver (2012)](https://vancouver.ca/green-vancouver/sea-level-rise.aspx), [Fraser Basin Council (2014)](https://www.fraserbasin.bc.ca/Phase_1_Results.html) |
| **Sensitivity** | Age | **<5 years old** | [Statistics Canada - Census (2016)](https://www12.statcan.gc.ca/census-recensement/2016/dp-pd/prof/details/download-telecharger/comp/page_dl-tc.cfm?Lang=E) |
|  |  | **>65 years old** | [Statistics Canada - Census (2016)](https://www12.statcan.gc.ca/census-recensement/2016/dp-pd/prof/details/download-telecharger/comp/page_dl-tc.cfm?Lang=E) |
|  | Pre-existing health conditions | **Mental health – Episodic Mood Anxiety Disorders (Crude Rate per 1000)** | [Chronic Disease Registry (2015/16)](http://www.bccdc.ca/health-professionals/data-reports/chronic-disease-dashboard) |
|  |  | **Mental health - Depression (Crude Rate per 1000)** | [Chronic Disease Registry (2015/16)](http://www.bccdc.ca/health-professionals/data-reports/chronic-disease-dashboard) |
|  |  | **Chronic lung disease - Asthma (Crude Rate per 1000)** | [Chronic Disease Registry (2015/16)](http://www.bccdc.ca/health-professionals/data-reports/chronic-disease-dashboard) |
|  |  | **Chronic Obstructive Pulmonary Disease (Crude Rate per 1000)** | [Chronic Disease Registry (2015/16)](http://www.bccdc.ca/health-professionals/data-reports/chronic-disease-dashboard) |
|  |  | **General health (excellent/very good)** | [My Health, My Community (2014)](https://www.myhealthmycommunity.org/Results/CommunityProfiles.aspx) |
|  |  | Physical injuries | Data unavailable |
| **Adaptive Capacity** | Socioeconomic | **Low education -**  **No certificate, diploma or degree** | [Statistics Canada - Census (2016)](https://www12.statcan.gc.ca/census-recensement/2016/dp-pd/prof/details/download-telecharger/comp/page_dl-tc.cfm?Lang=E) |
|  |  | **Low language skills - Neither English- nor French-speaking** | [Statistics Canada - Census (2016)](https://www12.statcan.gc.ca/census-recensement/2016/dp-pd/prof/details/download-telecharger/comp/page_dl-tc.cfm?Lang=E) |
|  |  | **Access to medical services - family doctor access (no)** | [My Health, My Community (2014)](https://www.myhealthmycommunity.org/Results/CommunityProfiles.aspx) |
|  |  | Low income - In low income based on the Low-income cut-offs, after tax | [Statistics Canada - Census (2016)](https://www12.statcan.gc.ca/census-recensement/2016/dp-pd/prof/details/download-telecharger/comp/page_dl-tc.cfm?Lang=E) |
|  | Race/ethnicity | **Visible minority groups -**  **Total visible minority population** | [Statistics Canada - Census (2016)](https://www12.statcan.gc.ca/census-recensement/2016/dp-pd/prof/details/download-telecharger/comp/page_dl-tc.cfm?Lang=E) |
|  |  | **Indigenous status -**  **Aboriginal identity** | [Statistics Canada - Census (2016)](https://www12.statcan.gc.ca/census-recensement/2016/dp-pd/prof/details/download-telecharger/comp/page_dl-tc.cfm?Lang=E) |
|  | Immigration status | **Recent immigrants - Immigrants from 2011 to 2016** | [Statistics Canada - Census (2016)](https://www12.statcan.gc.ca/census-recensement/2016/dp-pd/prof/details/download-telecharger/comp/page_dl-tc.cfm?Lang=E) |
|  |  | Non-citizens - Non-permanent residents | [Statistics Canada - Census (2016)](https://www12.statcan.gc.ca/census-recensement/2016/dp-pd/prof/details/download-telecharger/comp/page_dl-tc.cfm?Lang=E) |
|  | Built environment | **Poor housing -**  **Housing needing major repairs** | [Statistics Canada - Census (2016)](https://www12.statcan.gc.ca/census-recensement/2016/dp-pd/prof/details/download-telecharger/comp/page_dl-tc.cfm?Lang=E) |
|  |  | **Poor access to transit - transit stop (less than 5 min walk)** | [My Health, My Community (2014)](https://www.myhealthmycommunity.org/Results/CommunityProfiles.aspx) |
|  |  | **Population density per square kilometer**  (continuous variable) | [Statistics Canada - Census (2016)](https://www12.statcan.gc.ca/census-recensement/2016/dp-pd/prof/details/download-telecharger/comp/page_dl-tc.cfm?Lang=E) |
|  |  | No air conditioning | Data unavailable |
|  | Social cohesion | **Weak social network – 4+ people to confide in/turn to for help (no)** | [My Health, My Community (2014)](https://www.myhealthmycommunity.org/Results/CommunityProfiles.aspx) |
|  |  | **Living alone -**  **Dwelling characteristics: 1 person** | [Statistics Canada - Census (2016)](https://www12.statcan.gc.ca/census-recensement/2016/dp-pd/prof/details/download-telecharger/comp/page_dl-tc.cfm?Lang=E) |
|  | Institutional | **Flood hazard planning and mitigation guidelines in municipalities** | [Resilient-C](https://resilient-c.ubc.ca/), UBC^1^ |
|  |  | **Evacuation and displacement plans - emergency supplies (3+ days) (no)** | [My Health, My Community (2014)](https://www.myhealthmycommunity.org/Results/CommunityProfiles.aspx) |
|  |  | Population awareness levels | Data unavailable |
|  |  | Hospital and shelter response capacity | Data unavailable |

^1^ For municipalities where guidelines were not available from the Resilience-C platform, a Google search was conducted for the (municipality) and ‘flood planning guidelines’ to assess for official community plans, hazard risk and vulnerability assessment, and any major study done mentioned in the former documents. The variable was categorized as 1 (yes) and 0 (no). The results can be found in **Additional file 3.**

Table 4c – Wildfire smoke indicator decisions and data source:

**All indicators from the systematic literature review are identified below. Final indicators that remained after running the two-step PCA process and where data were available are* ***bolded*** *in the table below. Unless otherwise specified****,*** *the proportion of households in a community (dissemination area) that was recorded to have a given trait of a binary variable was computed.*

| **Categories** | **Determinants** | **Indicators** | **Data source** |
| --- | --- | --- | --- |
| **Exposure** | Climate | **Extreme smoke impact - Percentage of days when daily average PM2.5 concentration is >= 25ug/m3 (microgram per cubic meter), among all days (905 days in total) during the five intense fire seasons in the last ten years (April 1 to September 30 in year 2009, 2010, 2014, 2015 and 2017)**  (continuous variable) | [Optimized Statistical Smoke Exposure Model](https://www.nature.com/articles/jes201387) (99) |
| **Sensitivity** | Age | **<5 years old** | [Statistics Canada - Census (2016)](https://www12.statcan.gc.ca/census-recensement/2016/dp-pd/prof/details/download-telecharger/comp/page_dl-tc.cfm?Lang=E) |
|  |  | **>65 years old** | [Statistics Canada - Census (2016)](https://www12.statcan.gc.ca/census-recensement/2016/dp-pd/prof/details/download-telecharger/comp/page_dl-tc.cfm?Lang=E) |
|  | Pre-existing health conditions | **Existing respiratory disease - Asthma (Crude Rate per 1000)** | [Chronic Disease Registry (2015/16)](http://www.bccdc.ca/health-professionals/data-reports/chronic-disease-dashboard) |
|  |  | **Existing respiratory disease - Chronic Obstructive Pulmonary Disease (Crude Rate per 1000)** | [Chronic Disease Registry (2015/16)](http://www.bccdc.ca/health-professionals/data-reports/chronic-disease-dashboard) |
|  |  | **Existing cardiovascular disease - Acute Myocardial Infarction (Crude Rate per 1000)** | [Chronic Disease Registry (2015/16)](http://www.bccdc.ca/health-professionals/data-reports/chronic-disease-dashboard) |
|  |  | **Existing cardiovascular disease - Coronary Artery Bypass Graft (Crude Rate per 1000)** | [Chronic Disease Registry (2015/16)](http://www.bccdc.ca/health-professionals/data-reports/chronic-disease-dashboard) |
|  |  | **Existing cardiovascular disease - Hospitalized Stroke (Crude Rate per 1000)** | [Chronic Disease Registry (2015/16)](http://www.bccdc.ca/health-professionals/data-reports/chronic-disease-dashboard) |
|  |  | **Existing cardiovascular disease - Hypertension (Crude Rate per 1000)** | [Chronic Disease Registry (2015/16)](http://www.bccdc.ca/health-professionals/data-reports/chronic-disease-dashboard) |
|  |  | **Chronic health problems (general)** | [My Health, My Community (2014)](https://www.myhealthmycommunity.org/Results/CommunityProfiles.aspx) |
|  |  | Pregnancy | Data unavailable |
| **Adaptive capacity** | Socioeconomic | **Low education - No certificate, diploma or degree** | [Statistics Canada - Census (2016)](https://www12.statcan.gc.ca/census-recensement/2016/dp-pd/prof/details/download-telecharger/comp/page_dl-tc.cfm?Lang=E) |
|  |  | **Income inequality – Material Deprivation Index**  (continuous variable) | [CANUE (2011)](https://canue.ca/) |
|  |  | Low income - In low income based on the Low-income cut-offs, after tax | [Statistics Canada - Census (2016)](https://www12.statcan.gc.ca/census-recensement/2016/dp-pd/prof/details/download-telecharger/comp/page_dl-tc.cfm?Lang=E) |
|  | Sex | **Females** | [Statistics Canada - Census (2016)](https://www12.statcan.gc.ca/census-recensement/2016/dp-pd/prof/details/download-telecharger/comp/page_dl-tc.cfm?Lang=E) |
|  | Race/ethnicity | **Visible minority groups -Total visible minority population** | [Statistics Canada - Census (2016)](https://www12.statcan.gc.ca/census-recensement/2016/dp-pd/prof/details/download-telecharger/comp/page_dl-tc.cfm?Lang=E) |
|  |  | **Indigenous status -**  **Aboriginal identity** | [Statistics Canada - Census (2016)](https://www12.statcan.gc.ca/census-recensement/2016/dp-pd/prof/details/download-telecharger/comp/page_dl-tc.cfm?Lang=E) |
|  | Immigration status | **Recent immigrants - Immigrants from 2011 to 2016** | [Statistics Canada - Census (2016)](https://www12.statcan.gc.ca/census-recensement/2016/dp-pd/prof/details/download-telecharger/comp/page_dl-tc.cfm?Lang=E) |
|  | Built environment | Air conditioning | Data unavailable |
|  | Social cohesion | **Weak social network - Community belonging (not strong/not somewhat strong)** | [My Health, My Community (2014)](https://www.myhealthmycommunity.org/Results/CommunityProfiles.aspx) |
|  | Institutional | Guidelines | Data unavailable |

*Principal component analysis*

*Wildfire smoke sensitivity index*

**Figure I.1** of **Appendix I** presents the results of the principal component analysis for the sensitivity category for wildfire smoke. **Three** components were retained that met the criterion. Variables that loaded highly within each component include: **component 1 [respiratory diseases** (asthma and chronic obstructive pulmonary disease) **and cardiovascular diseases** (acute myocardial infarction, coronary artery bypass graft, hypertension, stroke**], component 2 [age** (<5 and >65 years old)**], and component 3 (general self-rated health).** The components explained 47.1%, 14.6%, and 11.5% of the total variance respectively. Collectively, the **three** components explained **73.2%** of the total variance of the wildfire smoke sensitivity index across the region.

*Wildfire smoke adaptive capacity index*

**Figure I.2** of **Appendix I** presents the results of the principal component analysis for the adaptive capacity category for wildfire smoke. **Three** components were retained based on the criterion**.** The variables that loaded highly within each component can be summarised as**: component 1 (recent immigrant, visible minority, female), component 2 (indigenous status and low education), and component 3 (community belonging and income inequality).** The components explained 38.8%, 21.3%, and 15.9% of the total variance respectively. Collectively, the three components explained **75.9%** of the total variance of the wildfire smoke adaptive capacity index across the region.

Overall wildfire smoke vulnerability index

*Overall wildfire smoke index*

**Figure I.3** of **Appendix I** presents the results of the contributions of the different categories (derived by percentage of variance) to the overall wildfire smoke vulnerability index scores after incorporating the exposure scores into a PCA along with the sensitivity and adaptive capacity scores. The final PCA results determined the corresponding weights that were used in the final wildfire smoke vulnerability index scores: **sensitivity (39.9%), exposure (34.1%), and adaptive capacity (26.0%)**.

Table 4d – Ground-level ozone indicator decisions and data source:

**Indicators that remained after running the two-step PCA process and where data were available are* ***bolded*** *in the table below. Final indicators that remained after running the two-step PCA process and where data were available are* ***bolded*** *in the table below. Unless otherwise specified****,*** *the proportion of households in a community (dissemination area) that was recorded to have a given trait of a binary variable was computed.*

| **Categories** | **Determinants** | **Indicators** | **Data source** |
| --- | --- | --- | --- |
| **Exposure** | Climate | **Ground-level Ozone - annual modelled concentration estimate**  (continuous variable) | [CANUE (2015)](https://canue.ca/) |
| **Sensitivity** | Age | **<19 years old -  **Added across 0 to 4, 5 to 9, 10 to 14, 15 to 19 groups from census*** | [Statistics Canada - Census (2016)](https://www12.statcan.gc.ca/census-recensement/2016/dp-pd/prof/details/download-telecharger/comp/page_dl-tc.cfm?Lang=E) |
|  |  | **>65 years old** | [Statistics Canada - Census (2016)](https://www12.statcan.gc.ca/census-recensement/2016/dp-pd/prof/details/download-telecharger/comp/page_dl-tc.cfm?Lang=E) |
|  | Pre-existing health conditions | **Existing respiratory disease -**  **Chronic Obstructive Pulmonary Disease (Crude Rate per 1000)** | [Chronic Disease Registry (2015/16)](http://www.bccdc.ca/health-professionals/data-reports/chronic-disease-dashboard) |
|  |  | **Existing cardiovascular disease - Hypertension (Crude Rate per 1000)** | [Chronic Disease Registry (2015/16)](http://www.bccdc.ca/health-professionals/data-reports/chronic-disease-dashboard) |
|  |  | **General health (excellent/very good)** | [My Health, My Community (2014)](https://www.myhealthmycommunity.org/Results/CommunityProfiles.aspx) |
|  |  | Diabetes | Data unavailable |
|  |  | Obesity | Data unavailable |
|  |  | 2^nd^ trimester of pregnancy | Data unavailable |
| **Adaptive Capacity** | Socioeconomic | **Poverty - Material and Social Deprivation Index**  (continuous variable) | [CANUE (2011)](https://canue.ca/) |
|  |  | Low income based on the low-income cut-offs, after tax | [Statistics Canada - Census (2016)](https://www12.statcan.gc.ca/census-recensement/2016/dp-pd/prof/details/download-telecharger/comp/page_dl-tc.cfm?Lang=E) |
|  |  | Population aged 15 years and over by labour force status - unemployed | [CANUE (2011)](https://canue.ca/) |
|  |  | Outdoor work | Data unavailable |
|  | Race/ethnicity | **Visible minority groups - Total visible minority population** | [Statistics Canada - Census (2016)](https://www12.statcan.gc.ca/census-recensement/2016/dp-pd/prof/details/download-telecharger/comp/page_dl-tc.cfm?Lang=E) |
|  |  | **Indigenous status -**  **Aboriginal identity** | [Statistics Canada - Census (2016)](https://www12.statcan.gc.ca/census-recensement/2016/dp-pd/prof/details/download-telecharger/comp/page_dl-tc.cfm?Lang=E) |
|  | Immigration status | **Immigrant status** | [Statistics Canada - Census (2016)](https://www12.statcan.gc.ca/census-recensement/2016/dp-pd/prof/details/download-telecharger/comp/page_dl-tc.cfm?Lang=E) |
|  | Built environment | **Nonurban - <400 people per square kilometre (derived from population density data)** | [Statistics Canada - Census (2016)](https://www12.statcan.gc.ca/census-recensement/2016/dp-pd/prof/details/download-telecharger/comp/page_dl-tc.cfm?Lang=E) |
|  |  | **Public transit use** | [Statistics Canada - Census (2016)](https://www12.statcan.gc.ca/census-recensement/2016/dp-pd/prof/details/download-telecharger/comp/page_dl-tc.cfm?Lang=E) |
|  |  | **Low fruit and vegetable intake - 5+ servings of fruits and vegetables (no)** | [My Health, My Community (2014)](https://www.myhealthmycommunity.org/Results/CommunityProfiles.aspx) |
|  |  | No central AC | Data unavailable |
|  |  | Outside air infiltration | Data unavailable |
