# Supplementary material for: Geospatial indicators of exposure, sensitivity, and adaptive capacity to assess neighbourhood variation in vulnerability to climate change-related health hazards
Source: Environ Health. 2021 Mar 22;20:31. doi: 10.1186/s12940-021-00708-z (PMC7986027; doi:10.1186/s12940-021-00708-z)
Supplement: Supplementary file 4 — Additional file 4. [file 12940_2021_708_MOESM4_ESM.docx]

**Additional 4 – Summary of data reduction process for extreme heat, wildfire smoke, and ground-level ozone**

Extreme heat:

Wildfire smoke:

Ground-level ozone
