# Supplementary material for: Geospatial indicators of exposure, sensitivity, and adaptive capacity to assess neighbourhood variation in vulnerability to climate change-related health hazards
Source: Environ Health. 2021 Mar 22;20:31. doi: 10.1186/s12940-021-00708-z (PMC7986027; doi:10.1186/s12940-021-00708-z)
Supplement: Supplementary file 5 — Additional file 5. [file 12940_2021_708_MOESM5_ESM.docx]

**Additional file 5 - Category-specific and overall vulnerability index maps**

**Extreme Heat**

**
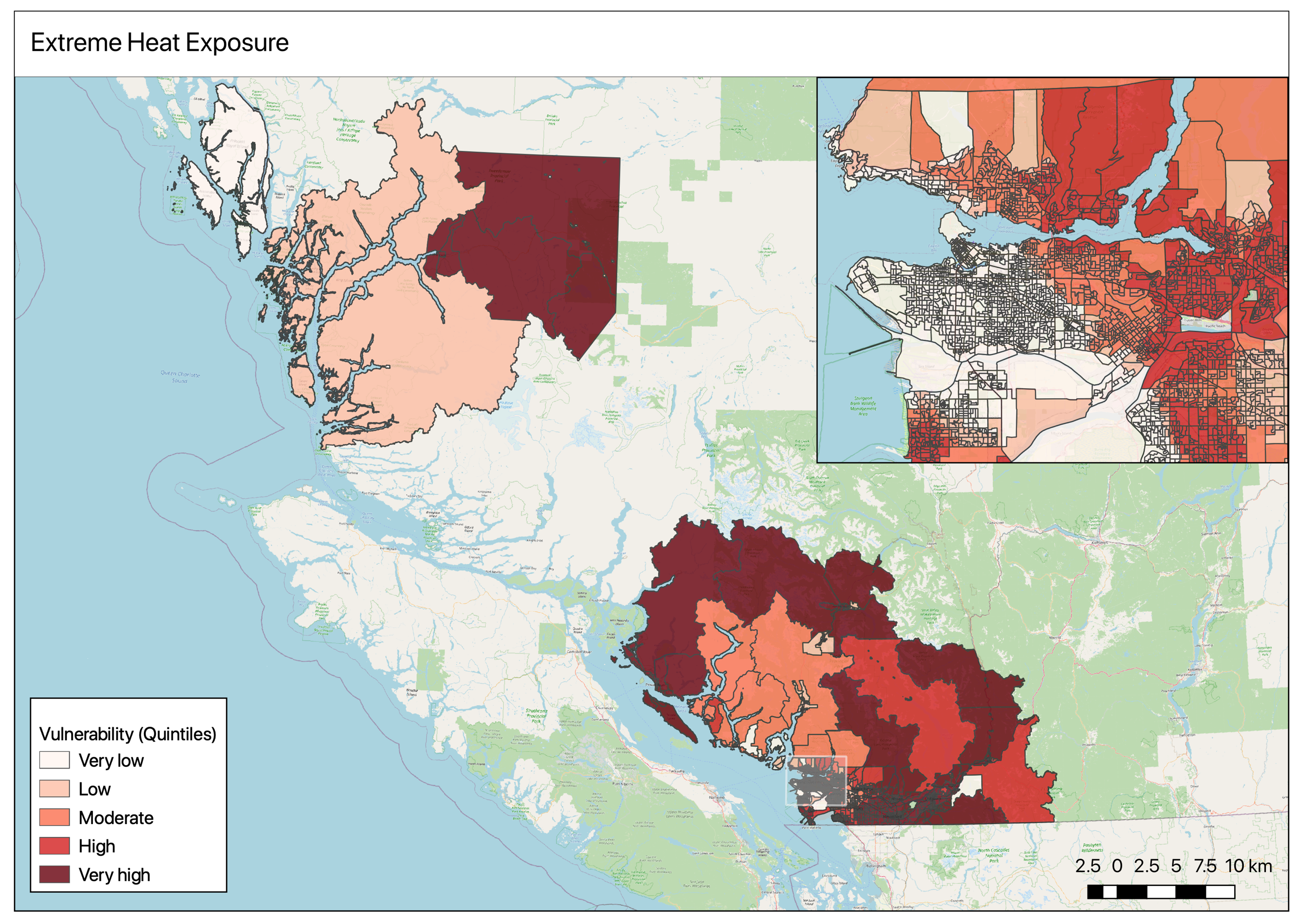
**

**
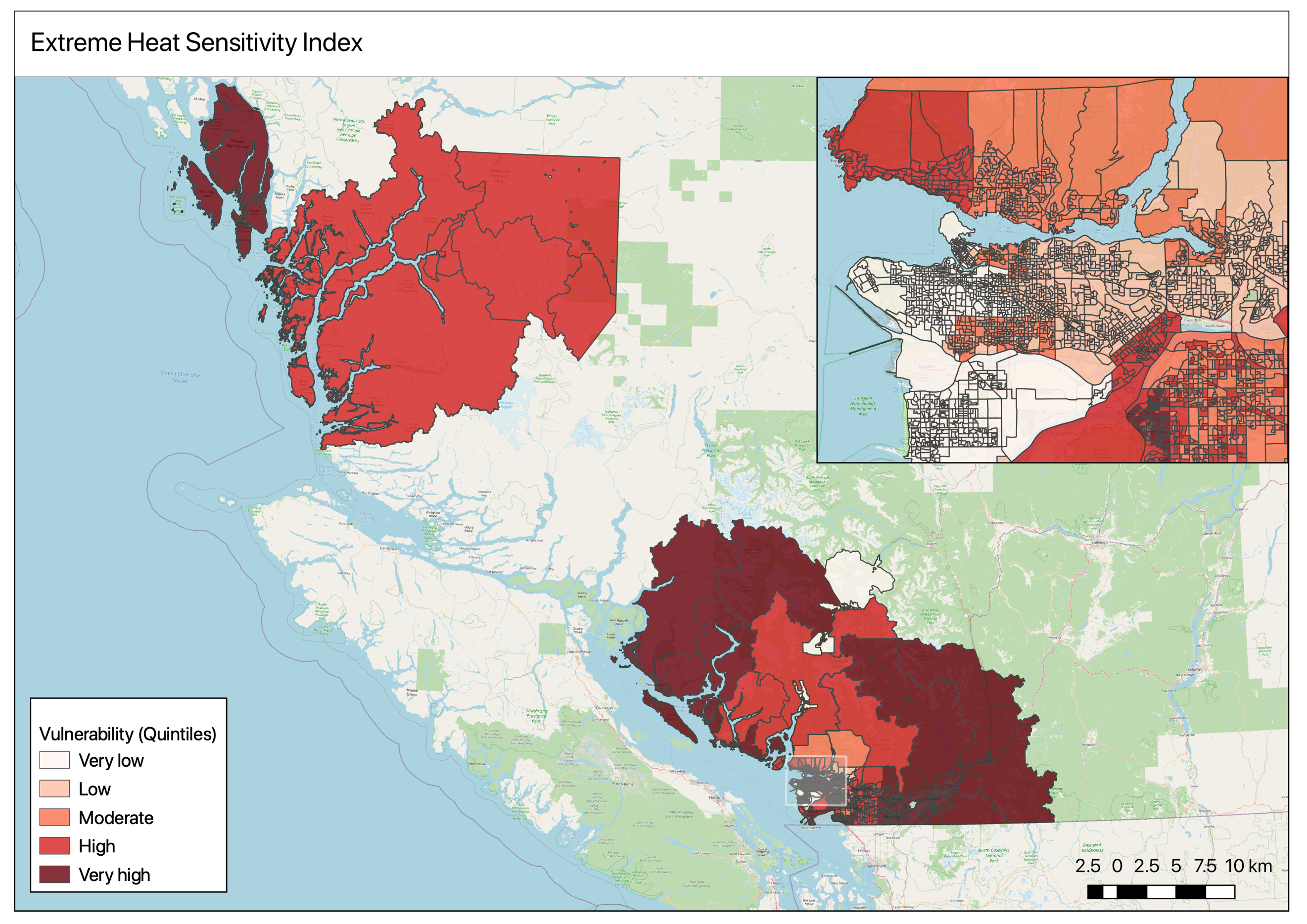
**

**
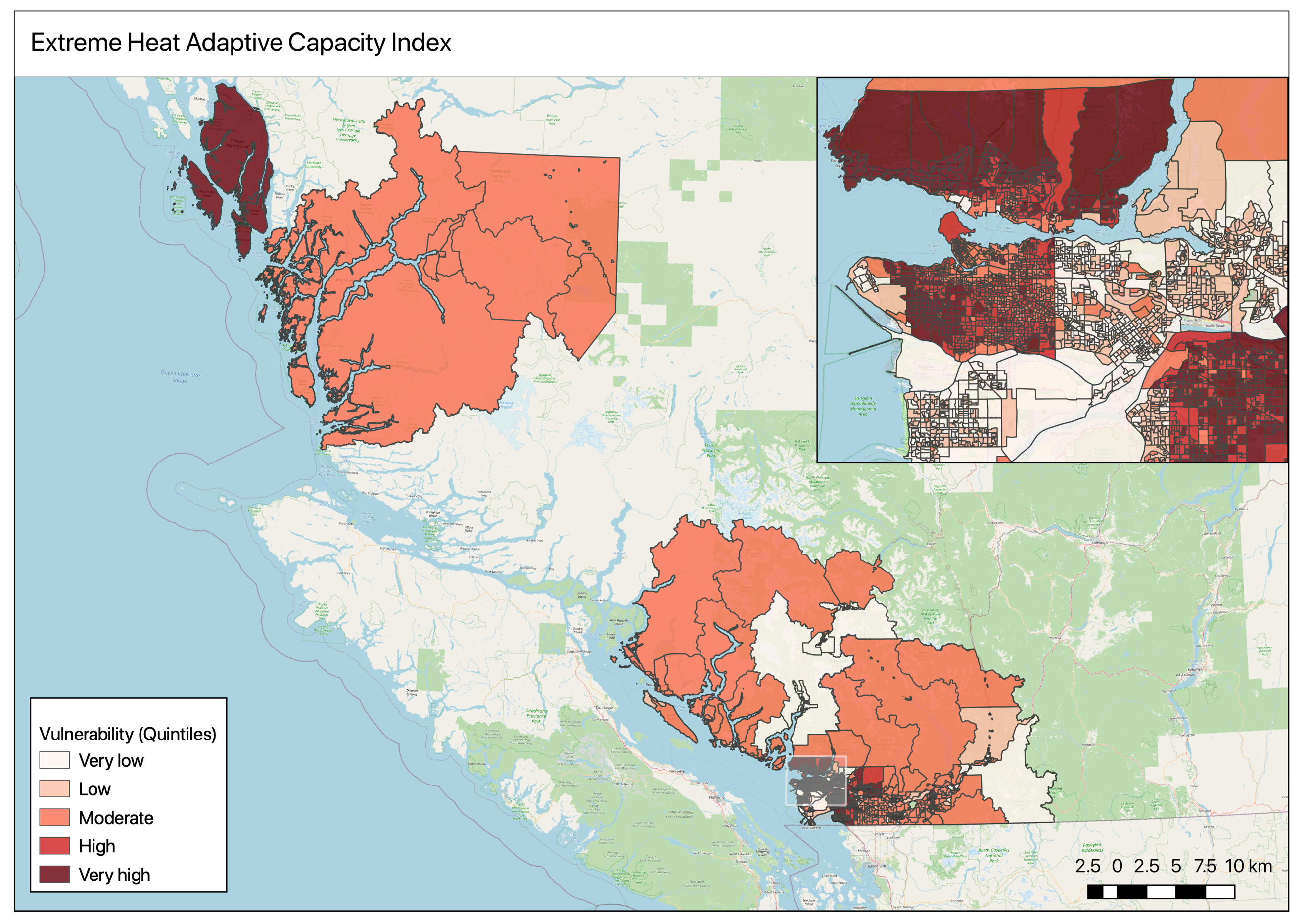
**

**
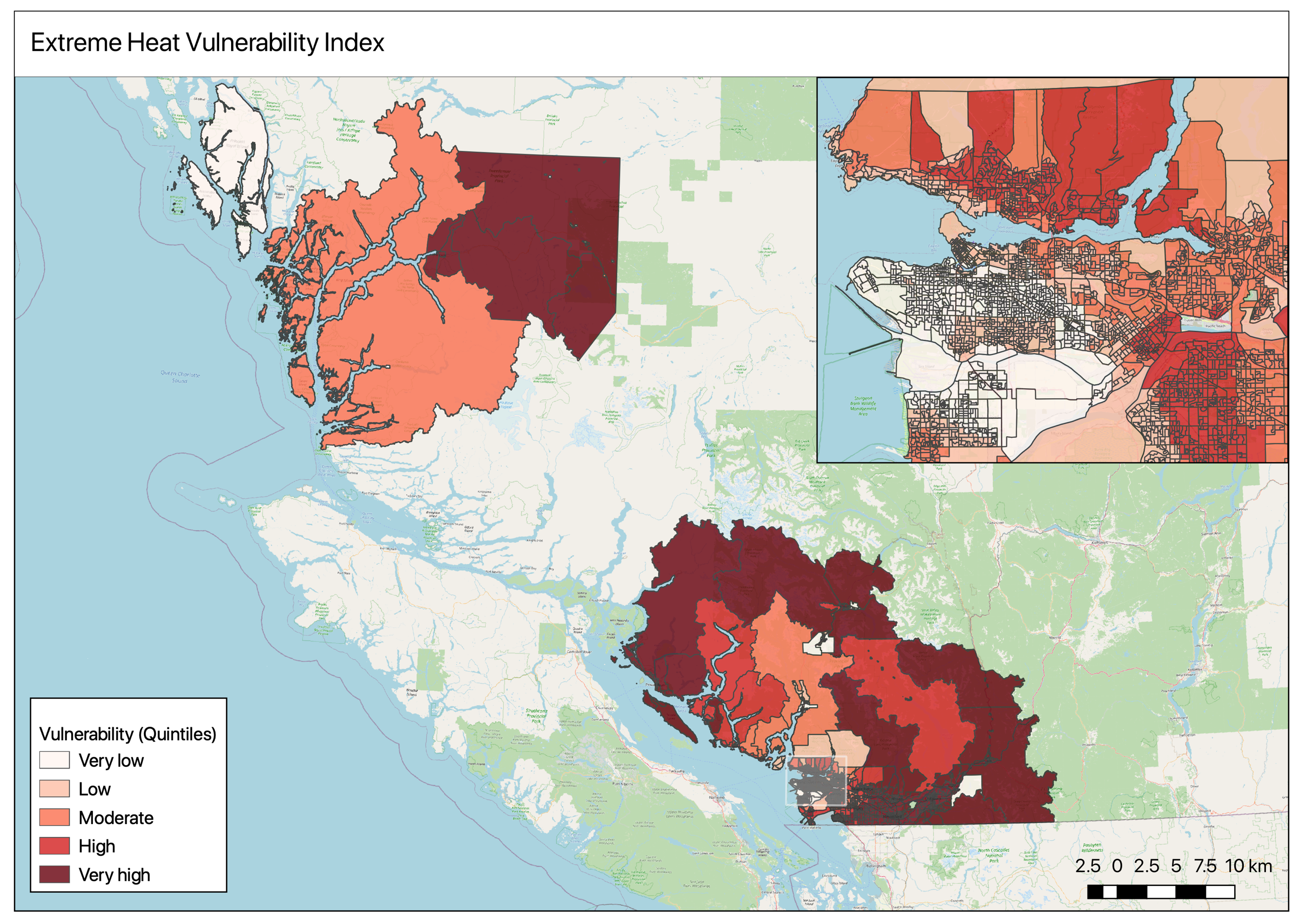
**

**Flooding**

**
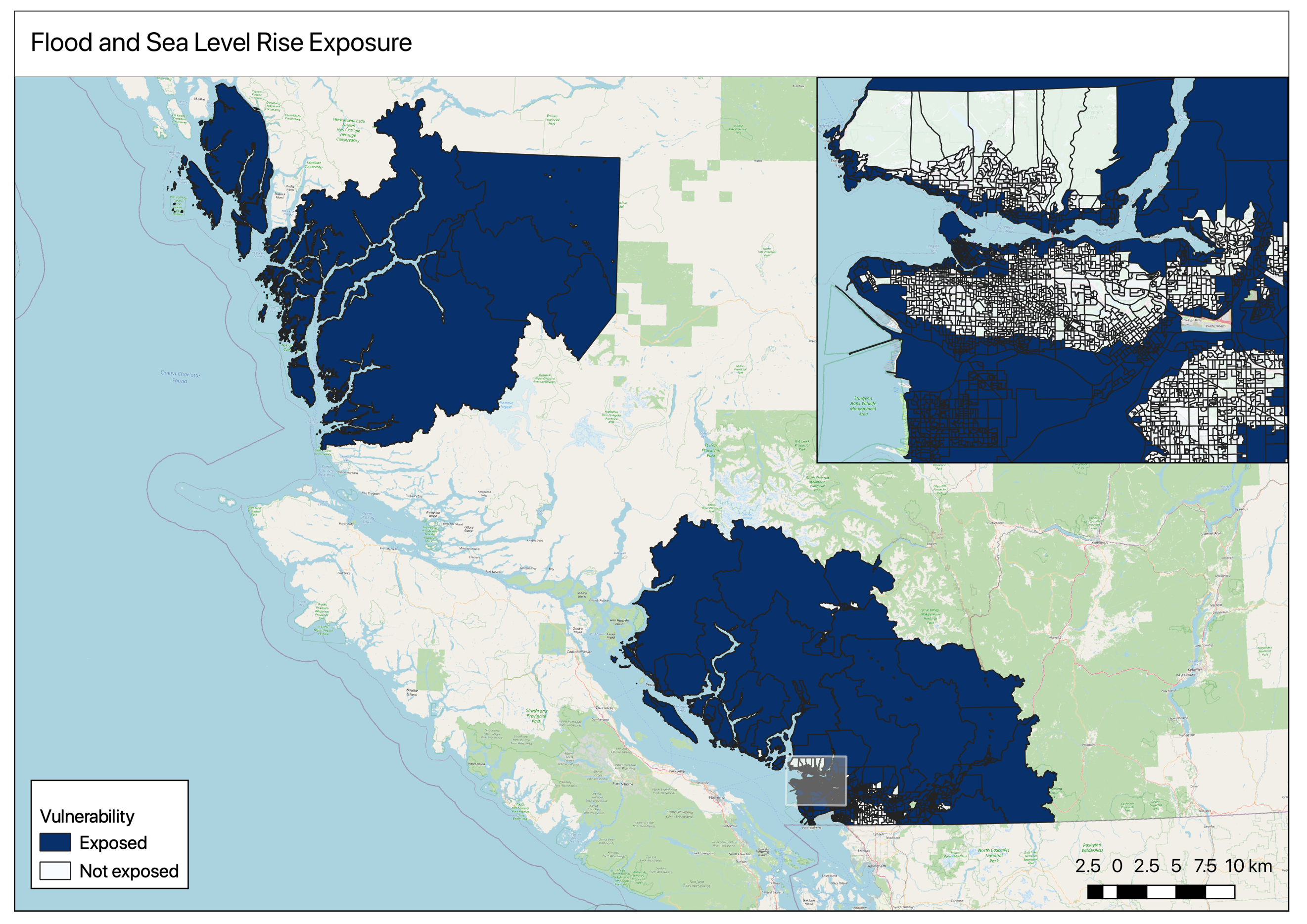
**

**
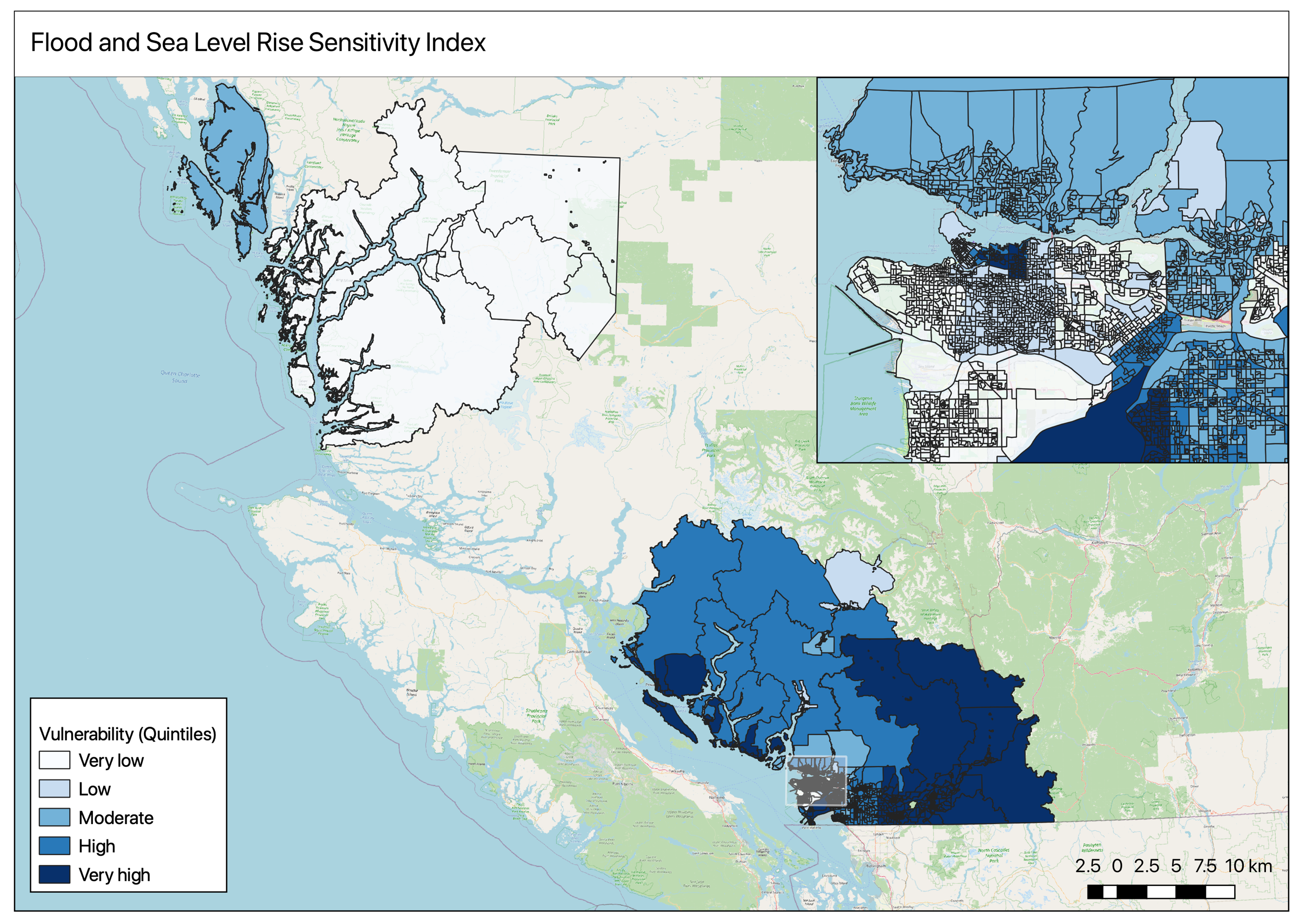
**

**
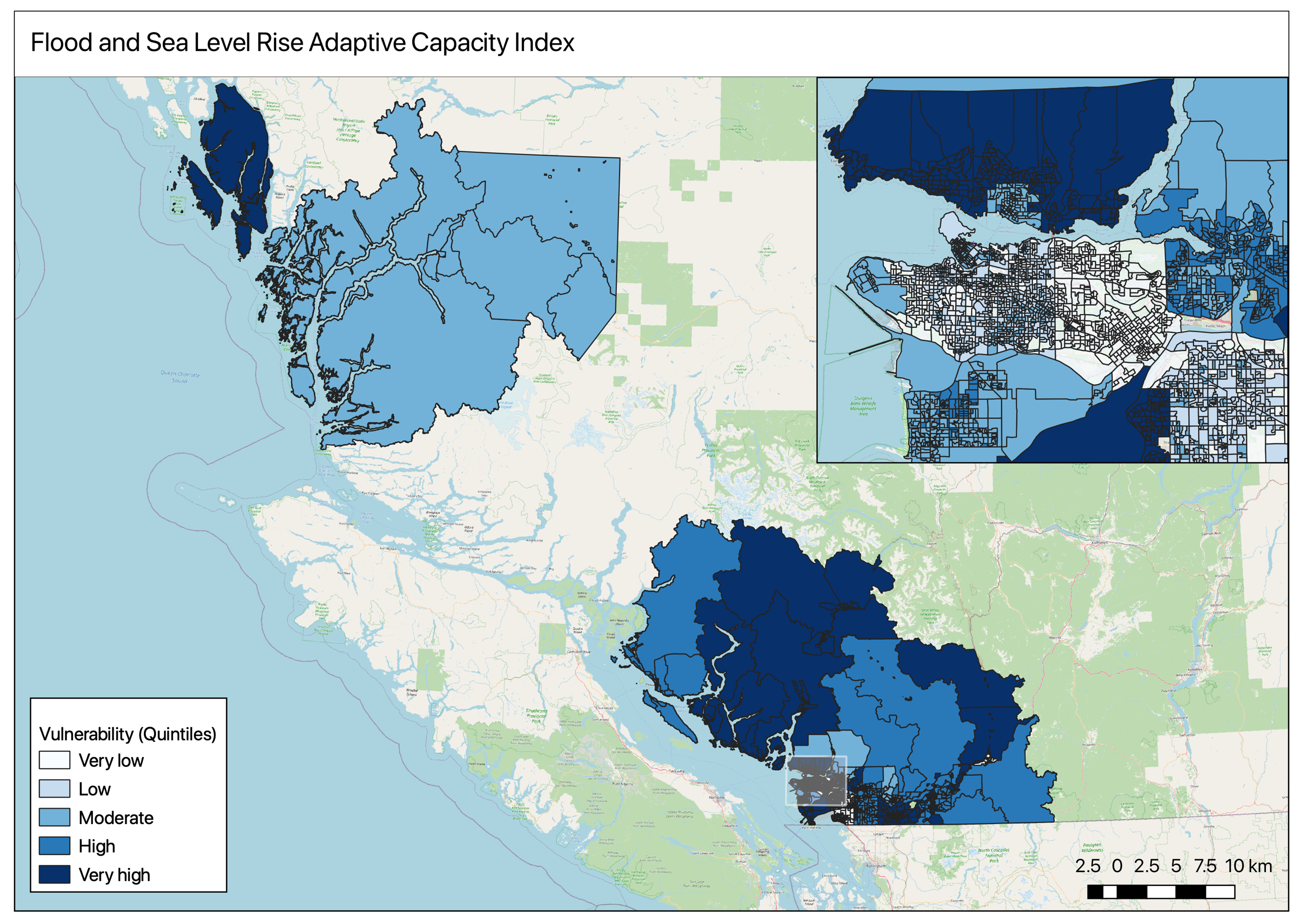
**

**
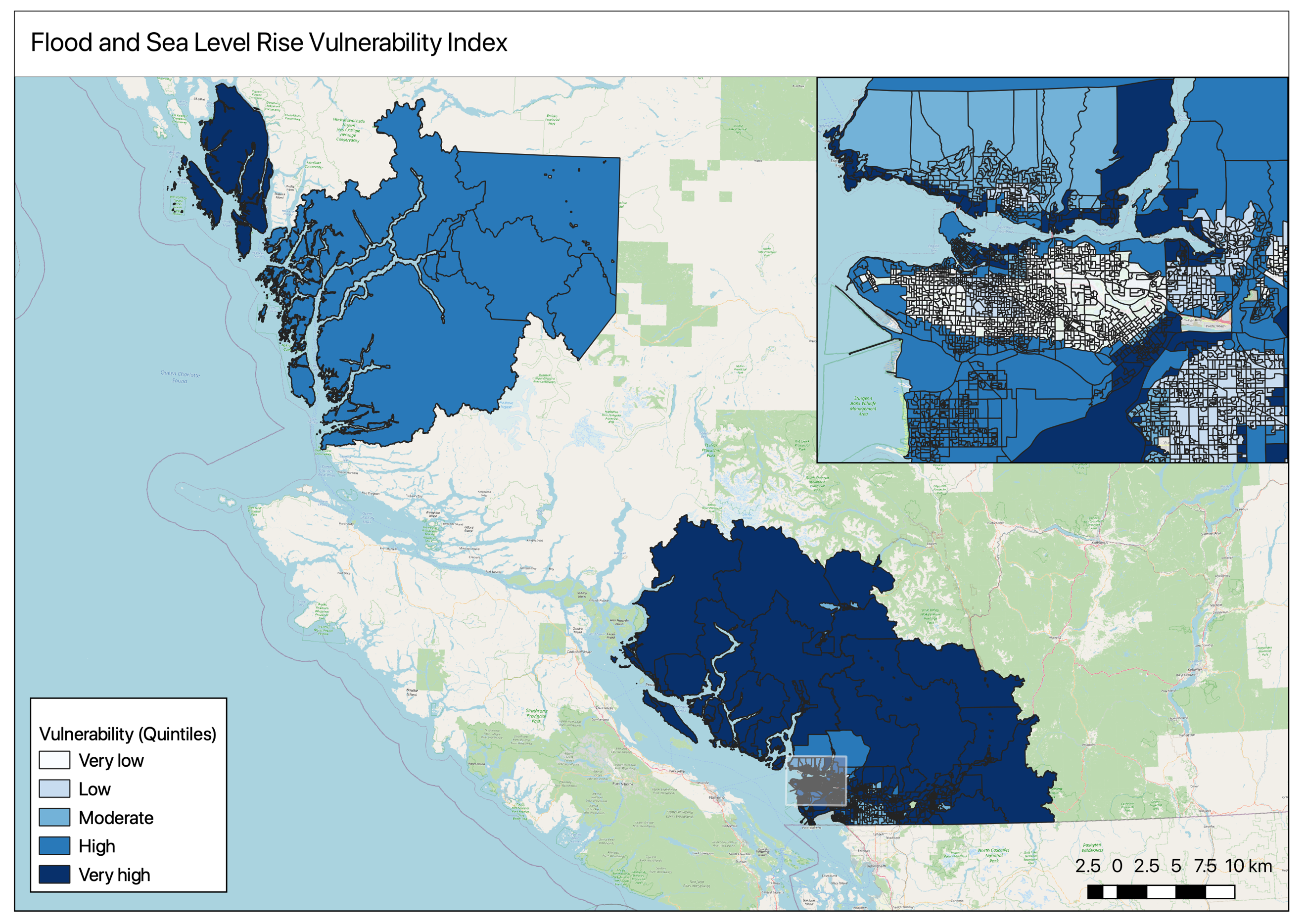
**

**Wildfire smoke**

**
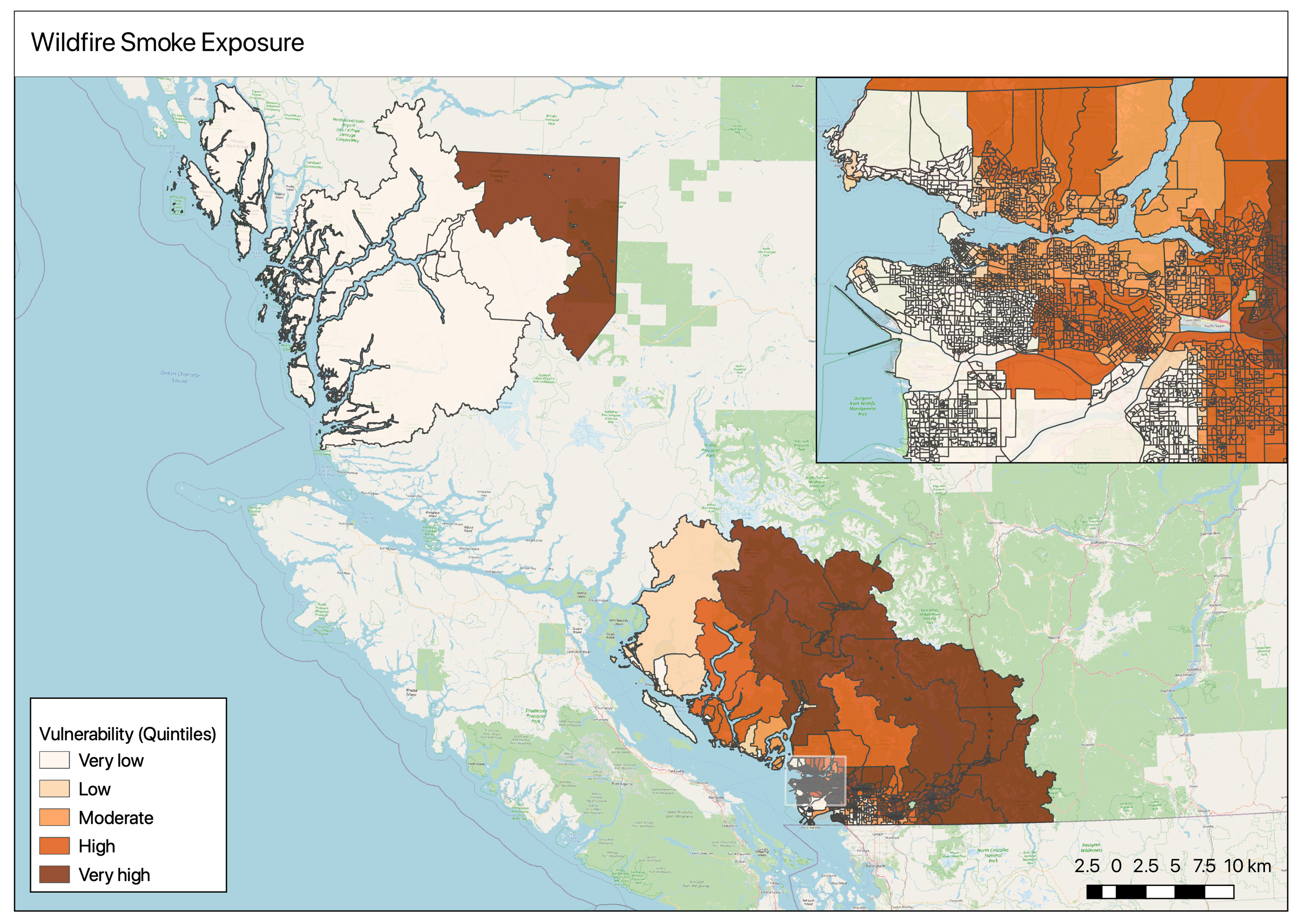
**

**
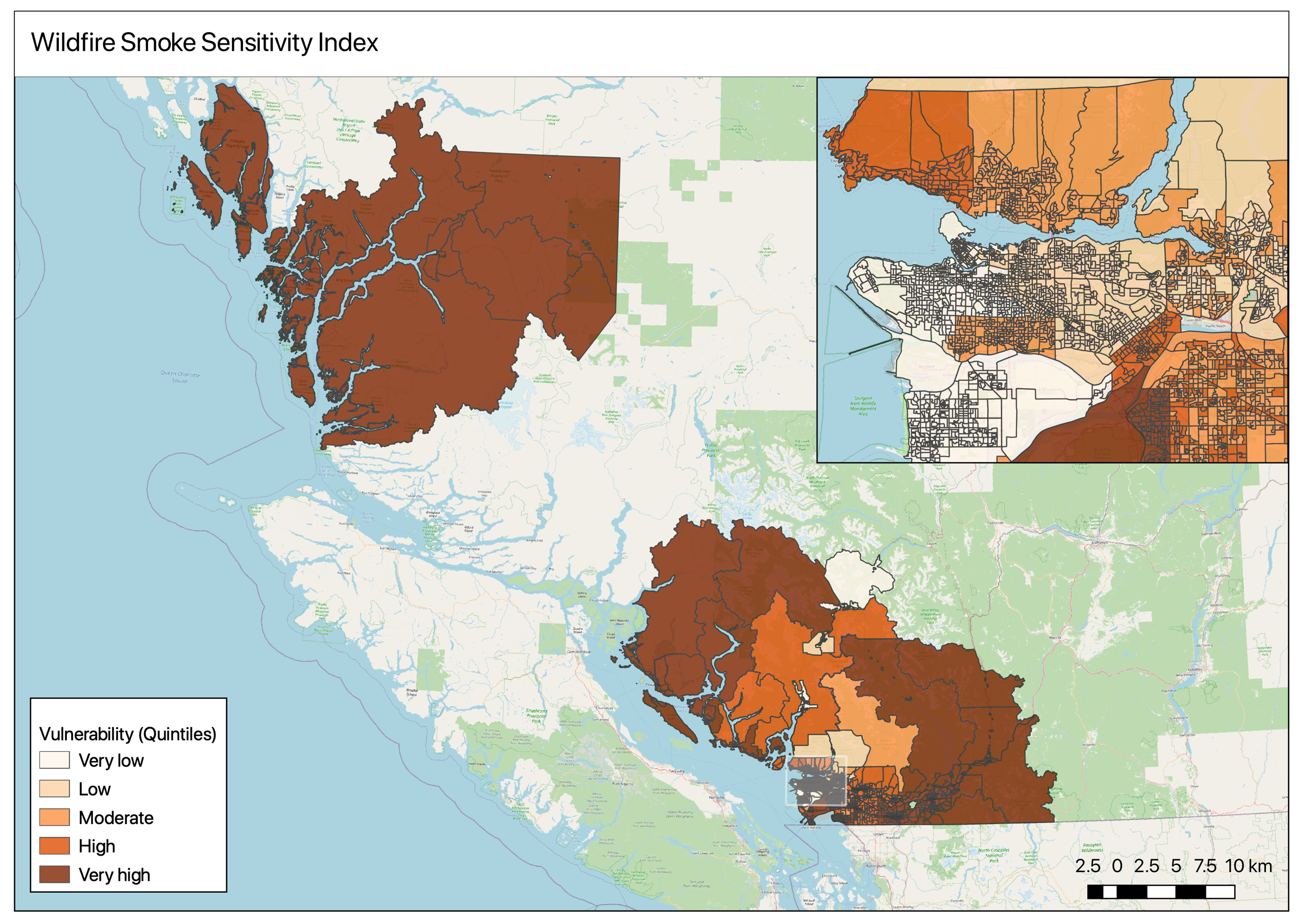
**

**
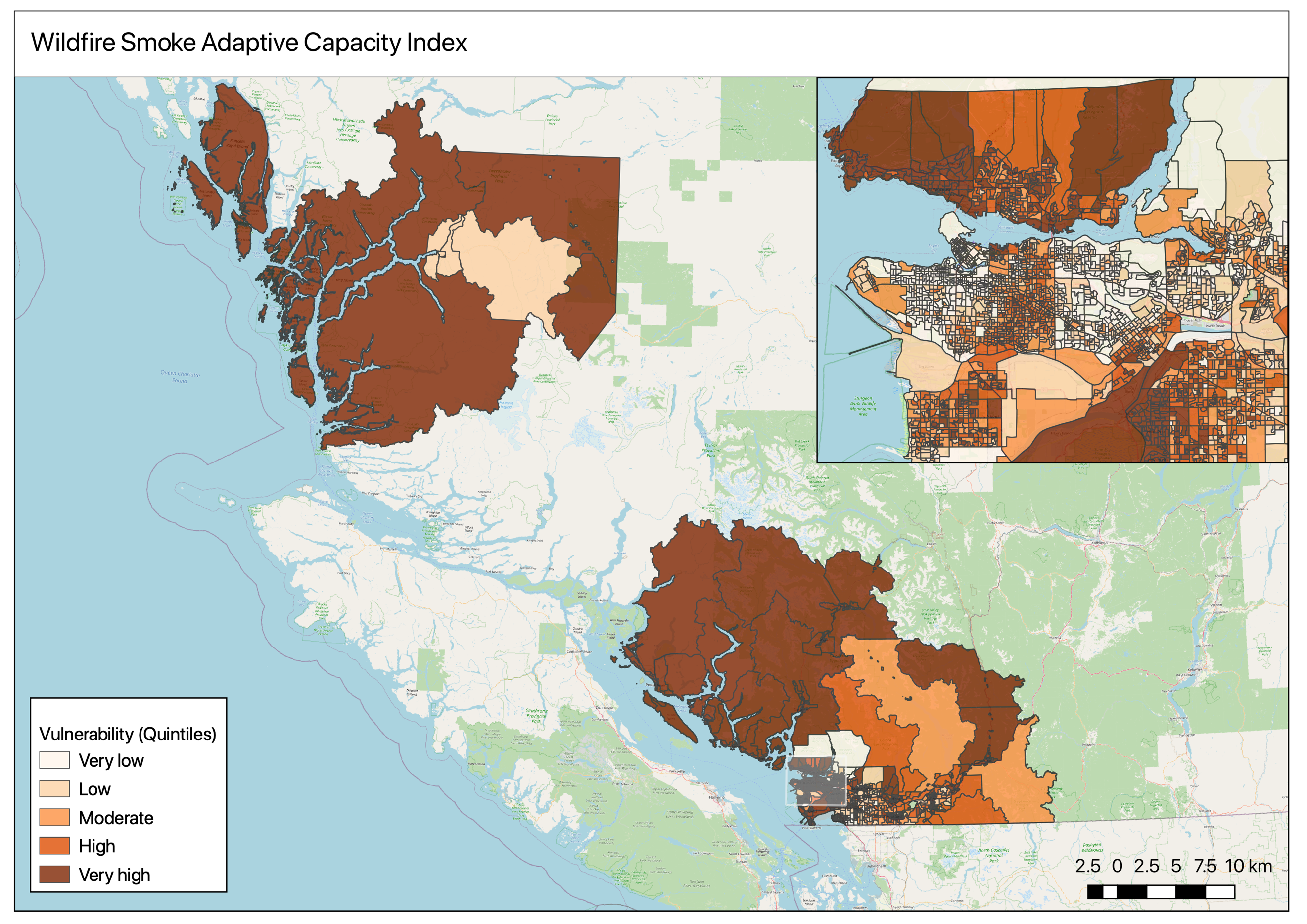
**

**
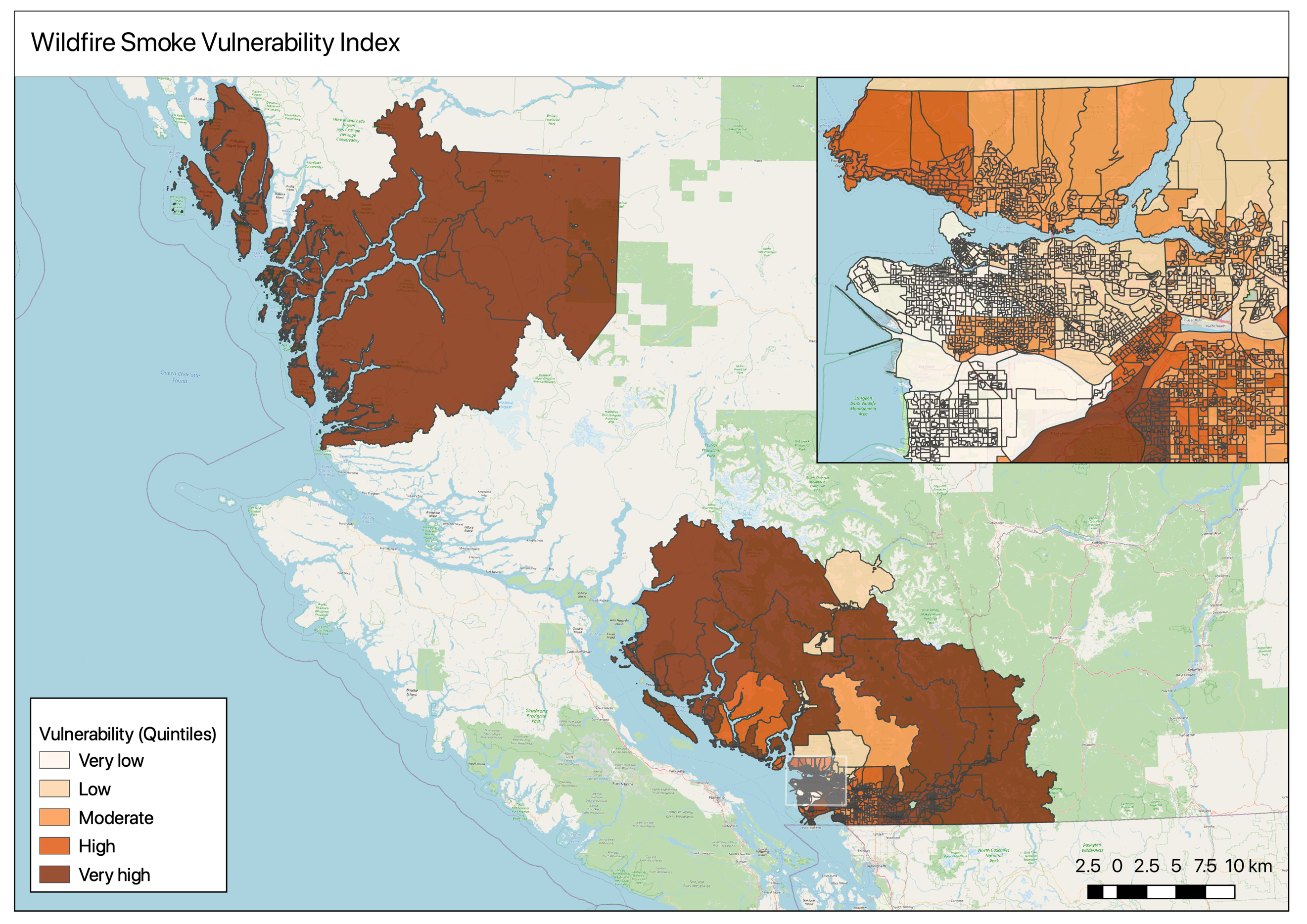
**

**Ground-level ozone**

**
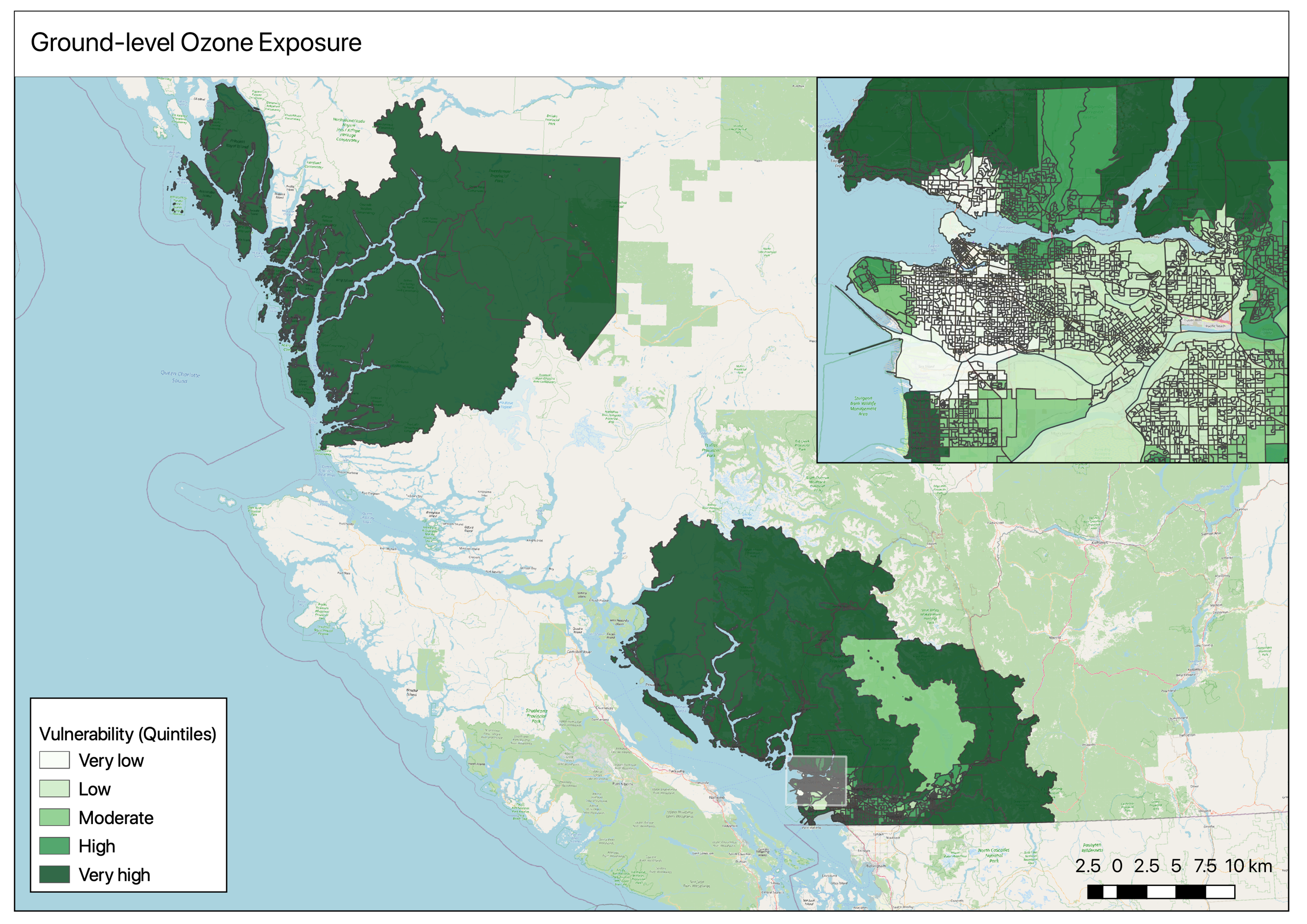
**

**
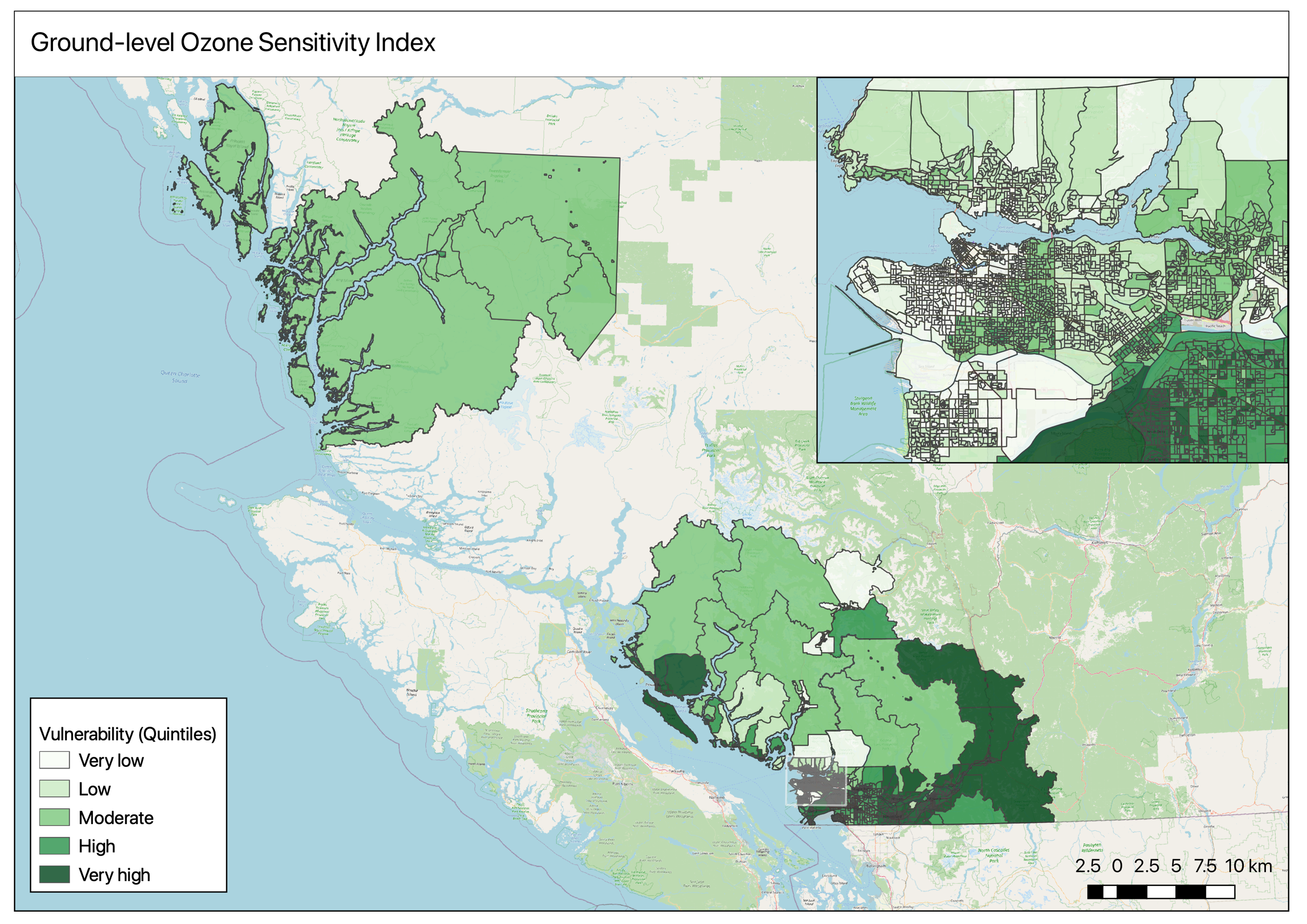
**

**
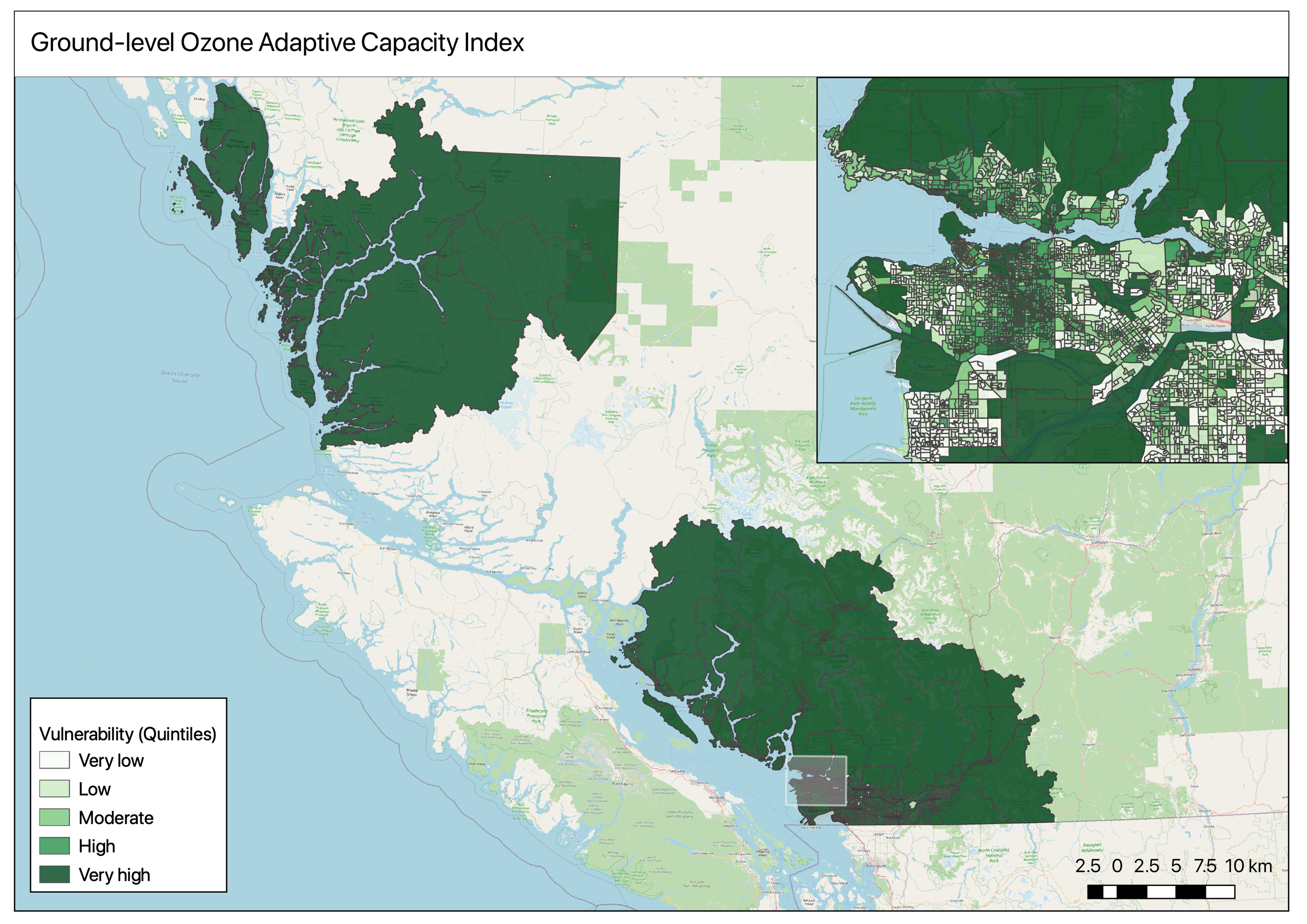
**

**
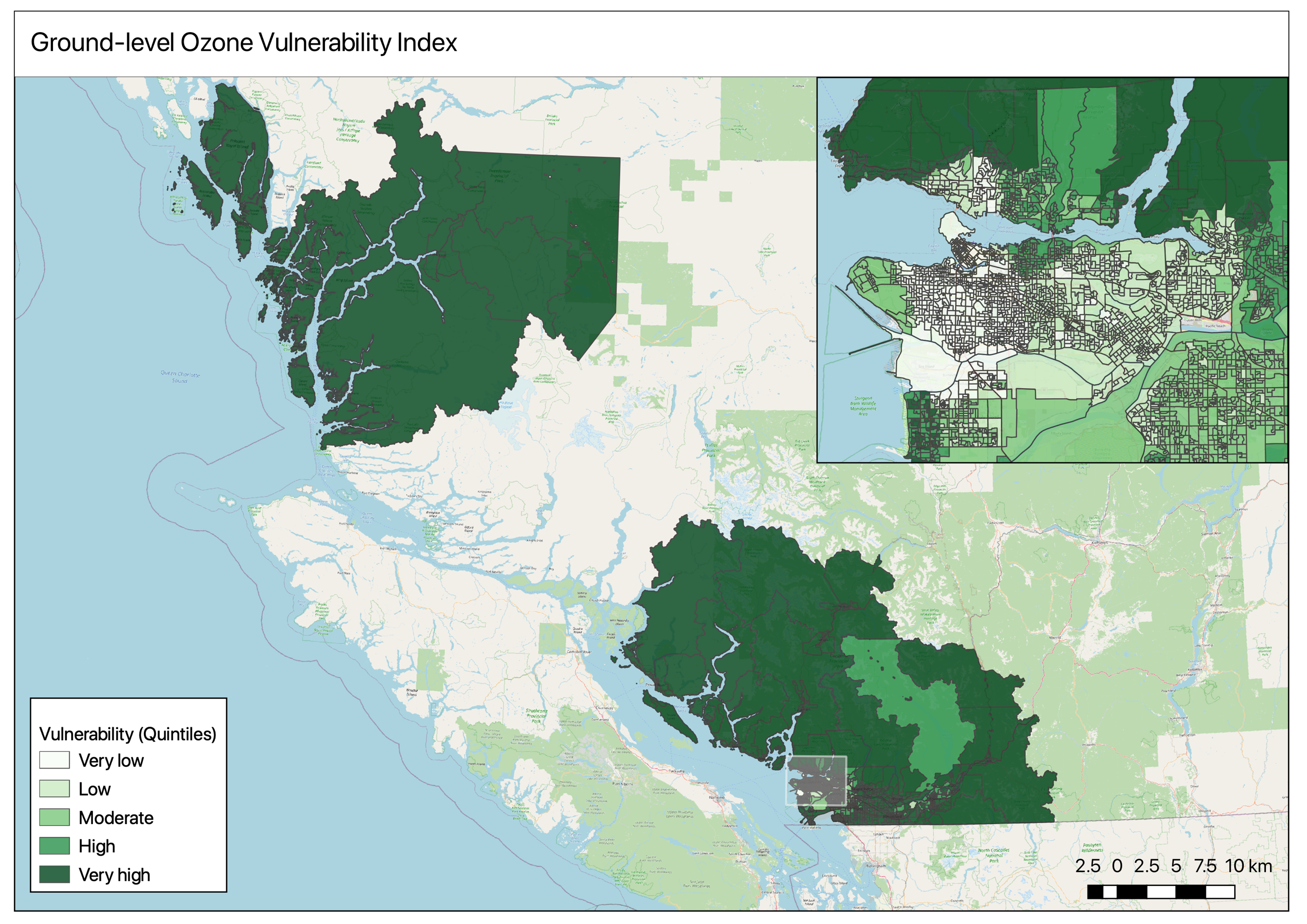
**
