# Supplementary material for: Geospatial indicators of exposure, sensitivity, and adaptive capacity to assess neighbourhood variation in vulnerability to climate change-related health hazards
Source: Environ Health. 2021 Mar 22;20:31. doi: 10.1186/s12940-021-00708-z (PMC7986027; doi:10.1186/s12940-021-00708-z)

**Additional file 6 – Identifying prioritised areas by combining categorical index scores**

**Figure 8:** Example of priority areas with high heat exposure and very high sensitivity (~608 DAs)

**
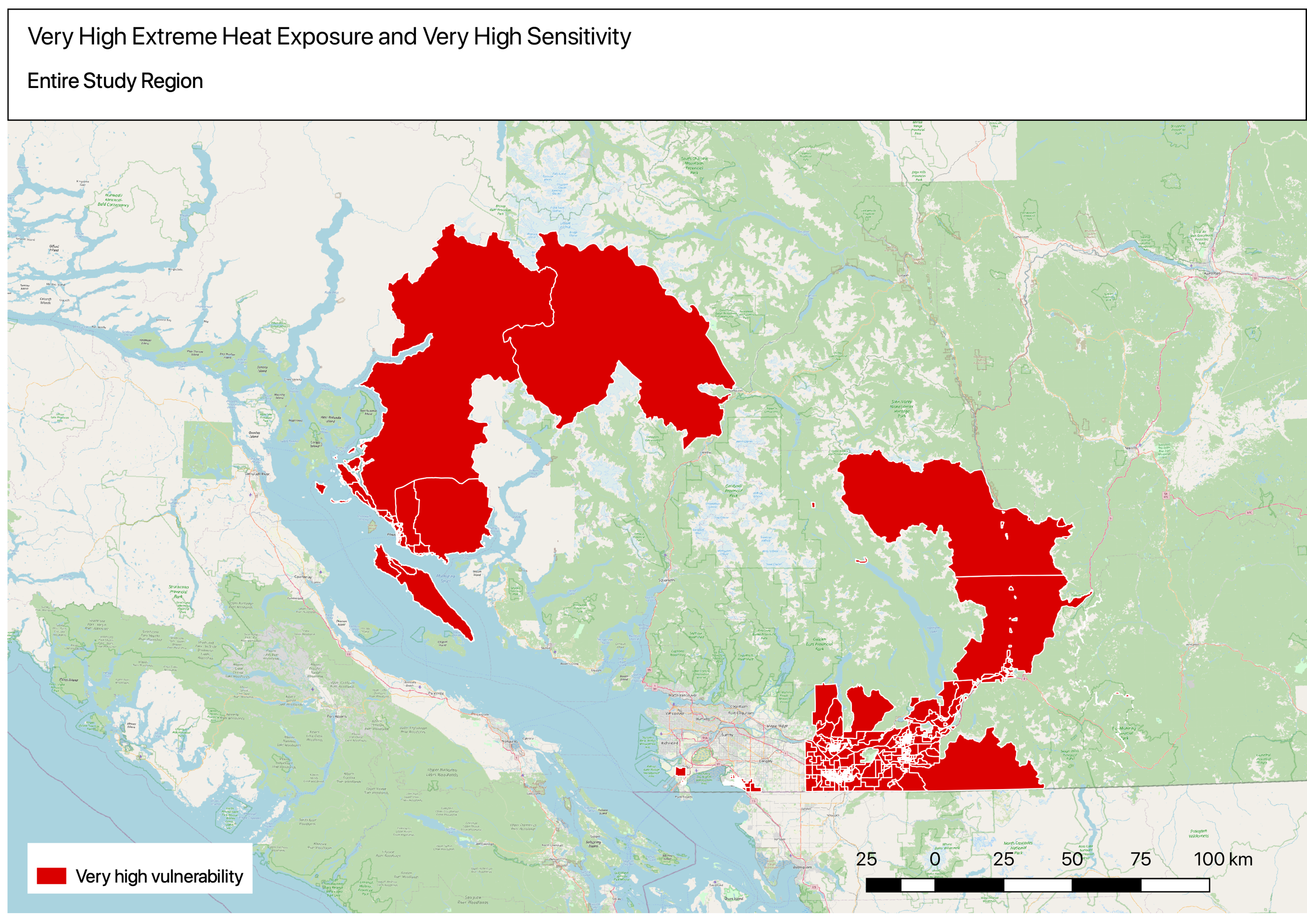
**

**Figure 9:** High flood exposure that are less vulnerable because of very high adaptive capacity (~203 DAs)


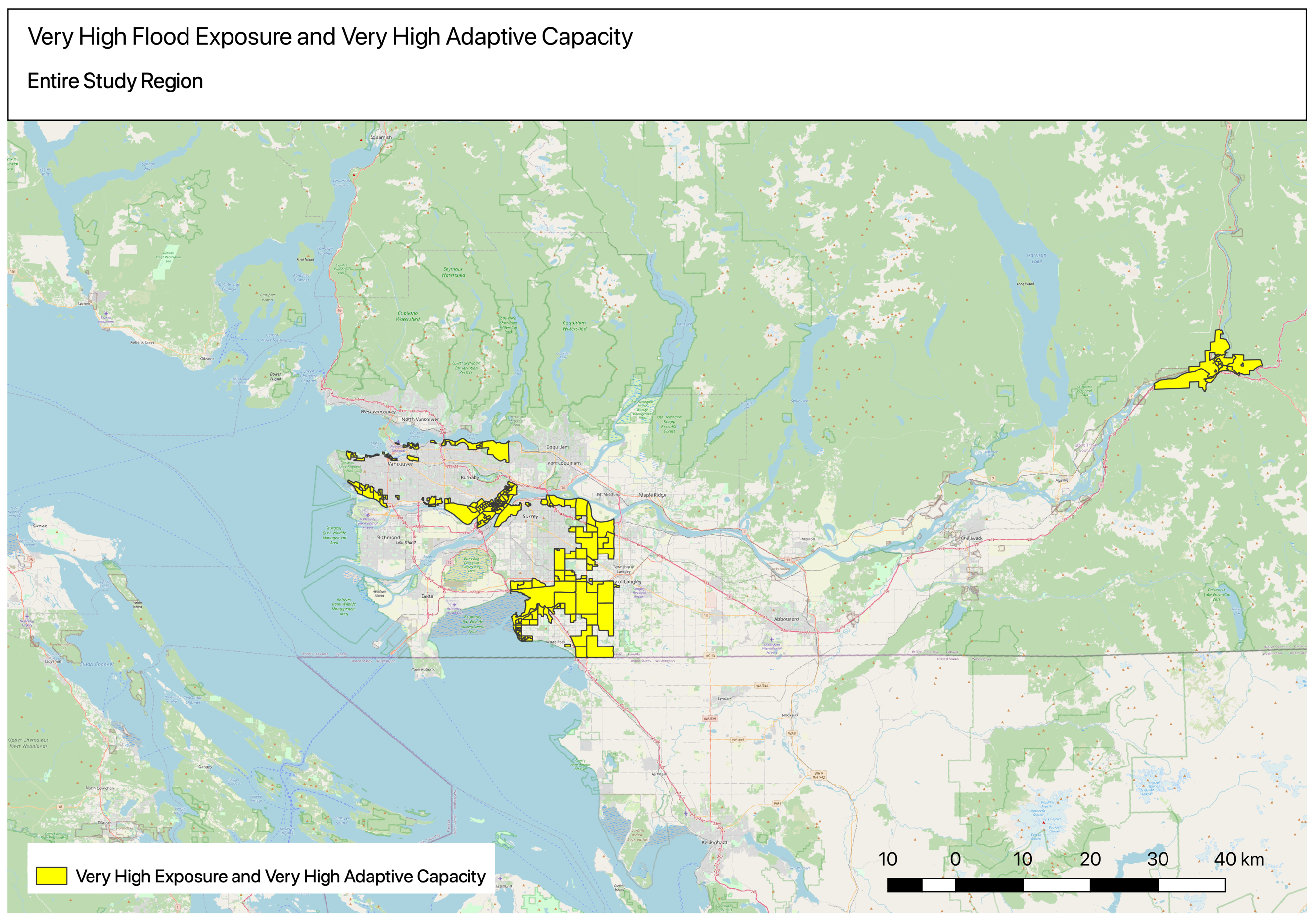


**Figure 10:** Highest vulnerability - very high smoke exposure and sensitivity and very low adaptive capacity (72 DAs)

**
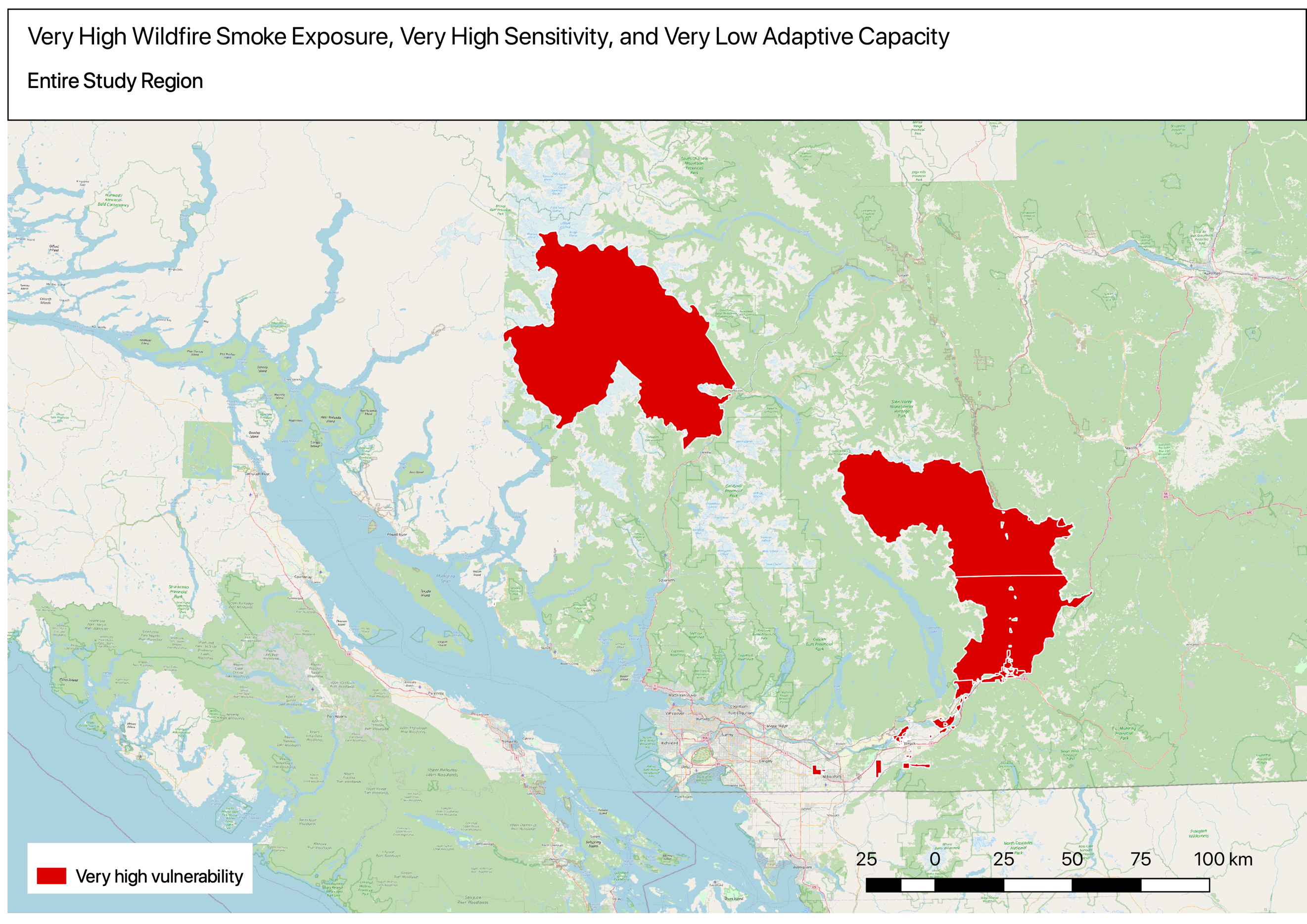
**

**Figure 11:** Lowest vulnerability - very low ozone exposure and sensitivity and very high adaptive capacity (~21 DAs)


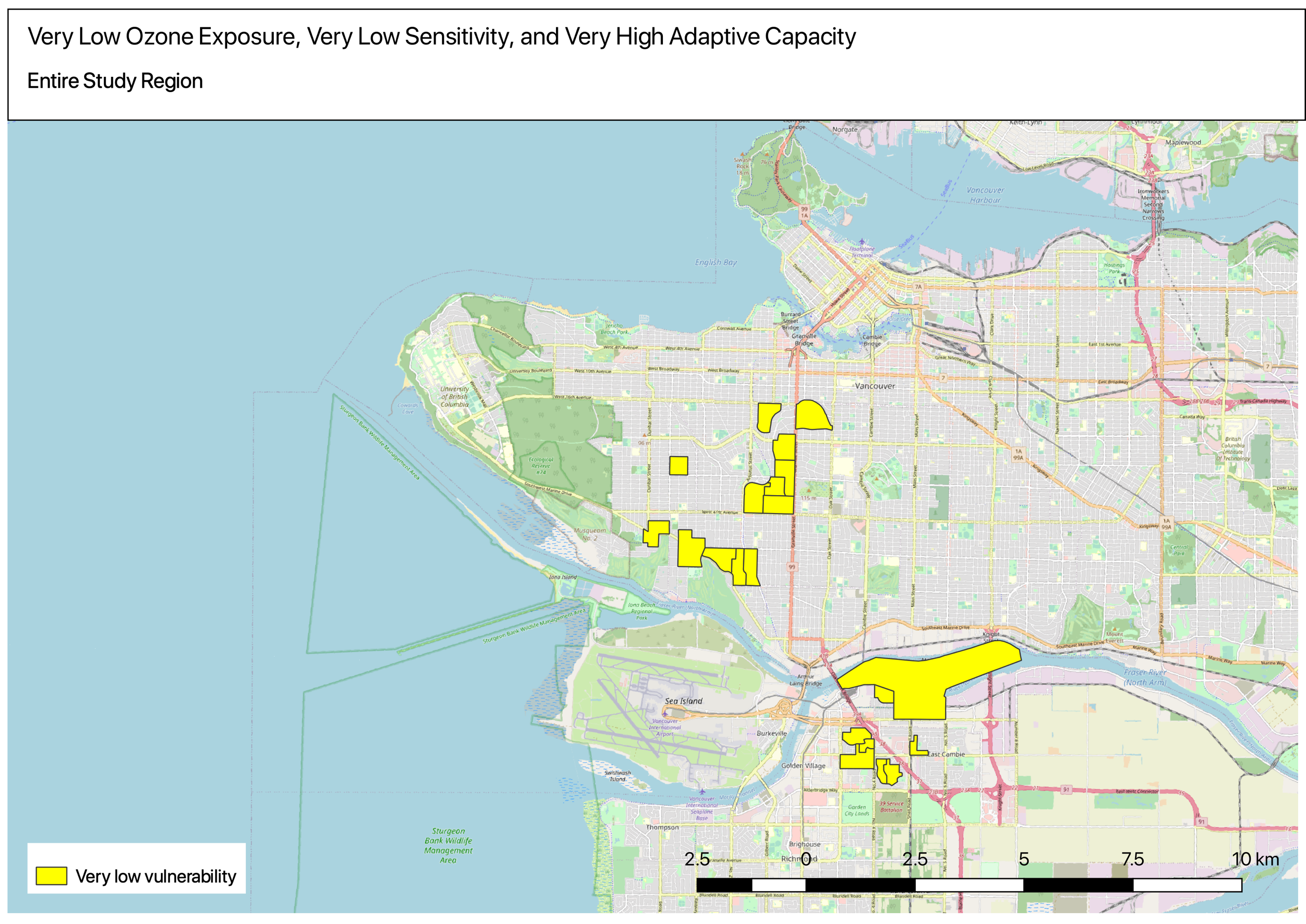

Supplement: Supplementary file 6 — Additional file 6. [file 12940_2021_708_MOESM6_ESM.docx]
